# Phylogenomic analyses of Crassiclitellata support major Northern and Southern Hemisphere clades and a Pangaean origin for earthworms

**DOI:** 10.1186/s12862-017-0973-4

**Published:** 2017-05-30

**Authors:** Frank E. Anderson, Bronwyn W. Williams, Kevin M. Horn, Christer Erséus, Kenneth M. Halanych, Scott R. Santos, Samuel W. James

**Affiliations:** 1 Department of Zoology, Southern Illinois University, Carbondale, IL 62901 USA; 2 North Carolina Museum of Natural Sciences, Research Laboratory, Raleigh, North Carolina 27699 USA; 3 Department of Biological and Environmental Sciences, University of Gothenburg, 405 30 Göteborg, SE Sweden; 4 Molette Biology Laboratory for Environmental and Climate Change Studies, Department of Biological Sciences, Auburn University, Auburn, AL 36849 USA; 5 Department of Biology, University of Iowa, Iowa City, Iowa, 52242 USA

**Keywords:** Clitellata, Crassiclitellata, Earthworm, Phylogenomics

## Abstract

**Background:**

Earthworms (Crassiclitellata) are a diverse group of annelids of substantial ecological and economic importance. Earthworms are primarily terrestrial infaunal animals, and as such are probably rather poor natural dispersers. Therefore, the near global distribution of earthworms reflects an old and likely complex evolutionary history. Despite a long-standing interest in Crassiclitellata, relationships among and within major clades remain unresolved.

**Methods:**

In this study, we evaluate crassiclitellate phylogenetic relationships using 38 new transcriptomes in combination with publicly available transcriptome data. Our data include representatives of nearly all extant earthworm families and a representative of Moniligastridae, another terrestrial annelid group thought to be closely related to Crassiclitellata. We use a series of differentially filtered data matrices and analyses to examine the effects of data partitioning, missing data, compositional and branch-length heterogeneity, and outgroup inclusion.

**Results and discussion:**

We recover a consistent, strongly supported ingroup topology irrespective of differences in methodology. The topology supports two major earthworm clades, each of which consists of a Northern Hemisphere subclade and a Southern Hemisphere subclade. Divergence time analysis results are concordant with the hypothesis that these north-south splits are the result of the breakup of the supercontinent Pangaea.

**Conclusions:**

These results support several recently proposed revisions to the classical understanding of earthworm phylogeny, reveal two major clades that seem to reflect Pangaean distributions, and raise new questions about earthworm evolutionary relationships.

**Electronic supplementary material:**

The online version of this article (doi:10.1186/s12862-017-0973-4) contains supplementary material, which is available to authorized users.

## Background

“*The plough is one of the most ancient and most valuable of man's inventions; but long before he existed the land was in fact regularly ploughed, and still continues to be thus ploughed by earth-worms. It may be doubted whether there are many other animals which have played so important a part in the history of the world, as have these lowly organised creatures.*”

Charles Darwin, *The formation of vegetable mould through the actions of worms, with observations on their habits*, pg. 313 [1]

Earthworms (Crassiclitellata) constitute a diverse group of primarily terrestrial, burrowing annelids comprising 6000+ extant species in 18 families and found on all continents except Antarctica. Most earthworm species live in soil, but some live in decaying logs, leaf litter, stream mud and riverbanks, as well as arboreal (e.g., epiphytic root masses) and even marine littoral habitats. Charles Darwin famously extolled the importance of earthworms as terrestrial ecosystem engineers, churning and aerating the soil with their burrows as well as burying and processing large fragments of organic matter and making their nutrients available to plants. Large-scale engineering by earthworms has recently been documented in South America [2] and may occur elsewhere. Even apart from their direct agricultural importance as soil processors, earthworms have a substantial economic impact—epigeic (leaf litter/compost-dwelling) species are used to process food waste (vermiculture), larger species are sold as bait for fish, and some earthworm species are considered delicacies and are sold for human consumption. Earthworms are prey items for many other species, including planarians, leeches, mollusks, insects, amphibians, lizards, snakes, birds and mammals, and thus serve as a crucial link in numerous terrestrial food webs. Many earthworms are considered invasive; approximately one-third of all earthworm species in North America are introduced from Europe and Asia [3, 4]. As invasive earthworms spread in recently glaciated and otherwise earthworm-free forests in North America, they affect many microbial, plant and invertebrate species that have come to rely on large amounts of undisturbed leaf material [5].

Widespread distribution and limited dispersal abilities make earthworms a promising model of historical biogeographic patterns at a global scale. Indeed, speculation about earthworm biogeography has a long history, perhaps unusually attractive to history-of-science enthusiasts. Early ideas about earthworm distributions relied on dubious land bridge hypotheses (review in [6]). Apocryphal lore has it that J.W. Michaelsen (a great Clitellata taxonomist of the late 19th and early 20th centuries; e.g., [7]) and Alfred Wegener were office neighbors in Hamburg, Germany for a time. Michaelsen [8] cited Wegener’s hypothesis of continental drift [9] as providing considerable explanatory power for the distributions of earthworms, and named an amphi-Atlantic genus after him (*Wegeneriella* Michaelsen 1933). Despite Michaelsen’s contribution, speculation about land bridges continued to pervade the earthworm biogeographic literature.

Earthworms have a very poor fossil record, and specialists have long disagreed about directions of character evolution within the group. Early earthworm phylogenies were highly intuitive (cf. [10]) and shed little light on earthworm historical biogeography. Earthworm phylogenetic understanding has progressed slowly since these initial attempts. The few applications of cladistic analysis, such as Jamieson’s (1988) morphological study [11], yielded mixed conclusions, and the first use of molecular data [12] overturned many of the morphology-based hypotheses. James and Davidson [13] included a broader gene (16S, 18S and 28S ribosomal RNA genes) and taxon sampling of Crassiclitellata and several outgroups and were able to reinterpret many morphological changes defining the families of crassiclitellates, proposing new hypotheses of morphological evolution and rehabilitating older ones.

Although James and Davidson [13] clarified many aspects of earthworm phylogeny, relationships among several major groups remain poorly supported. Fortunately, the advent of low-cost, high-throughput sequencing methods has revolutionized the study of higher-level relationships across the tree of life, allowing researchers to bring dozens to thousands of genes to bear on previously intractable questions. To test previous hypotheses of relationships among earthworms and provide a robust framework for historical biogeographic inference and studies of character evolution, we generated transcriptomic data from representatives of nearly all major extant lineages of Crassiclitellata and performed a series of analyses to infer relationships among the major lineages of earthworms.

## Methods

### Taxon sampling

A total of forty taxa (33 crassiclitellates, one moniligastrid and six outgroup taxa) were sampled for this study (Table 1). James and Davidson [13] used representatives of several clitellate taxa as outgroups for their analysis of crassiclitellate phylogeny based on 18S data, but only used an enchytraeid for most other analyses (including combined analyses of multiple loci). Their 18S Bayesian phylogeny (Fig. 1 in [13]) suggested that Haplotaxidae s. str. (represented by *Haplotaxis gordioides*) was sister to Metagynophora (Crassiclitellata + Moniligastridae), and our preliminary analyses of a broader sample of clitellate transcriptomes also suggested that members of Haplotaxidae are the closest extant relatives of Metagynophora (not shown). Haplotaxidae, with its currently recognized eight genera, is no longer considered to be monophyletic and has long been regarded as a “dustbin” for slender, primitive-looking clitellates [10, 14–17]. We chose representatives of four haplotaxid species, Lumbriculidae (*Lumbriculus variegatus*) and Propappidae (*Propappus volki*) as outgroups; *P*. *volki* was used to root the phylogeny. No leeches or branchiobdellidans were used in this study, for two reasons. First, previous work [13, 18] and preliminary analyses including several leech and branchiobdellidan transcriptomes supported a clade comprising Lumbriculidae, Branchiobdellida and Hirudinea. Second, all available leech and branchiobdellidan transcriptomes showed appreciably longer branch lengths on preliminary ML trees than did all other clitellates. Sampling only the relatively short-branch *Lumbriculus variegatus* allows this outgroup clade to be represented while avoiding potential confounding factors due to branch-length heterogeneity.

**Table 1:** Collection locality, museum location of voucher specimen, museum catalog number, SRA project number, number of Illumina reads, number of Trinity contigs and number of HaMSTr ortholog groups represented for each of the thirty-seven transcriptomes generated in this study

Taxon	Locality	Museum	# Contigs	# HaMStR Orthologs
Acanthodrilidae sp.	Argentina, Tierra del Fuego, Ushuaia (coll. E. Lapied)	NCSM 27264	181228	1140
*Alma* sp. Almidae	Gabon, edge of Lac Vembo, Gamba complex, (coll. S James 18 May 2008)	NCSM 27265	110015	558
*Avelona ligra* Lumbricidae	France, Jargeau, Loiret Department, (coll. M. Koken)	MNHN XXXXXX	182509	1173
*Criodrilus lacuum* Criodrilidae	Hungary (coll. C. Csuzdi)	NCSM 27266	119084	934
*Dendrobaena hortensis* Lumbricidae	Sweden, Södermanland, Vingåker, Valltrand, indoor compost, 59.0864 N, 16.0544 E (coll. E. Boräng, 1 Jan 2012)	SMNH 161291 in EtOH CE13942	179981	1180
*Dichogaster* sp. (green tree worm) Benhamiidae	Brazil, Amazonas, near Manaus, Reserva Campina (coll. S. James, S. Coral, 2 Feb 2012)	NCSM 27267	116065	1140
*Dichogaster* sp. Benhamiidae	France, Guadeloupe, Basse Terre (colls. S. James, F. Gamiette Feb 2013)	NCSM 27268	106438	1152
*Dichogaster saliens* Benhamiidae	France, Guadeloupe, Chutes Carbet, Basse Terre (colls. S. James, F. Gamiette Feb 2013)	NCSM 00000	98665	999
*Drawida* sp. Moniligastridae	USA, Tonganoxie, Kansas (coll. S. James? May 2013)	NCSM 27269	159219	1081
*Eisenia andrei* Lumbricidae	Unknown	---	137631	1217
*Eisenia andrei* Lumbricidae	Sweden, Södermanland, Vingåker, Valltrand, indoor compost, 59.0864 N, 16.0544 E (coll. E. Boräng, 1 Jan 2012)	SMNH 161292 in EtOH CE13945	168836	1191
*Eudrilus eugeniae* Eudrilidae	Brazil, Sao Paulo, bait shop (coll. S. James, 7 Nov 2010)	NCSM 27270	85990	1008
*Fimoscolex* sp. Glossoscolecidae	Brazil, Assistencia, São Paulo, Fazenda Sta Rosa (coll. S. James, 9 Nov 2012)	NCSM 27271	95465	705
*Gatesona chaetophora* Lumbricidae	France, Aveyron, L'Hospitalet du Larzac (coll. S James, 1 Mar 2011)	NCSM 27272	104334	961
*Geogenia benhami* Microchaetidae	South Africa, Western Cape, Stellenbosch (colls S. James, D. Plisko, 27 Aug 2011)	NCSM 27273	84303	932
*Glossodrilus* sp. Glossoscolecidae	Brazil, Amazonas, near Manaus, Reserva Ducke (colls. S. James, S. Coral 1 Feb 2012)	NCSM 27274	122993	1053
*Glossoscolex* sp. Glossoscolecidae	Brazil, Parana, Campina Grande do Sul, Caratuva peak trail (coll. S. James, M. Bartz, 17 Oct 2010)	NCSM 27275	58411	722
*Hemigastrodrilus monicae* Hormogastridae	France, Aveyron, L'Hospitalet du Larzac (coll. S James, 1 Mar 2011)	NCSM 27276	103338	1098
*Hormogaster elisae* Hormogastridae	SRA PRJNA196484*,Spain, El Molar, 40°44′22.9″N, 3°33′53.1″W	---	459282	1234
*Kerriona* sp. Graciosa1 Ocnerodrilidae	Brazil, Parana, Graciosa Road (coll. S. James, 4 Nov 2010)	NCSM 27277	104982	1010
*Komarekiona eatoni* Komarekionidae	USA, Sideling Hill Wildlife Mgmt. Area, Washington County, Maryland. (colls. S. James, M. Callaham, May 2013)	NCSM 27278	83743	1151
*Kynotus pittarelli* Kynotidae	Madagascar, Antsirabe, 19°46'38.60"S 47°06'41.69"E	NCSM 00000	108836	1073
*Lutodrilus multivesiculatus* Lutodrilidae	USA, Louisiana, Washington Parish (coll S. James, M. Callaham, M. Damoff, C. Erseus, 17 Jan 2011)	NCSM 00000	57341	1049
*Maoridrilus wilkini* Acanthodrilidae	New Zealand, Kelly’s Creek (coll. T. Buckley)	NCSM 27279	80910	861
Microchaetidae sp.	South Africa, Western Cape, Tokai Swamp (colls. S. James and D. Plisko, 29 Aug 2011)	NCSM 27280	194638	1053
*Microchaetus* sp. Microchaetidae	South Africa, Northern Cape, Niewwoudtville (colls. S. James, D. Plisko 5 Sep 2011)	NCSM 27281	125494	1093
*Parachilota* sp. Acanthodrilidae	South Africa, Western Cape, Table Mountain (coll. James, Meassey, Plisko, 26 Aug 2011)	NCSM 27282	102971	1074
Place Kabary 2 sp. Acanthodrilidae	Madagascar, Place Kabary, Antsiranana, 12°16'58.27''S 49°17'25.94''E	NCSM 00000	146018	1157
*Pontodrilus litoralis* Megascolecidae	USA, Cedar Point, Alabama (colls. S. James, C. Erséus 17 January 2011)	NCSM 00000	90268	1189
*Rhinodrilus priollii* Rhinodrilidae	Brazil, Amazonas, Reserve Ducke (colls. S. James, S. Coral, 3 Feb 2012)	NCSM 00000	87158	1102
*Scherotheca savignyi* Lumbricidae	France, Midi-Pyrénées, Ariège, Malegoude (coll. S. James, 2 Mar 2011)	NCSM 27283	113157	1041
*Sparganophilus* sp. Sparganophilidae	USA, Iowa, Des Moines River, at Douds (coll. S. James 12 May 2012)	NCSM 27284	123905	1199
*Urobenus brasiliensis* Rhinodrilidae	Brazil, Rio Grande do Sul, Santo Cristo (coll. G. Steffen 09 Sep 2009)	NCSM 27285	55709	890
*Vignysa popi* Hormogastridae	France, Aveyron, Montpellier (colls. S. James, M. Bouche, 1 Mar 2011)	NCSM 27286	93260	779
Outgroups				
*Delaya leruthi* Haplotaxidae	France, Midi-Pyrénées, Ariège, Cazavet, L'Estelas Cave, in water, 43.000 N, 1.010 E (coll. M.C. des Chatelliers, P. Martin & N. Giani, 24 May 2011) (topotype)	SMNH 161293 in EtOH CE13924	118020	1067
*Pelodrilus* sp. Haplotaxidae	Western Australia, 25.5 km S of Busselton, Rapids Conservation Park, Margaret River (coll. C. Erséus, 16 Sep 2012)	WAM V9004	100864	1129
*Haplotaxis gordioides* Haplotaxidae	Sweden, Västergötland, Göteborg, seeping groundwater at Göteborg Botanical Garden (Vitsippsdalen), 57.6813 N, 11.9562 E (C. Erséus & A. Achurra, 29 Mar 2011)	SMNH 161294 in EtOH CE11200	53878	855
*?*Haplotaxidae sp.	Brazil, Amazonas, Reserva Ducke (colls. S. James, S. Coral, 3 Feb 2012) (topotype)	NCSM 000000 in EtOH CE14372	93548	1053
*Lumbriculus variegatus* Lumbriculidae	Sweden, Västergötland, Göteborg, Guldheden, spring S of Dr Fries Torg, 57.6827 N, 11.9707 E (coll. M. Svensson, 8 Nov 2011)	SMNH 161295 slide CE13679	109949	985
*Propappus volki* Propappidae	Sweden, Blekinge, Ronneby, Väby, Bräkneån River, sand in rapids, 56.1792 N, 15.1052 E (C. Erséus, B. Williams & S. Martinsson, 31 May 2013) (topotype)	SMNH 161296 slide CE18375	131574	1140

^a^ numbers of contigs and orthologs pooled across transcriptomes from three tissue types; see [84] for details

*MNHN* National Museum of Natural History (Paris, France), *NCSM* North Carolina Museum of Natural Sciences, *SMNH* Swedish Museum of Natural History, *WAM* Western Australian Museum; some specimens include preservation type and co-author Erséus’s specimen ID numbers (CE#####)

**Fig. 1:**
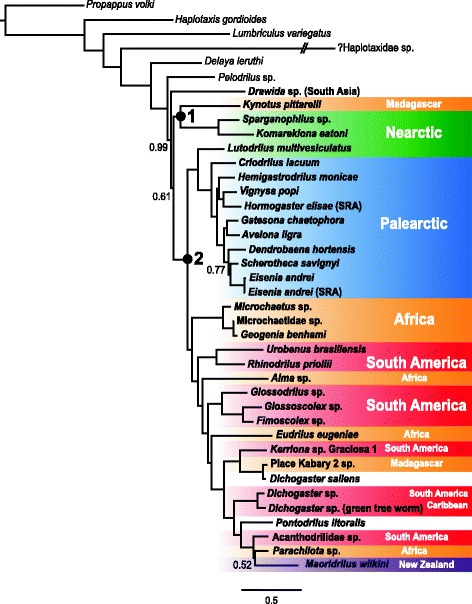
PhyloBayes 50%-majority-rule consensus phylogram for the 75% data set (59 loci, 16,458 amino acid characters, CAT-GTR model, 500-generation burn-in; see text for details). Posterior probabilities are shown at nodes; nodes without values have posterior probabilities of 1.0. Members of Metagynophora (Moniligastridae + Crassiclitellata) are highlighted in bold font; with *Drawida* sp. representing Moniligastridae. Transcriptomes downloaded from the Sequence Read Archive are labeled “(SRA)”. Crassiclitellate taxa are color coded by biogeographic region; *Dichogaster saliens* and *Pontodrilus litoralis* are cosmopolitan species. Dates for nodes labeled 1 and 2 were estimated with PhyloBayes; see text for details

The crassiclitellate samples represented all extant crassiclitellate families but one (Biwadrilidae) and at least 28 genera. Transcriptomes for thirty-one crassiclitellate taxa and all six outgroup taxa were generated as part of this study, and two additional crassiclitellate transcriptomes were assembled as described below from data in the Sequence Read Archive (SRA; http://www.ncbi.nlm.nih.gov/sra/) for *Hormogaster elisae* (PRJNA196484) and *Eisenia andrei* (DRX021555). A transcriptome was also generated for a representative of Moniligastridae (*Drawida* sp.). Voucher specimens are deposited at the North Carolina Museum of Natural Sciences (NCSM), the Swedish Museum of Natural History (SMNH) and the Western Australian Museum (WAM) (Table 1).

### Molecular techniques

Total RNA was extracted from RNA*later*®-preserved samples using the Ambion RNAqueous®-Micro Total RNA Isolation kit. First-strand cDNA was constructed using the SMART® cDNA Library Construction Kit (Clontech Laboratories, Inc.), replacing the included 3′ primer with the Cap-TRSA-CV oligo [19]. We amplified double-stranded cDNA using the Advantage® 2 PCR Kit (Clontech Laboratories, Inc.). To minimize the risk of contamination, extractions and cDNA construction were performed in small batches of four tissue samples or fewer, and the workstation and tools were cleaned with bleach between each set of extractions. Where possible, we avoided sampling the external body surface and the gut to limit the potential for contamination from epibionts and gut contents (e.g., prey items and microorganisms).

Non-normalized cDNA libraries were sent to Hudson Alpha Institute for Biotechnology, Huntsville, Alabama USA for library preparation and 2 × 100–bp paired-end sequencing on an Illumina HiSeq 2000. Approximately one-sixth of a lane was used for each taxon.

### Sequence assembly and processing

Raw PE Illumina reads were digitally normalized using khmer (normalize-by-median.py -C 30 -k 20 -N 4 -× 2.5e9) [20] and assembled using the October 5, 2012 release of Trinity [21]. We used TransDecoder (http://transdecoder.github.io) to find open reading frames and translate nucleotide sequences into amino acid sequences that were at least 100 amino acids in length.

### Dataset construction

Translated data for all 40 taxa were searched against the Lophotrochozoa pHMMs in HaMStR v.13.2.3 [22] using *Helobdella robusta* as reference species. We set HaMStR to output all sequences that fulfilled the reciprocity requirement and then used a custom script to generate FASTA-formatted files for each orthogroup that included all sequences and deleted duplicated contigs. Each orthogroup was then aligned with MAFFT (L-INS-i) [23].

One of the major difficulties in phylogenomic analysis—particularly when dealing with transcriptome data—is orthology assessment. Most animals harbor paralogous copies of many genes, but standard molecular phylogenetic analyses assume that data sampled from each taxon for each locus are orthologs. Failure to distinguish orthologs from paralogs can cause major problems in phylogenetic inference [24]. Given this, we used a tree-based approach to remove likely paralogs from our alignments. We inferred a maximum-likelihood (ML) tree for each aligned orthogroup with FastTreeMP [25] (under the –slow and –gamma settings), and used PhyloTreePruner [26] to screen each of the resulting trees. In PhyloTreePruner, nodes on each ML tree with SH-like local support values <0.7 were collapsed into polytomies, and the largest subtree was retained where each taxon was represented by either no sequences or only one sequence, unless all sequences for a given taxon formed part of a clade or part of the same polytomy (in which case, all were retained). Sequences falling outside this maximally inclusive subtree were assumed to be paralogs and were deleted from the data set. If multiple in-paralogs were initially retained, all but the longest sequence were subsequently deleted by PhyloTreePruner. This returned an alignment for each orthogroup that included (at most) a single, putatively orthologous sequence for each taxon. PhyloTreePruner was used to retain only orthogroups found in at least 25% (10 taxa), 50% (20 taxa), 75% (30 taxa) and 100% (40 taxa) of transcriptomes. All loci were subsequently realigned with MAFFT (L-INS-i). FASconCAT v1.0.pl [27] was then used to concatenate orthogroups. The ProteinModelSelection.pl script (https://github.com/stamatak/standard-RAxML/blob/master/usefulScripts/ProteinModelSelection.pl) was used to find the best-fitting amino-acid substitution model for each orthogroup (for downstream analyses using TreSpEx; see below) and for each concatenated data matrix. We chose not to use any automated alignment filtering methods (e.g., GBlocks [28]), due to concerns about their efficacy in improving phylogenetic inference [29].

Distantly related outgroups may be problematic for phylogenomic inference [30]. We used two approaches to explore the effect of outgroup sampling on estimates of ingroup relationships. First, we deleted *Lumbriculus variegatus* and *Propappus volki* (the two most distant outgroups in terms of summed branch length to the base of Crassiclitellata across analyses) and “?Haplotaxidae sp.” (a conspicuously long outgroup branch) from the set of transcriptomes prior to processing with the approach outlined above, leaving a total of 37 taxa. Following the approach outlined above, we used PhyloTreePruner to only retain orthogroups found in at least 25, 50 and 75% of the taxa (in this case, 10, 19 and 28 taxa, respectively). Second, we deleted only “?Haplotaxidae sp.” from the original set of transcriptomes, leaving a total of 39 taxa. For this data set, we processed the transcriptomes as described above, but used PhyloTreePruner to only retain orthogroups found in ≥75% of the taxa (i.e., 30 taxa). To assess the influence of sites with high percentages of gaps/missing data on our inferences, we produced two concatenated “no ?Haplotaxidae sp.” 75% data matrices. For one, we did no additional filtering. For the other, we used TrimAl v1.2 [31] to remove all sites comprising >50% gaps from each individual orthogroup alignment prior to concatenation and model testing. Amounts of missing data per taxon were calculated using TREE-PUZZLE 4.3 [32] for all matrices.

All data matrices, ML tree files, custom scripts and supplementary figures are available via the Dryad Digital Repository (http://datadryad.org/resource/doi:10.5061/dryad.n7n71).

### Long-branch effects and compositional heterogeneity

Differences in substitution rates and nucleotide/amino acid composition among lineages constitute two well-known confounding factors in phylogenetic analysis [33–36]. To assess potential impact of these factors on our inferences, TreSpEx.v1.1 [37] was used to calculate three measures of branch-length heterogeneity—the average patristic distance (PD), the standard deviation of the tip-to-root distance and the LB score (the mean pairwise PD of a taxon to all other taxa in the tree relative to the average pairwise PD over all taxa [37])—for each locus. Any single-gene alignment that had a value equal to or greater than 1.5 times the interquartile range above the median for any of these three indices was eliminated. Remaining loci were evaluated with BaCoCa v. 1.104r [38]. Data partitions (loci) with a *p*-value of less than 0.05 for a chi-square test of homogeneity were eliminated, as were all loci that were 1.5 times the interquartile range above the median RCFV value. RCFV measures the absolute deviation from the mean for each amino acid and taxon, in this case summed across taxa for each partition (locus); higher RCFVs indicate a higher degree of compositional heterogeneity in that partition [39]. TreSpEx and BaCoCa filtering was not applied to the 100% data set, which was already quite small in terms of number of loci (Table 2).

**Table 2:** Characteristics of all data matrices analyzed in this study

Data Set	# Loci	# Characters	# Parsimony-informative Characters	% Missing
25%				
Unpartitioned/partitioned original data	766	361,365	64,892	77.27
TreSpEx + BaCoCa filtered	543	251,614	40,509	77.56
Deleted outgroups	727	337,650	59,520	76.97
50%				
Unpartitioned/partitioned original data	162	58,085	18,515	55.57
TreSpEx + BaCoCa filtered	131	46,468	15,173	53.29
Deleted outgroups	206	78,060	22,753	55.34
75%				
Unpartitioned/partitioned original data	58	16,458	6596	35.21
TreSpEx + BaCoCa filtered	49	14,075	5785	34.99
Deleted outgroups	92	28,097	9844	35.00
No ?Haplotaxidae sp., all sites	56	16,541	6879	33.45
No ?Haplotaxidae sp., no gappy sites	56	13,168	6443	18.62
100%				
Unpartitioned/partitioned original data	7	1997	805	20.21

### Maximum Likelihood (ML) analyses

Partitioned maximum-likelihood (ML) analyses were conducted with RAxML versions 8.1.24 and 8.2.3 [40] on CIPRES [41] with 1000 rapid bootstrap replicates, using the following options: -f a -x < random number seed for rapid bootstrapping; unique for each analysis > −p < random number seed for initial parsimony inferences; unique for each analysis > −# 1000 -m PROTGAMMA < amino acid model > −s < inputfile> − n < outputfile> (Table 2). Best-fitting amino acid substitution models were inferred for each locus and applied to each locus in RAxML by adding “-q < partitionfile>” to the command listed above. Identical random number seeds for rapid bootstrapping and parsimony inferences were used for the two “no ?Haplotaxidae sp.” 75% matrices (one that was not cleaned with TrimAl and one from which sites with >50% gaps were removed, both filtered with TreSpEx and BaCoCa) to allow a direct comparison of tree topologies for these two matrices.

We used SuperQ v.1.1 [42] to visualize topological conflict among loci for the 25, 50 and 75% unfiltered data sets. SuperQ rescales the partial, unrooted ML gene trees for each data set to produce comparable branch lengths, decomposes the trees into weighted quartet trees and employs the QNet algorithm to produce a split network from the quartet trees. We used the Gurobi optimizer to calculate initial split weights and optimize the weights under the “balanced” objective function. We used SplitsTree v.4.14.4 [43] to visualize the resulting networks.

### Bayesian Inference (BI) analyses

Site-heterogeneous Bayesian Inference (BI) analyses of the 25, 50, and 75% data sets and for the two filtered “no ?Haplotaxidae sp.” 75% matrices (one that was not cleaned with TrimAl and one from which sites with >50% gaps were removed) were conducted with PhyloBayes-MPI v1.5a [44] under the CAT-GTR model with two independent chains and gamma-distributed rates on CIPRES. Analyses were allowed to run for up to 168 h (the CIPRES limit), constant sites were removed, and four categories were used for the discrete gamma distribution. Convergence checks were conducted automatically every 1800 s and analyses were terminated early if after a burn-in of 500 cycles, the minimum effective size exceeded 50, and the “maxdiff” value between chains was less than 0.1. For runs that terminated due to reaching the time limit, convergence of parameter estimates and topologies across chains was assessed by evaluating the basecomp and tracecomp files produced by PhyloBayes and via visual inspection of trace files in Tracer v1.6 [45].

### Topology tests

Tree topologies recovered in our analyses contradicted previous hypotheses regarding the monophyly of *Dichogaster* (see below). The Shimodaira-Hasegawa and approximately unbiased tests [46, 47] are often used to evaluate particular topological hypotheses (including at least one hypothesis chosen a posteriori), but these tests are actually designed to evaluate whether *all* topologies in a plausible set of topologies are equally good explanations of the data, rather than to compare specific alternative topologies [48]. Fortunately, the parametric bootstrapping (SOWH) test [48, 49] and Bayesian topology tests [50] are both appropriate in this context.

We used SOWHAT [51] to perform SOWH tests to test *Dichogaster* monophyly. SOWH tests require two ML analyses—an unconstrained analysis and an analysis in which the topology is constrained to match a particular alternative hypothesis. The difference in likelihoods between the trees resulting from each analysis (δ) constitutes the test statistic for the SOWH test. The ML topology and branch lengths from the constrained analysis are then used to simulate a large number of data sets using the model parameter estimates for the constrained ML topology and original data. We provided SOWHAT with a *Dichogaster* monophyly constraint (forcing monophyly of the three *Dichogaster* transcriptomes) in Newick format and a reduced data set in which three distant/long-branch outgroup taxa (*Propappus volki*, *Lumbriculus variegatus* and ?Haplotaxidae sp.) were removed, retaining only orthogroups found in at least 28 of the transcriptomes, emulating the 75% data set described above. SOWHAT called Seq-Gen 1.3.2 [52] to simulate 100 data sets and RAxML 8.2.8 [40] to infer topologies for each simulated data set in an unconstrained and constrained ML analysis. SOWHAT calculates confidence intervals around a SOWH test *p*-value after addition of each replicate to determine if the sample size of the test was adequate.

For Bayesian topology tests, we used the posterior sample of trees generated in the PhyloBayes CAT-GTR analysis of the 75% data set to estimate posterior model odds for alternative topological hypotheses, following suggestions by Bergsten et al. [50]. We calculated posterior model odds by dividing the frequency of trees in the post burn-in sample of trees that support one hypothesis (e.g., *Dichogaster* is not monophyletic) by the frequency of trees that support the alternative hypothesis (e.g., *Dichogaster* is monophyletic; all three *Dichogaster* transcriptomes form a clade).

### Divergence time estimation

Unfortunately, the dearth of fossils that can be attributed to earthworms [53, 54] presents a challenge for estimating divergence times, but there are some relevant fossils as well as some previous dating studies on earthworms. Putative earthworm trace fossils (burrows or casts) have been recovered from the Triassic [55], with possible body fossils in the Paleocene [56]. Possible clitellate body fossils have been recovered from Permian deposits [57], and fossil leech cocoons are known from the late Triassic [58]. Finally, a molecular study of hormogastrid earthworms (calibrated using the separation of the Corso-Sardinian microplate from continental Europe) suggests that they radiated in the Late Cretaceous [59]; if this is correct, the common ancestor of all crassiclitellates must have arisen much earlier.

These fossils and inferences give us a set of calibration points that we can use to estimate dates for key divergences within our phylogenies. We performed dating analyses for three data matrices: the unfiltered 75% data set (including ?Haplotaxidae sp.) and two versions of the 75% data matrix that did not include ?Haplotaxidae sp. (one with all sites and the other with sites containing >50% gaps removed, both filtered with TreSpEx and BaCoCa as described above) in PhyloBayes 3.3f [60]. In each case, we used the CAT-GTR PhyloBayes majority rule consensus tree for each data matrix as a fixed topology. We ran four independent chains for each data set, sampling every ten cycles, under the CAT-GTR substitution model with gamma-distributed rates, a lognormal autocorrelated relaxed clock model and a uniform prior on divergence times.

We used three calibration points/ranges in our analyses—the oldest known leech cocoon fossil (201 Mya) [58], the divergence of Hormogastridae (67–97 Mya) [59] and a minimum age estimate for crown-group Annelida of 520 Mya (based on the earliest known—probably stem-group—polychaetes from the Sirius Passet deposit of North Greenland; [61–64]). Though we did not include leeches in our analyses, previous studies have supported a sister-group relationship between leeches and their allies (branchiobdellidans and *Acanthobdella*) and Lumbriculidae [13, 18], providing a minimum age for divergence of the Lumbiculidae + Hirudinea clade and Crassiclitellata based on the earliest fossil cocoons attributable to leeches. We used 67 Mya as a minimum age and 97 Mya as a maximum age for the deepest divergence within Hormogastridae as represented in our data matrices [59] (the node subtending *Hemigastrodrilus monicae* and *Vignysa popi*/*Hormogaster elisae*; a recent phylogenomic study of Hormogastridae [65] corroborates this pattern of relationships). Finally, we argue that a minimum age of crown-group Annelida (520 Mya) is suitable as a maximum age constraint for the root of our phylogeny, because no evidence of clitellates is known prior to the Permian, and the root of our phylogeny is deeply nested within Clitellata, which is itself deeply nested within the annelid crown group.

The calibration for the divergence between Lumbriculidae and Hirudinea (201 mya) was treated as a hard upper bound, with the lower bound modeled as a truncated Cauchy distribution (*p* = 0.1 and c = 1). We placed uniform priors of 67–97 mya and 201–520 mya on the Hormogastridae divergence and the root node, respectively. Convergence was assessed with estimated sample sizes and visual inspection of parameter traces in Tracer v1.6. To assess whether the priors conditional on our calibrations match our intended prior distributions, we ran PhyloBayes under the prior and our calibrations using the F81 model without rate variation across sites (these model parameters do not factor into the prior over divergence times) and visually inspected the results.

We focused on divergence times for two nodes in our phylogeny that separated Northern and Southern Hemisphere subclades—1) a node separating *Kynotus pittarelli* (Madagascar) and a clade comprising *Sparganophilus* sp. and *Komarekiona eatoni* (both found in eastern North America) and 2) a node separating a Northern Hemisphere clade comprising *Lutodrilus* (North America) and Lumbricoidea (Criodrilidae, Hormogastridae, Lumbricidae) (Europa and Asia) and a primarily Southern Hemisphere clade comprising representatives of Almidae, Acanthodrilidae, Eudrilidae, Glossoscolecidae, Megascolecidae, Microchaetidae and Ocnerodrilidae (Africa, Australia, New Zealand and South America). We hypothesized that these divergences may be due to vicariance during the breakup of Pangaea starting in the late Triassic to early Jurassic (~200–185 Mya) [66, 67]; divergence time estimation using molecular data allows a test of this hypothesis.

Ideally, we would also infer dates using a Bayesian method such as BEAST [68], but preliminary analyses suggested that the computational demands of inferring divergence times for our data in this manner would be prohibitive.

## Results

Transcriptomes for thirty-one crassiclitellates, one moniligastrid, and six outgroup taxa were generated as part of this study (Table 1; Additional file 1: Figure S1) and are available from the SRA at NCBI under BioProject accession number PRJNA362879. We added publicly available transcriptome data for two additional crassiclitellates—*Hormogaster elisae* (PRJNA196484) and *Eisenia andrei* (PRJDB3115)—for a total of forty transcriptomes. Four gene sets from these transcriptomes were analyzed, reflecting different levels of gene occupancy.

### Ingroup relationships

We filtered our data matrices to attempt to account for several issues known to cause problems in phylogenomic analysis. First, we built four data sets representing different levels of missing data, ranging from a data matrix with a high number of genes but also a high amount of missing data (i.e., the 25% data set) to a data matrix with a very low number of genes and very little missing data (i.e., the 100% data set) (Table 2). Second, we attempted to improve substitution model fit by partitioning our matrices by orthogroup (gene) and inferring best-fitting substitution models for each gene and also by using a site-heterogeneous model (CAT-GTR) in PhyloBayes. Third, we eliminated subsets of loci that showed high levels of branch-length heterogeneity (which could be due to either the presence of previously undetected paralogs or substitutional rate differences among taxa) and amino-acid compositional heterogeneity with TreSpEx and BaCoCa; trees for one set of genes that passed through this filter generally show low levels of branch-length heterogeneity (Additional file 2: Figure S2). Amounts of missing data per taxon varied widely among taxa and across matrices, ranging from a low value of 1.57% (*Hormogaster elisae*, 75% no ?Haplotaxidae sp., gappy sites removed matrix) to a high value of 93.32% (Alma sp., 25% unfiltered matrix) (Table 3). *Alma* sp. had the most missing data, followed by *Fimoscolex* sp. and *Glossoscolex* sp. (Table 3; Additional file 1: Figure S1).

**Table 3:** Percent missing data per taxon across all matrices analyzed in this study, calculated using TREE-PUZZLE 5.3

	Matrix											
Taxon	25 U	25 T	25D	50 U	50 T	50D	75 U	75 T	75D	75H	75HG	100 U
Acanthodrilidae sp.	71.28	70.48	71.28	49.23	48.86	49.23	24.94	48.86	24.94	25.32	8.29	16.62
*Alma* sp.	93.52	93.39	93.52	84.99	84.61	84.99	71.86	84.61	71.86	73.99	68.13	44.37
*Avelona ligra*	70.46	67.95	70.46	47.68	42.17	47.68	26.91	42.17	26.91	23.52	6.48	14.97
*Criodrilus lacuum*	84.72	84.45	84.72	62.46	57.70	62.46	44.00	57.70	44.00	44.02	31.04	20.93
*Delaya leruthi*	78.74	82.55	78.74	52.10	50.12	52.10	28.27	50.12	28.27	29.27	14.19	16.88
*Dendrobaena hortensis*	70.35	69.16	70.35	44.63	39.30	44.63	23.86	39.30	23.86	18.36	2.59	12.52
*Dichogaster* sp. (green tree worm)	71.97	70.21	71.97	54.66	53.56	54.66	29.06	53.56	29.06	24.36	7.03	17.93
*Dichogaster* sp.	69.76	67.77	69.76	50.28	49.08	50.28	27.37	49.08	27.37	24.48	7.49	16.07
*Dichogaster saliens*	80.45	78.88	80.45	63.13	62.73	63.13	34.41	62.73	34.41	27.56	11.44	17.63
*Drawida* sp.	78.41	80.37	78.41	57.60	55.64	57.60	39.68	55.64	39.68	39.08	25.80	15.82
*Eisenia andrei*	62.78	62.04	62.78	39.83	37.24	39.83	25.17	37.24	25.17	23.14	5.07	16.07
*Eisenia andrei* SRA	69.12	66.93	69.12	43.18	38.61	43.18	25.96	38.61	25.96	21.76	4.33	19.93
*Eudrilus eugeniae*	79.96	80.02	79.96	58.95	57.36	58.95	31.73	57.36	31.73	31.96	15.98	23.99
*Fimoscolex* sp.	88.37	87.75	88.37	76.41	75.80	76.41	56.96	75.80	56.96	61.63	52.16	31.90
*Gatesona chaetophora*	79.32	78.45	79.32	54.76	49.42	54.76	27.69	49.42	27.69	23.66	7.19	15.42
*Geogenia benhami*	83.35	84.86	83.35	62.53	57.10	62.53	38.68	57.10	38.68	41.06	27.62	19.83
*Glossodrilus* sp.	76.14	75.65	76.14	54.69	53.03	54.69	33.19	53.03	33.19	31.04	14.94	18.13
*Glossoscolex* sp.	88.17	87.55	88.17	73.82	73.67	73.82	65.22	73.67	65.22	59.75	49.91	33.55
*Haplotaxis gordioides*	86.63	89.16	86.63	69.18	69.65	69.18	45.94	69.65	45.94	42.19	30.48	21.58
?Haplotaxidae sp.	81.18	86.38	-----	55.95	54.58	-----	31.37	54.58	-----	22.63	-----	-----
*Hemigastrodrilus monicae*	74.66	73.40	74.66	47.84	42.08	47.84	28.08	42.08	28.08	22.91	6.14	14.72
*Hormogaster elisae*	61.55	61.14	61.55	40.13	35.46	40.13	22.07	35.46	22.07	18.66	1.57	15.17
*Kerriona* sp. Graciosa1	79.62	77.63	79.62	68.83	69.86	68.83	46.30	69.86	46.30	44.66	32.04	16.78
*Komarekiona eatoni*	75.54	78.33	75.54	49.28	46.41	49.28	30.93	46.41	30.93	29.50	14.06	19.33
*Kynotus pittarelli*	78.34	80.98	78.34	48.40	44.51	48.40	31.88	44.51	31.88	33.44	19.32	20.93
*Lumbriculus variegatus*	84.69	86.95	-----	62.91	63.29	-----	44.54	63.29	-----	45.40	34.07	33.20
*Lutodrilus multivesiculatus*	78.23	77.77	78.23	50.82	47.41	50.82	27.34	47.41	27.34	23.54	6.18	18.38
*Maoridrilus wilkini*	86.86	86.10	86.86	75.28	75.10	75.28	60.26	75.10	60.26	54.11	43.85	35.90
Microchaetidae sp.	79.37	80.11	79.37	48.98	43.78	48.98	28.13	43.78	28.13	24.11	7.76	15.32
*Microchaetus* sp.	74.62	75.20	74.62	45.96	42.02	45.96	27.69	42.02	27.69	26.44	9.31	14.77
*Parachilota* sp.	73.61	72.09	73.61	59.08	58.99	59.08	42.15	58.99	42.15	32.77	17.19	25.94
*Pelodrilus* sp.	75.31	79.76	75.31	50.98	50.45	50.98	32.80	50.45	32.80	35.99	21.16	16.93
Place Kabary 2 sp.	70.17	68.40	70.17	49.41	49.91	49.41	23.89	49.91	23.89	23.47	6.43	18.58
*Pontodrilus litoralis*	66.37	66.34	66.37	46.87	46.53	46.87	26.84	46.53	26.84	19.66	2.16	15.92
*Propappus volki*	77.03	81.59	-----	56.39	57.47	-----	36.90	57.47	-----	36.68	22.49	15.02
*Rhinodrilus priollii*	75.00	76.10	75.00	45.59	43.06	45.59	24.06	43.06	24.06	26.27	9.64	16.17
*Scherotheca savignyi*	76.55	75.20	76.55	54.39	49.26	54.39	30.39	49.26	30.39	28.99	12.94	14.67
*Sparganophilus* sp.	68.43	71.47	68.43	36.25	32.65	36.25	21.49	32.65	21.49	23.05	7.09	13.37
*Urobenus brasiliensis*	84.07	85.13	84.07	59.66	56.92	59.66	39.04	56.92	39.04	37.79	23.41	24.69
*Vignysa popi*	86.01	84.87	86.01	69.72	66.08	69.72	50.88	66.08	50.88	51.03	39.39	21.53

Matrices are coded as percentages (25%, 50% or 75%); *U* unfiltered, *T* TreSpEx and BaCoCa filtered, *D* “deleted outgroups”, *H* “no ?Haplotaxidae sp.”, all sites included, *HG* “no ?Haplotaxidae sp.”, no gappy sites

**Fig. 2:**
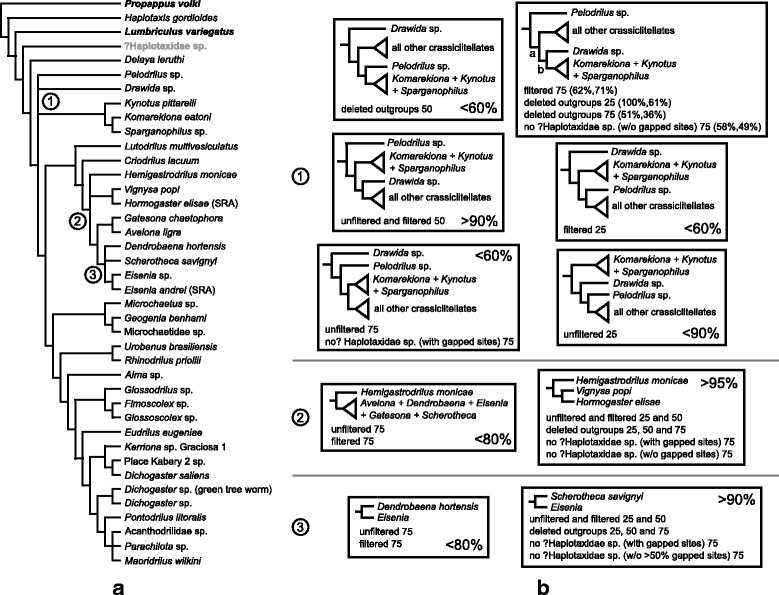
**a** Strict consensus tree of ML trees for unfiltered, filtered (with TreSpEx and BaCoCa) and deleted outgroup 75, 50 and 25% data matrices and two 75% data matrices from which ?Haplotaxidae sp. was deleted (one with all sites included, the other with sites with >50% gaps deleted) (eleven analyses/trees total). Numbers at nodes highlight well-supported discrepancies across analyses. Taxa in bold black or gray were not included in the “deleted outgroup” analyses. **b** Resolutions of conflicting clades across eleven analysis/matrix combinations, with ML bootstrap values or ranges shown

Across this array of data matrices and analyses, inferred patterns of relationships within Crassiclitellata were largely congruent and well supported (Figs. 1 and 2). Bayesian analyses under the CAT-GTR model were attempted for the unfiltered 25, 50, and 75% data sets and the two filtered “no ?Haplotaxidae sp.” 75% matrices in PhyloBayes, but examination of PhyloBayes output for the 25% data set in Tracer confirmed that it did not converge. For the 75% data set, two chains ran for an average of 17,890 cycles; for the 50% data set, two chains ran for an average of 8915 cycles. The filtered “no ?Haplotaxidae sp.” 75% matrix with all sites ran for an average of 18,465 cycles; the filtered “no ?Haplotaxidae sp.” 75% matrix with >50% gap sites deleted ran for an average of 29,205 cycles (Additional file 3: Figure S3). The largest discrepancy observed across all bipartitions was <0.1, the maximum discrepancy between the chains was ~0.3 and the effective sample sizes for all parameters were >50, all suggesting an acceptable PhyloBayes run for the 75% data set (the same was true for the analysis of the 50% data set, except that the maximum discrepancy was <0.5). The 75 and 50% PhyloBayes majority-rule consensus topologies are almost identical except for the position of *Drawida* sp. (recovered as sister to the clade comprising all crassiclitellates except *Kynotus*, *Komarekiona* and *Sparganophilus* in the 50% tree), so only the 75% PhyloBayes tree is shown (Fig. 1).

Within Crassiclitellata, several clades were consistently recovered, including a clade comprising Kynotidae (Madagascar), Sparganophilidae and Komarekionidae (both found in the southern and eastern United States) as sister to the rest of Crassiclitellata. All analyses revealed a deep split between two major clades, one with a largely Northern Hemisphere (Laurasian) distribution (Lumbricoidea sensu James and Davidson [12]; Lumbricidae, Hormogastridae, Criodrilidae and *Lutodrilus multivesiculatus*) and the other with a primarily Southern Hemisphere (Gondwanan) distribution (Microchaetidae, Rhinodrilidae, Almidae, Glossoscolecidae, Eudrilidae and Megascolecoidea sensu James and Davidson [12]) (note that Rhinodrilidae was mistakenly given the name Pontoscolecidae in James and Davidson [13]; this error was subsequently corrected [69].

Data partitioning, deleting long-branch outgroup taxa (e.g., ?Haplotaxidae sp.) and removal of loci that showed variable rates of change in different lineages or signs of compositional heterogeneity had little impact on trees resulting from analyses of the 25, 50, and 75% data sets. Relationships recovered in analyses of the 100% data set were poorly supported, likely reflecting the very small size of this data set (7 loci, <2000 amino acids; Table 2), and will not be discussed in detail. Across the 25, 50, and 75% data sets, relationships differed in only two ingroup clades. In one of these cases (relationships among *Maoridrilus*, *Parachilota* and Acanthodrilidae sp.), bootstrap support for any particular resolution was low across all analyses (Fig. 2). In the other, support for Hormogastridae (represented here by *Hemigastrodrilus*, *Vignysa* and *Hormogaster*) and a *Scherotheca* + *Eisenia* pairing increased as the number of genes (and amount of missing data) increased (Fig. 2). Bootstrap support for both of these clades was >90% across all data sets from which one or more distant/long-branch outgroup taxa were deleted. With the exception of these cases, all ingroup relationships were identical across all trees, whether or not ?Haplotaxidae sp. was included, sites containing >50% gaps were deleted, the data were filtered with TreSpEx and BaCoCa or analyzed with a site-heterogeneous model in PhyloBayes.

**Fig. 3:**
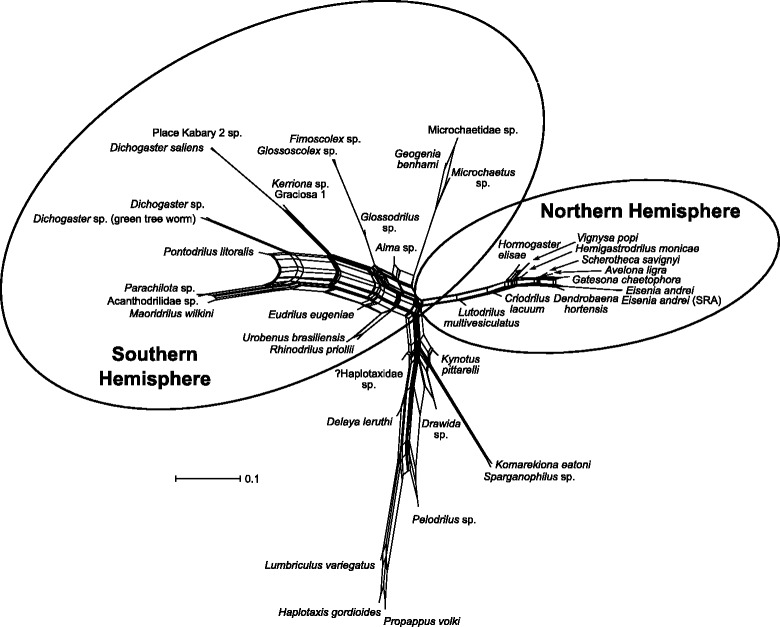
Quartet-based supernetwork based on all single-gene ML trees for the 75% data set (49 genes). Geographically delineated subclades of the main crassiclitellate clade (the clade subtending node 2 in Fig. 1) are *circled*

The split networks produced with SplitsTree from the SuperQ lists of weighted splits generally reflect the tree-like structure recovered in concatenated ML and Bayesian analyses, but also show substantial incongruence among loci in all three unfiltered data sets (Fig. 3; the three supernetworks are very similar, so only the supernetwork for the 75% data set is shown).

### Outgroups and basal relationships

Despite the high stability and levels of bootstrap support for relationships within the ingroup across analyses, positions of some outgroup taxa and the lone representative of Moniligastridae (*Drawida*) varied among data sets and analyses (Fig. 2). Based on previous analyses [13], we expected *Propappus volki* to be a suitable distant outgroup to root our phylogeny, with Lumbriculidae and Haplotaxidae forming successively closer outgroups to Crassiclitellata. However, most analyses failed to recover this pattern of relationships. Partitioned ML analyses of the 25% through 75% data matrices supported a paraphyletic Crassiclitellata and Metagynophora (due to the inclusion of *Pelodrilus* sp., an alleged haplotaxid) as well as a doubly paraphyletic Haplotaxidae (due to the inclusion of both Metagynophora and *Lumbriculus variegatus*) (Fig. 2). Recovery of topologies in which *Lumbriculus variegatus* is more closely related to Metagynophora than *Haplotaxis gordioides* is strains credulity; *Lumbriculus* is a member of Lumbriculidae, a clitellate group that previous molecular and morphological phylogenetic reconstructions (18S data; [13, 18]) suggest is more closely related to leeches (Hirudinida) than to haplotaxids or crassiclitellates.

Our outgroup sampling was designed to test crassiclitellate monophyly and root Crassiclitellata, not to infer deep-level relationships among major clitellate taxa. As such, unexpected relationships among outgroups may not be surprising, but failure to recover Crassiclitellata is of greater concern—in some trees (e.g., based on partitioned ML analyses of the 25 and 50% data sets), *Drawida* (Moniligastridae) was found to be nested within Crassiclitellata, usually as sister to a clade comprising all earthworms except *Komarekiona*, *Kynotus* and *Sparganophilus* (Fig. 2). Removal of outlier loci detected by TreSpEx and BaCoCa did not consistently recover expected relationships among the outgroup taxa, nor did it consistently yield a monophyletic Crassiclitellata across data sets (Fig. 2b).

Elimination of potentially problematic loci is one way to explore the impact of systematic bias and possibly improve inferences; elimination of potentially problematic *taxa* is another. Inclusion of distant outgroups can perturb phylogenomic analyses, particularly with respect to basal ingroup relationships [30]. Cursory visual inspection of our trees revealed that one of the haplotaxids in our data sets—?Haplotaxidae sp.—is a rather long-branch taxon, and this could be confounding our results. To test this, we eliminated ?Haplotaxidae sp. alone, or the two (putatively) most distant outgroup taxa in our data sets (*Propappus* and *Lumbriculus*) and ?Haplotaxidae sp. Unfortunately, despite the seemingly positive impact of outgroup deletion on inference of some ingroup relationships (see above), analyses of these matrices failed to clarify basal crassiclitellate relationships, usually yielding trees in which either *Pelodrilus* sp. or *Drawida* sp. was weakly supported as sister to the *Komarekiona* + *Kynotus* + *Sparganophilus* clade (Fig. 2).

By contrast, PhyloBayes analysis of the unfiltered 75% data set recovered both a monophyletic Crassiclitellata and a monophyletic Metagynophora, though the posterior probability of Crassiclitellata was low (0.61) (Fig. 1). Assuming Crassiclitellata and Metagynophora are, indeed, monophyletic, our PhyloBayes results suggest that accounting for site-specific substitution processes, if computationally feasible, rather than simply partitioning by gene, can yield improved inferences.

### Topology tests

In the SOWH test of *Dichogaster* monophyly, the observed δ test statistic was 4148.083, and *Dichogaster* monophyly was rejected (*p*-value <0.01, 95% confidence interval = 0.03621669–0). No trees in the post burn-in sample of 2578 trees from PhyloBayes include a monophyletic *Dichogaster*, making the posterior model odds in favor of a non-monophyletic *Dichogaster* infinite.

### Divergence times

We ran PhyloBayes under the prior for 370,000+ cycles for the unfiltered 75% matrix, and visual inspection of the output suggests that the induced prior distributions for the root and nodes of interest are non-informative. The dating analysis of the 75% data matrix ran for an average (across four independent chains) of 21,500 cycles, the analysis of the “no ?Haplotaxidae sp.” 75% data matrix, filtered with TreSpEx and BaCoCa with no sites deleted, ran for an average of 16,660 cycles, and the analysis for the “no ?Haplotaxidae sp.” 75% data matrix, filtered with TreSpEx and BaCoCa with sites containing >50% gaps deleted, ran for an average of 18,000 cycles. For all three analyses, inspection of the four chains in Tracer suggested that a 10% burn-in was appropriate, and all ESS values were above 200. The consensus tree topologies differ slightly across the three analyses, most notably in that *Drawida* sp. is recovered as sister to all crassiclitellates in the unfiltered 75% consensus tree (Fig. 2), but it is recovered as sister to the *Komarekiona* + *Kynotus* + *Sparganophilus* clade in the consensus trees for the 75% “no ?Haplotaxidae sp.” analyses (Additional file 3: Figure S3). Date estimates for each node across all four independent chains were within 2% of each other, so only results from the first chain are reported for each data matrix (Table 4); chronograms are presented in Additional file 4: Figure S4.

**Table 4:** PhyloBayes divergence time estimates (mean ± standard error) in millions of years ago for three data matrices for two key nodes in the earthworm radiation—the node separating *Kynotus* from *Sparganophilus* + *Komarekiona* (node 1, Fig. 1) and the node separating the Northern Hemisphere clade comprising *Lutodrilus* and Lumbricoidea and the clade comprising Southern Hemisphere representatives of several families (node 2, Fig. 1)

Data Matrix	Node 1 (mean ± SE)	Node 2 (mean ± SE)
75% with ?Haplotaxidae sp., unfiltered	164.527 ± 22.2868	161.104 ± 21.0178
no ?Haplotaxidae sp., filtered, all sites	186.1521 ± 23.2339	186.0185 ± 22.18
no ?Haplotaxidae sp., filtered, no >50% gaps	178.1059 ± 21.9198	177.6679 ± 21.1014

“Filtered” = loci showing evidence of high levels of branch-length or compositional heterogeneity deleted with TreSpEx and BaCoCa. See text for details

## Discussion

### Crassiclitellata systematics

**Fig. 4:**
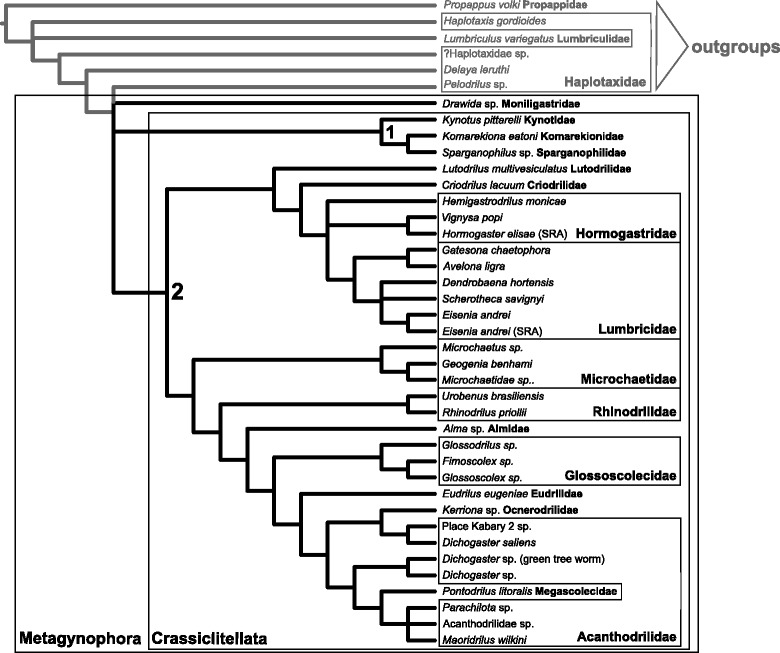
Summary tree of relationships among the taxa included in this study, highlighting the names of relevant higher taxa. The topology shown is a strict consensus of all trees recovered for the 25, 50 and 75% data matrices generated in this study (filtered with TreSpEx and BaCoCa and unfilitered, with and without long-branch outgroups, etc.), with families and major taxa highlighted. The two numbered nodes were the focus of divergence time estimation. See Fig. 2 and text for details

The convergence of results from multiple approaches on a consistent topology (Figs. 1, 2 and 4) provides us with a strong framework for understanding earthworm phylogeny and evolutionary relationships. Our data lend substantial support to revisions of the classical, intuitive, understandings of earthworm phylogeny proposed by James and Davidson [13]. Among the more striking revisions supported here is the placement of Kynotidae (endemic to Madagascar) within a group also containing the exclusively North American families Komarekionidae and Sparganophilidae, rather than sister to or nested within Microchaetidae (South Africa) [70]. Unfortunately, we were unable to obtain suitable material of *Biwadrilus bathybates*, the sole representative of the monotypic family Biwadrilidae from Japan, for transcriptome sequencing; previous work suggests *B. bathybates* may be sister to Kynotidae [13], a relationship that, if supported, reflects a paleogeography no longer obvious from Pangaean or post-Pangaea continental configurations.

A second important revision to classical earthworm thinking supported by the current study is the placement of the monotypic families Lutodrilidae and Criodrilidae as successive sister taxa to the Hormogastridae + Lumbricidae clade. Both Lutodrilidae and Criodrilidae are aquatic, as is Almidae, and these three families show strong morphological similarities of body form (quadrangular tail segments), color (dusky gray with blue-green in the head segments) and clitellum length (extraordinarily long, tens of segments rather than the usual 3 to 10 or so seen in most terrestrial earthworms). The finding that the two closest relatives of the clade comprising the predominant earthworms of Europe (Lumbricidae and Hormogastridae) are aquatic suggests a possible aquatic ancestor for European earthworms. Typically, aquatic earthworms lack dorsal pores, but most members of Lumbricidae have them, as do members of the crown clade Megascolecoidea (represented here by representatives of *Dichogaster*, *Maoridrilus*, *Parachilota* and *Pontodrilus*, along with a thus-far-unidentified acanthodrilid from Madagascar, Place Kabary 2 sp.). Microchaetidae through Eudrilidae and Ocnerodrilidae (represented here by *Kerriona*) lack dorsal pores, with rare exceptions in the last family [71], indicating that dorsal pores probably evolved independently at least twice.

In the current study, placement of the only member of Ocnerodrilidae, *Kerriona* sp. Graciosa 1, as sister to a clade composed of *Dichogaster saliens* (Benhamiidae) and the acanthodriline Place Kabary 2 sp. is unusual and, if validated with a larger sampling of Ocnerodrilidae, would be a major change in the systematics of Megascolecoidea. Traditionally Ocnerodrilidae is considered to be close to acanthodriline earthworms (Acanthodrilidae, Benhamiidae, and “Octochaetidae”), because they share similar male reproductive apparatuses composed of prostate glands associated with the male gonopores (cf. [21–23]). They are also morphologically similar in a few respects to the African Eudrilidae.

The status of *Dichogaster* is uncertain from the present results, perhaps largely due to the inclusion of New World species, which have not been included in previous phylogenetic efforts. Two of the three sampled *Dichogaster* species, from Guadeloupe (French West Indies) and from an arboreal epiphyte root mass north of Manaus, Brazil (*Dichogaster* green tree worm), are clearly separated from *D. saliens*, historically endemic to Africa, and both a SOWH test and a Bayesian topology test strongly reject *Dichogaster* monophyly. The latter species has previously been included in a highly supported African and south Pacific *Dichogaster* clade, within the also highly supported Benhamiinae [12]. Morphologically, the New and Old World *Dichogaster* species share many derived characters, but differ on a few points [72]. The geographic distribution of the genus (equatorial Africa, north Neotropics, northern South America, South Pacific) remains enigmatic in the absence of a well resolved and more broadly sampled phylogeny of Benhamiinae.

The classically defined Glossoscolecidae was separated into Rhinodrilidae (“Pontoscolecidae”) and a restricted Glossoscolecidae based on a weakly supported node in the topology recovered in [12]. That node had Almidae intervening between the two families. Our results confirm that node with strong support, suggesting that Almidae is probably secondarily aquatic given that Glossoscolecidae and Rhinodrilidae are predominantly terrestrial. We hypothesize that the common ancestor of Almidae and Rhinodrilidae occurred at a time when paleocontinents made possible the occupation of South American, African and Asian landmasses; South America would seem to be the most probable area of origin for Almidae.

The current study confirmed relationships within Lumbricoidea put forth by [12], and resolved an outstanding conflict about Hormogastridae, which was found to be monophyletic in [65] but paraphyletic or unresolved due to the placement of *Hemigastrodrilus* in [12]. Although analyses of the 75% data set support paraphyly of Hormogastridae, analyses of the 25 and 50% data sets, as well as all “deleted outgroup” data sets, return a monophyletic Hormogastridae (Fig. 2).

Despite the consistent topological patterns seen across all analyses, supernetwork visualization revealed high levels of interlocus conflict (Fig. 3). Some regions of high incongruence—e.g., near the base of Crassiclitellata—are unsurprising, given that concatenated analyses of different data sets recover different relationships in this region of the tree. However, the networks also show a higher level of conflict among loci along the backbone of the Southern Hemisphere subclade than in the Northern Hemisphere group. The reasons for this are unclear, but more taxa were sampled from the Southern Hemisphere clade, and branches in this group on both the network (Fig. 3) and, less obviously, on the PhyloBayes tree (Fig. 1) are generally longer.

### Pangaean earthworms?

No known earthworm fossils exist. Although several ichnofossils have been attributed to earthworm-like organisms, these traces provide little or no concrete information about the clade membership of the author of any hole, burrow, fecal material or other fossilized biostructure made by an elongated soft-bodied invertebrate. However, we can make some inferences about the age of earthworm clades based on the biology and distributions of extant earthworm species and the results of our dating analyses. First, transoceanic movement of adult crassiclitellates seems unlikely except for a few cases where species have become salt-water tolerant inhabitants of marine littoral zones (e.g., *Pontodrilus litoralis*). Transoceanic dispersal of earthworms is nonetheless a possibility over geological time scales—such dispersal events have been inferred for other subterranean terrestrial animals (e.g., amphisbaenians; [73]), earthworm cocoons may be dispersed via rafting or by birds, and earthworms are known from many islands. Second, current earthworm distributions show a high degree of congruence with post-Pangaean continental movements [6, 70]. Third, current earthworm distributions generally show high degrees of local endemicity in topographically complex landscapes, and even in non-complex areas in some lowland tropical forests [74].

Relative ages of New Zealand earthworm clades are comparable to those of continental earthworm faunas [75], and multiple sister-group relationships span large distributional gaps (e.g., New Zealand-Madagascar and trans-Pacific relationships between Australia and North and Central America). Lumbricidae (Eurasia, North America) has been estimated to be about 125 million years old [76] using biogeographic calibrations, while the split between Lumbricoidea and earthworm families on the branch leading to Megascolecoidea was previously estimated at about 200 MYA, the Triassic-Jurassic boundary, coinciding with the separation of Laurasia from Gondwana [77]. The latter split is also present in our trees, with comparable taxon sampling. Our date estimates for this node are somewhat more recent (ranging from 161 to 186 mya, depending on the data matrix; Table 4), but the standard errors on these estimates (±21- ± 23 my) are substantial. However, deletion of the long-branch outgroup taxon ?Haplotaxidae sp. yielded earlier divergence times for this node (~178 mya with >50% gap sites excluded, ~186 mya with all sites) that are more concordant with the breakup of Pangaea.

Recovery of a sister-group relationship between a Laurasian clade and a Gondwanan clade is not unprecedented; similar patterns have been seen in crayfish (Astacoidea and Parastacoidea) [78], dragonflies (Petaluridae) [79], stoneflies (Arctoperlaria and Antarctoperlaria) [80], mayflies (Ephemerelloidea) [81] and squeak beetles (Hygrobiidae) [82]. Within Lumbricidae, the split between European *Eisenia* and a North American clade containing *Eisenoides* and others (not sampled in this study) may be consistent with the final separation of the two continents at ~72 MYA [77].

The clade containing *Komarekiona*, Sparganophilidae, Kynotidae and Biwadrilidae (the latter not sampled in this study) also shows some sign of a northern continent / southern continent split, which also suggests a Pangaean distribution. As above, our divergence time estimate for the split between *Kynotus* and *Komarekiona* + *Sparganophilus* using the 75% data set (165 ± 22 mya) is more recent than we might expect if the Pangaean hypothesis was true. Once again, however, estimates based on the “no ?Haplotaxidae sp.” data matrices (178 and 186 mya) are deeper in time, and the congruence in divergence time estimates for the two focal nodes is noteworthy (for the “no ?Haplotaxidae sp.” data matrices, the mean estimates are within one million years of each other). These are very small families, two from North America and one each from Madagascar and Japan, respectively. There is little evidence to lead us to a hypothesis about the geographic location of the ancestor of Crassiclitellata. Moniligastridae, the sister group of Crassiclitellata represented in this study by *Drawida*, is now only found in South and East Asia. It is extremely diverse in India, but less so elsewhere in Asia as far east as Borneo [83] and Mindoro Island, Philippines (James, unpublished data), an Asian crustal fragment. Based on this distribution, the Moniligastridae–Crassiclitellata divergence would seem most likely to have occurred in a southern landmass.

Our dating analyses seem to be broadly consistent with the hypothesis that the two major “north-south” divergences within Crassiclitellata were caused by the breakup of Pangaea, but they do not constitute a particularly strong test. Additional data and more thorough dating analyses will be required to provide a more rigorous test of the Pangaean breakup hypothesis.

There remain several unanswered questions about the evolutionary history of Clitellata. Within the former, for example, we do not yet have a clear picture of the sister group to Crassiclitellata, nor have we robust support for crassiclitellate monophyly using the data presented here. The shared presence of a multi-layered clitellum remains the strongest evidence for crassiclitellate monophyly, but the possibility of multiple origins of this trait cannot be disregarded. Ongoing phylogenomic work on Clitellata as a whole should shed substantial light on this question.

## Conclusions

This study clarifies earthworm phylogeny and evolution, supporting several recently proposed revisions to our understanding of earthworm relationships and resolving others, most notably including 1) placement of Kynotidae (Madagascar) with a group containing the North American taxa Komarekionidae and Sparganophilidae, 2) a clade comprising Lutodrilidae, Criodrilidae, Hormogastridae and Lumbricidae, 3) *Dichogaster* paraphyly, 4) affirmation of a restricted Glossoscolecidae and 5) Hormgastridae monophyly. Recovery of two major clades, each consisting of a Northern Hemisphere subclade and a Southern Hemisphere subclade, suggested a major role for vicariance (specifically, the breakup of Pangaea during the Mesozoic) in earthworm phylogeny and biogeography. Divergence time estimation provided additional support for this hypothesis, dating the north-south splits within each major clade to ~161–185 Mya.

## Additional files

Additional file 1: Figure S1. Gene occupancy matrices for the original, unfiltered a) 25%, b) 50% and c) 75% data matrices. Black/shaded cells indicate the presence of sequence for a sampled gene fragment (shading represents the proportion of gaps/missing data for that gene fragment; black cells represent complete gene fragments and white cells represent missing gene fragments). Trees depict the maximum likelihood topology from an unpartitioned RAxML analysis. Matrix rows are arranged to reflect estimated relationships; order of matrix columns is arbitrary. (PDF 413 kb)

Additional file 2: Figure S2. ML phylograms with bootstrap support values of all 55 genes (OGs) that were used to construct the 75% data matrix from which “?Haplotaxidae sp.” had been excluded. These genes passed through the TreSpEx and BaCoCa filters described in the text, and included all sites (i.e., sites comprising >50% gaps were not deleted). The title for each tree lists the tree number, the orthogroup number in the HaMStR Lophotrochozoa core ortholog set (e.g., “111,230” for tree 1) and the gene/transcript name (e.g., “C43H8.2” for tree 1). The gene/transcript name can be looked up in online databases (e.g., EnsemblMetazoa; http://metazoa.ensembl.org). For example, for the first tree, C43H8 is a transcript of WBGene00016622, repressor of RNA polymerase III transcription MAF1. (PDF 14509 kb)

Additional file 3: Figure S3. PhyloBayes 50%-majority-rule consensus phylogram for a) the 75% matrix with ?Haplotaxidae sp. removed, filtered with TreSpEx and BaCoCa, all sites, and b) 75% matrix with ?Haplotaxidae sp. removed, filtered with TreSpEx and BaCoCa, and sites comprising >50% gaps deleted. Posterior probabilities are shown at nodes; nodes without values have posterior probabilities of 1.0. (PDF 82 kb)

Additional file 4: Figure S4. Chronograms depicting results of PhyloBayes dating analyses for three 75% data matrices, with highlighted nodes separating *Kynotus* from *Sparganophilus* + *Komarekiona* (node 1) and separating the Northern Hemisphere clade comprising *Lutodrilus* and Lumbricoidea and the clade comprising several Southern Hemisphere families (node 2). Scale bars in millions of years ago. a) Unfiltered 75% matrix including ?Haplotaxidae sp., all sites included b), 75% matrix with ?Haplotaxidae sp. removed, filtered with TreSpEx and BaCoCa, all sites, and c) 75% matrix with ?Haplotaxidae sp. removed, filtered with TreSpEx and BaCoCa, and sites comprising >50% gaps deleted. (PDF 779 kb)
