# Erratum to: Phylogenomic analyses of Crassiclitellata support major Northern and Southern hemisphere clades and a Pangaean origin for earthworms

**DOI:** 10.1186/s12862-017-1042-8

**Published:** 2017-08-25

**Authors:** Frank E. Anderson, Bronwyn W. Williams, Kevin M. Horn, Christer Erséus, Kenneth M. Halanych, Scott R. Santos, Samuel W. James

**Affiliations:** 1 Department of Zoology, Southern Illinois University, Carbondale, IL 62901 USA; 2 North Carolina Museum of Natural Sciences, Research Laboratory, Raleigh, Carolina, North, 27699 USA; 3 Department of Biological and Environmental Sciences, University of Gothenburg, 405 30 Göteborg, SE Sweden; 4 Molette Biology Laboratory for Environmental and Climate Change Studies, Department of Biological Sciences, Auburn University, Auburn, AL 36849 USA; 5 Department of Biology, University of Iowa, Iowa City, Iowa 52242 USA

## Erratum

After publication of the original article [1], the authors received the correct museum catalogue number of *Avelona ligra*, Lumbricidae, which is listed in Table 1. The museum number currently reads MNHN XXXXXX in the original article, but should read MNHN HEL593.

A full, updated version of the amended Table (Table 1) is included in this Erratum.

The original article has been corrected.

**Table 1:** Collection locality, museum location of voucher specimen, museum catalog number, SRA project number, number of Illumina reads, number of Trinity contigs and number of HaMSTr ortholog groups represented for each of the thirty-seven transcriptomes generated in this study

Taxon	Locality	Museum	# Contigs	# HaMStR Orthologs
Acanthodrilidae sp.	Argentina, Tierra del Fuego, Ushuaia (coll. E. Lapied)	NCSM 27264	181,228	1140
*Alma* sp. Almidae	Gabon, edge of Lac Vembo, Gamba complex, (coll. S James 18 May 2008)	NCSM 27265	110,015	558
*Avelona ligra* Lumbricidae	France, Jargeau, Loiret Department, (coll. M. Koken)	MNHN HEL593	182,509	1173
*Criodrilus lacuum* Criodrilidae	Hungary (coll. C. Csuzdi)	NCSM 27266	119,084	934
*Dendrobaena hortensis* Lumbricidae	Sweden, Södermanland, Vingåker, Valltrand, indoor compost, 59.0864 N, 16.0544 E (coll. E. Boräng, 1 Jan 2012)	SMNH 161291 in EtOH CE13942	179,981	1180
*Dichogaster* sp. (green tree worm) Benhamiidae	Brazil, Amazonas, near Manaus, Reserva Campina (coll. S. James, S. Coral, 2 Feb 2012)	NCSM 27267	116,065	1140
*Dichogaster* sp. Benhamiidae	France, Guadeloupe, Basse Terre (colls. S. James, F. Gamiette Feb 2013)	NCSM 27268	106,438	1152
*Dichogaster saliens* Benhamiidae	France, Guadeloupe, Chutes Carbet, Basse Terre (colls. S. James, F. Gamiette Feb 2013)	NCSM 00000	98,665	999
*Drawida* sp. Moniligastridae	USA, Tonganoxie, Kansas (coll. S. James? May 2013)	NCSM 27269	159,219	1081
*Eisenia andrei* Lumbricidae	Unknown	---	137,631	1217
*Eisenia andrei* Lumbricidae	Sweden, Södermanland, Vingåker, Valltrand, indoor compost, 59.0864 N, 16.0544 E (coll. E. Boräng, 1 Jan 2012)	SMNH 161292 in EtOH CE13945	168,836	1191
*Eudrilus eugeniae* Eudrilidae	Brazil, Sao Paulo, bait shop (coll. S. James, 7 Nov 2010)	NCSM 27270	85,990	1008
*Fimoscolex* sp. Glossoscolecidae	Brazil, Assistencia, São Paulo, Fazenda Sta Rosa (coll. S. James, 9 Nov 2012)	NCSM 27271	95,465	705
*Gatesona chaetophora* Lumbricidae	France, Aveyron, L’Hospitalet du Larzac (coll. S James, 1 Mar 2011)	NCSM 27272	104,334	961
*Geogenia benhami* Microchaetidae	South Africa, Western Cape, Stellenbosch (colls S. James, D. Plisko, 27 Aug 2011)	NCSM 27273	84,303	932
*Glossodrilus* sp. Glossoscolecidae	Brazil, Amazonas, near Manaus, Reserva Ducke (colls. S. James, S. Coral 1 Feb 2012)	NCSM 27274	122,993	1053
*Glossoscolex* sp. Glossoscolecidae	Brazil, Parana, Campina Grande do Sul, Caratuva peak trail (coll. S. James, M. Bartz, 17 Oct 2010)	NCSM 27275	58,411	722
*Hemigastrodrilus monicae* Hormogastridae	France, Aveyron, L’Hospitalet du Larzac (coll. S James, 1 Mar 2011)	NCSM 27276	103,338	1098
*Hormogaster elisae* Hormogastridae	SRA PRJNA196484^a^,Spain, El Molar, 40°44′22.9″N, 3°33′53.1″W	---	459,282	1234
*Kerriona* sp. Graciosa1 Ocnerodrilidae	Brazil, Parana, Graciosa Road (coll. S. James, 4 Nov 2010)	NCSM 27277	104,982	1010
*Komarekiona eatoni* Komarekionidae	USA, Sideling Hill Wildlife Mgmt. Area, Washington County, Maryland. (colls. S. James, M. Callaham, May 2013)	NCSM 27278	83,743	1151
*Kynotus pittarelli* Kynotidae	Madagascar, Antsirabe, 19°46′38.60″S 47°06′41.69″E	NCSM 00000	108,836	1073
*Lutodrilus multivesiculatus* Lutodrilidae	USA, Louisiana, Washington Parish (coll S. James, M. Callaham, M. Damoff, C. Erseus, 17 Jan 2011)	NCSM 00000	57,341	1049
*Maoridrilus wilkini* Acanthodrilidae	New Zealand, Kelly’s Creek (coll. T. Buckley)	NCSM 27279	80,910	861
Microchaetidae sp.	South Africa, Western Cape, Tokai Swamp (colls. S. James and D. Plisko, 29 Aug 2011)	NCSM 27280	194,638	1053
*Microchaetus* sp. Microchaetidae	South Africa, Northern Cape, Niewwoudtville (colls. S. James, D. Plisko 5 Sep 2011)	NCSM 27281	125,494	1093
*Parachilota* sp. Acanthodrilidae	South Africa, Western Cape, Table Mountain (coll. James, Meassey, Plisko, 26 Aug 2011)	NCSM 27282	102,971	1074
Place Kabary 2 sp. Acanthodrilidae	Madagascar, Place Kabary, Antsiranana, 12°16′58.27″S 49°17′25.94″E	NCSM 00000	146,018	1157
*Pontodrilus litoralis* Megascolecidae	USA, Cedar Point, Alabama (colls. S. James, C. Erséus 17 January 2011)	NCSM 00000	90,268	1189
*Rhinodrilus priollii* Rhinodrilidae	Brazil, Amazonas, Reserve Ducke (colls. S. James, S. Coral, 3 Feb 2012)	NCSM 00000	87,158	1102
*Scherotheca savignyi* Lumbricidae	France, Midi-Pyrénées, Ariège, Malegoude (coll. S. James, 2 Mar 2011)	NCSM 27283	113,157	1041
*Sparganophilus* sp. Sparganophilidae	USA, Iowa, Des Moines River, at Douds (coll. S. James 12 May 2012)	NCSM 27284	123,905	1199
*Urobenus brasiliensis* Rhinodrilidae	Brazil, Rio Grande do Sul, Santo Cristo (coll. G. Steffen 09 Sep 2009)	NCSM 27285	55,709	890
*Vignysa popi* Hormogastridae	France, Aveyron, Montpellier (colls. S. James, M. Bouche, 1 Mar 2011)	NCSM 27286	93,260	779
Outgroups				
*Delaya leruthi* Haplotaxidae	France, Midi-Pyrénées, Ariège, Cazavet, L’Estelas Cave, in water, 43.000 N, 1.010 E (coll. M.C. des Chatelliers, P. Martin & N. Giani, 24 May 2011) (topotype)	SMNH 161293 in EtOH CE13924	118,020	1067
*Pelodrilus* sp. Haplotaxidae	Western Australia, 25.5 km S of Busselton, Rapids Conservation Park, Margaret River (coll. C. Erséus, 16 Sep 2012)	WAM V9004	100,864	1129
*Haplotaxis gordioides* Haplotaxidae	Sweden, Västergötland, Göteborg, seeping groundwater at Göteborg Botanical Garden (Vitsippsdalen), 57.6813 N, 11.9562 E (C. Erséus & A. Achurra, 29 Mar 2011)	SMNH 161294 in EtOH CE11200	53,878	855
*?*Haplotaxidae sp.	Brazil, Amazonas, Reserva Ducke (colls. S. James, S. Coral, 3 Feb 2012) (topotype)	NCSM 000000 in EtOH CE14372	93,548	1053
*Lumbriculus variegatus* Lumbriculidae	Sweden, Västergötland, Göteborg, Guldheden, spring S of Dr. Fries Torg, 57.6827 N, 11.9707 E (coll. M. Svensson, 8 Nov 2011)	SMNH 161295 slide CE13679	109,949	985
*Propappus volki* Propappidae	Sweden, Blekinge, Ronneby, Väby, Bräkneån River, sand in rapids, 56.1792 N, 15.1052 E (C. Erséus, B. Williams & S. Martinsson, 31 May 2013) (topotype)	SMNH 161296 slide CE18375	131,574	1140

^a^numbers of contigs and orthologs pooled across transcriptomes from three tissue types; see [85] for details. MNHN = National Museum of Natural History (Paris, France); NCSM = North Carolina Museum of Natural Sciences; SMNH = Swedish Museum of Natural History; WAM = Western Australian Museum; some specimens include preservation type and co-author Erséus’s specimen ID numbers (CE#####)
