# Supplementary material for: Phylogenomic analyses of Crassiclitellata support major Northern and Southern Hemisphere clades and a Pangaean origin for earthworms
Source: BMC Evol Biol. 2017 May 30;17:123. doi: 10.1186/s12862-017-0973-4 (PMC5450073; doi:10.1186/s12862-017-0973-4)
Supplement: Supplementary file 1 — Gene occupancy matrices for the original, unfiltered a) 25%, b) 50% and c) 75% data matrices. Black/shaded cells indicate the presence of sequence for a sampled gene fragment (shading represents the proportion of gaps/missing data for that gene fragment; black cells represent complete gene fragments and white cells represent missing gene fragments). Trees depict the maximum likelihood topology from an unpartitioned RAxML analysis. Matrix rows are arranged to reflect estimated relationships; order of matrix columns is arbitrary. (PDF 413 kb) [file 12862_2017_973_MOESM1_ESM.pdf]

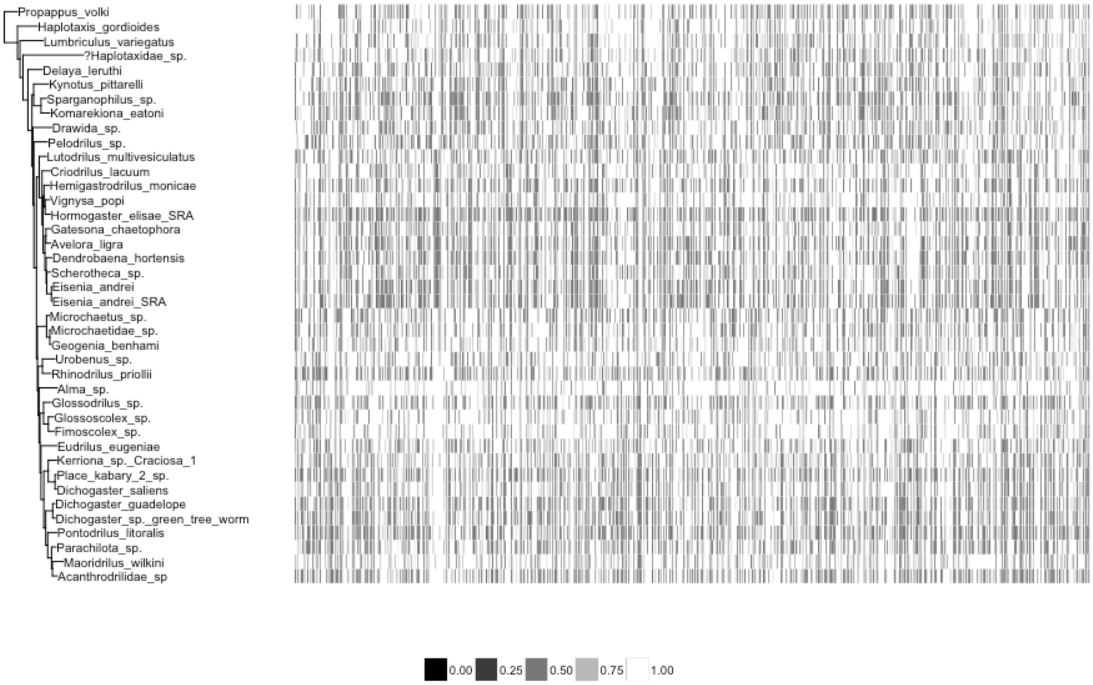
Propappus_volki
Haplotaxis_gordioides
Lumbriculus_variegatus
?Haplotaxidae_sp.
Delaya_leruthi
Kynotus_pittarelli
Sparganophilus_sp.
Komarekiona_eatoni
Drawida_sp.
Pelodrilus_sp.
Lutodrilus_multivesiculatus
Criodrilus_lacuum
Hemigastrodrilus_monicae
Vignysa_popi
Hormogaster_elisae_SRA
Gatesona_chaetophora
Avelora_ligra
Dendrobaena_hortensis
Scherotheca_sp.
Eisenia_andrei
Eisenia_andrei_SRA
Microchaetus_sp.
Microchaetidae_sp.
Geogenia_benhami
Urobenus_sp.
Rhinodrilus_priollii
Alma_sp.
Glossodrilus_sp.
Glossoscolex_sp.
Fimoscolex_sp.
Eudrilus_eugeniae
Kerriona_sp._Craciosa_1
Place_kabary_2_sp.
Dichogaster_saliens
Dichogaster_guadelope
Dichogaster_sp._green_tree_worm
Pontodrilus_litoralis
Parachilota_sp.
Maoridrilus_wilkini
Acanthrodrilidae_sp
0.00
0.25
0.50
0.75
1.00

a

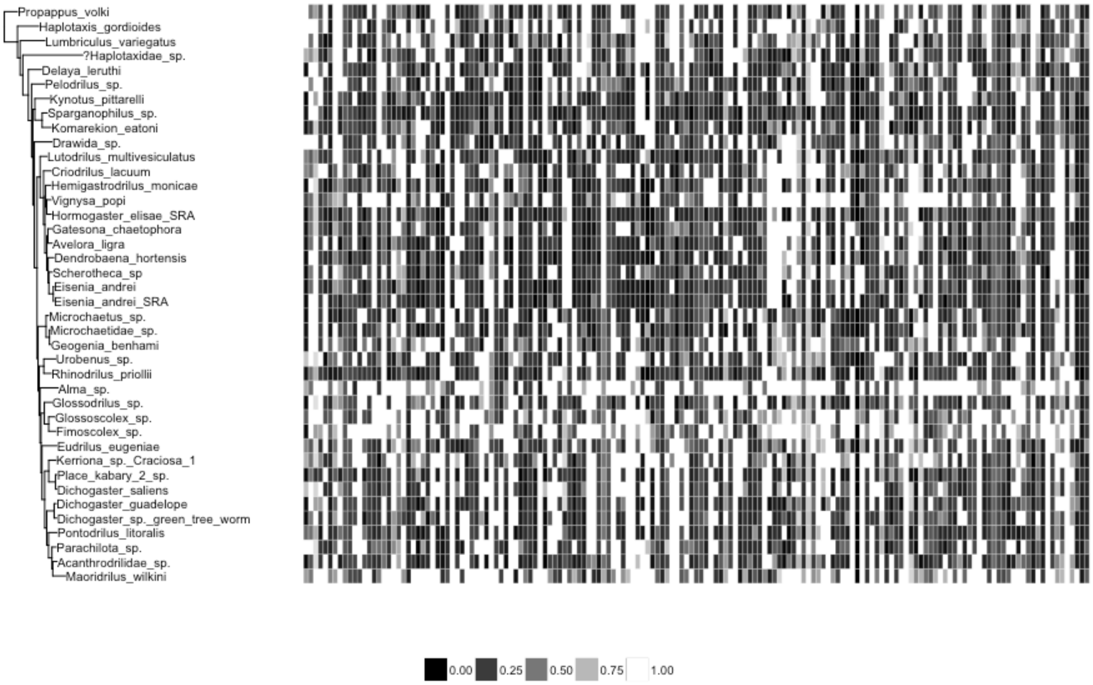
Propappus_volki
Haplotaxis_gordioides
Lumbriculus_variegatus
?Haplotaxidae_sp.
Delaya_leruthi
Pelodrilus_sp.
Kynotus_pittarelli
Sparganophilus_sp.
Komarekion_eatoni
Drawida_sp.
Lutodrilus_multivesiculatus
Criodrilus_lacuum
Hemigastrodrilus_monicae
Vignysa_popi
Hormogaster_elisae_SRA
Gatesona_chaetophora
Avelora_ligra
Dendrobaena_hortensis
Scherotheca_sp
Eisenia_andrei
Eisenia_andrei_SRA
Microchaetus_sp.
Microchaetidae_sp.
Geogenia_benhami
Urobenus_sp.
Rhinodrilus_priollii
Alma_sp.
Glossodrilus_sp.
Glossoscolex_sp.
Fimoscolex_sp.
Eudrilus_eugeniae
Kerriona_sp._Craciosa_1
Place_kabary_2_sp.
Dichogaster_saliens
Dichogaster_guadelope
Dichogaster_sp._green_tree_worm
Pontodrilus_litoralis
Parachilota_sp.
Acanthrodrilidae_sp.
Maoridrilus_wilkini
0.00
0.25
0.50
0.75
1.00

b

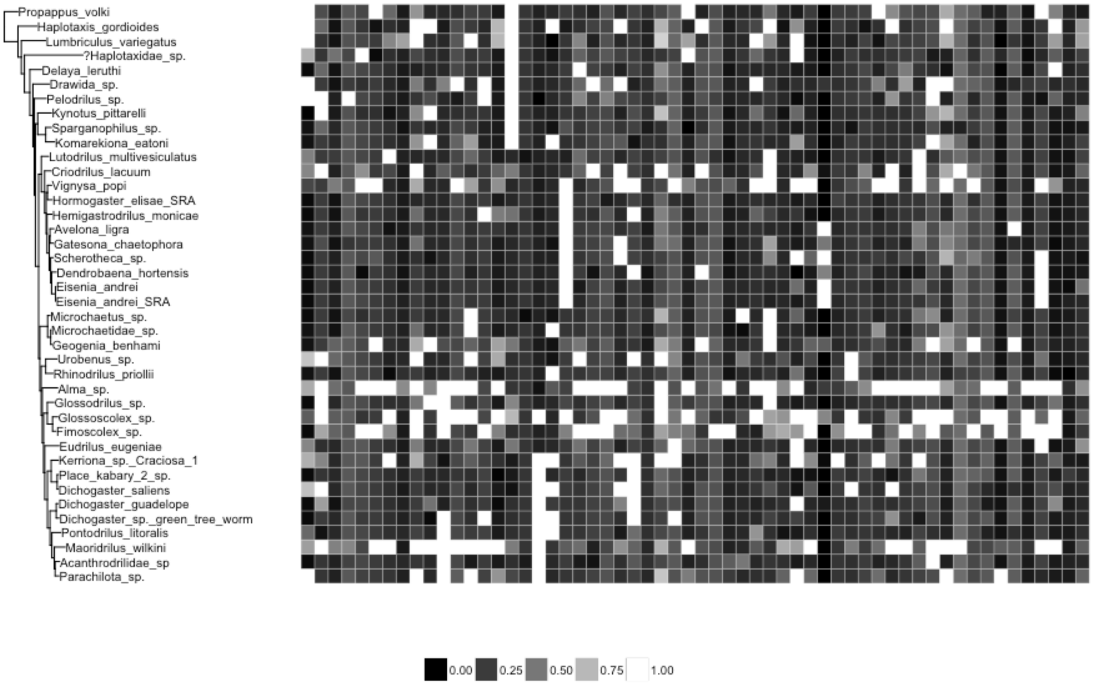
Propappus_volki
Haplotaxis_gordioides
Lumbriculus_variegatus
?Haplotaxidae_sp.
Delaya_leruthi
Drawida_sp.
Pelodrilus_sp.
Kynotus_pittarelli
Sparganophilus_sp.
Komarekiona_eatoni
Lutodrilus_multivesiculatus
Criodrilus_lacuum
Vignysa_popi
Hormogaster_elisae_SRA
Hemigastrodrilus_monicae
Avelona_ligra
Gatesona_chaetophora
Scherotheca_sp.
Dendrobaena_hortensis
Eisenia_andrei
Eisenia_andrei_SRA
Microchaetus_sp.
Microchaetidae_sp.
Geogenia_benhami
Urobenus_sp.
Rhinodrilus_priollii
Alma_sp.
Glossodrilus_sp.
Glossoscolex_sp.
Fimoscolex_sp.
Eudrilus_eugeniae
Kerriona_sp._Craciosa_1
Place_kabary_2_sp.
Dichogaster_saliens
Dichogaster_guadelope
Dichogaster_sp._green_tree_worm
Pontodrilus_litoralis
Maoridrilus_wilkini
Acanthrodrilidae_sp
Parachilota_sp.
0.00
0.25
0.50
0.75
1.00

c
