# Supplementary material for: Phylogenomic analyses of Crassiclitellata support major Northern and Southern Hemisphere clades and a Pangaean origin for earthworms
Source: BMC Evol Biol. 2017 May 30;17:123. doi: 10.1186/s12862-017-0973-4 (PMC5450073; doi:10.1186/s12862-017-0973-4)

111230_C43H8

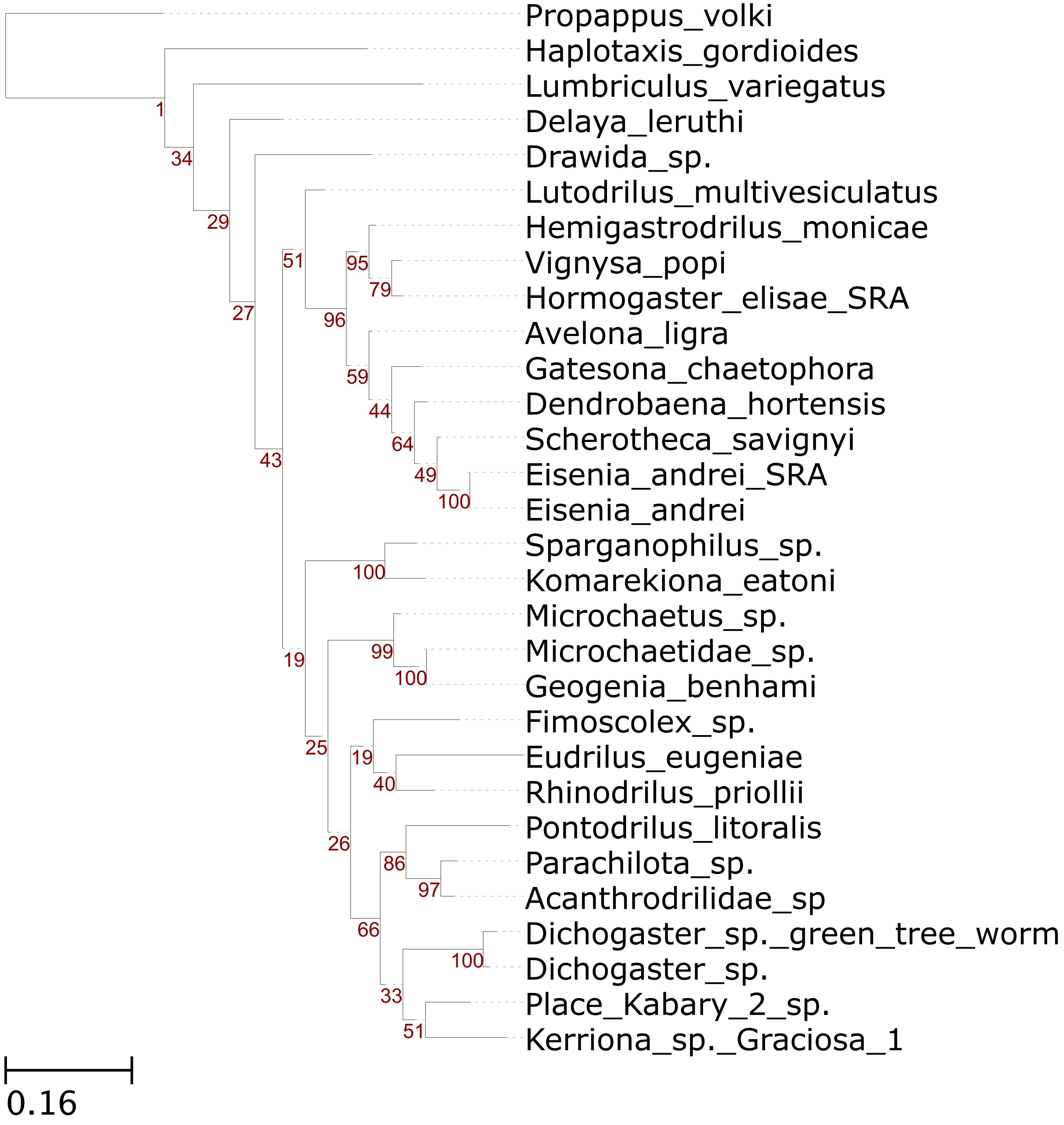

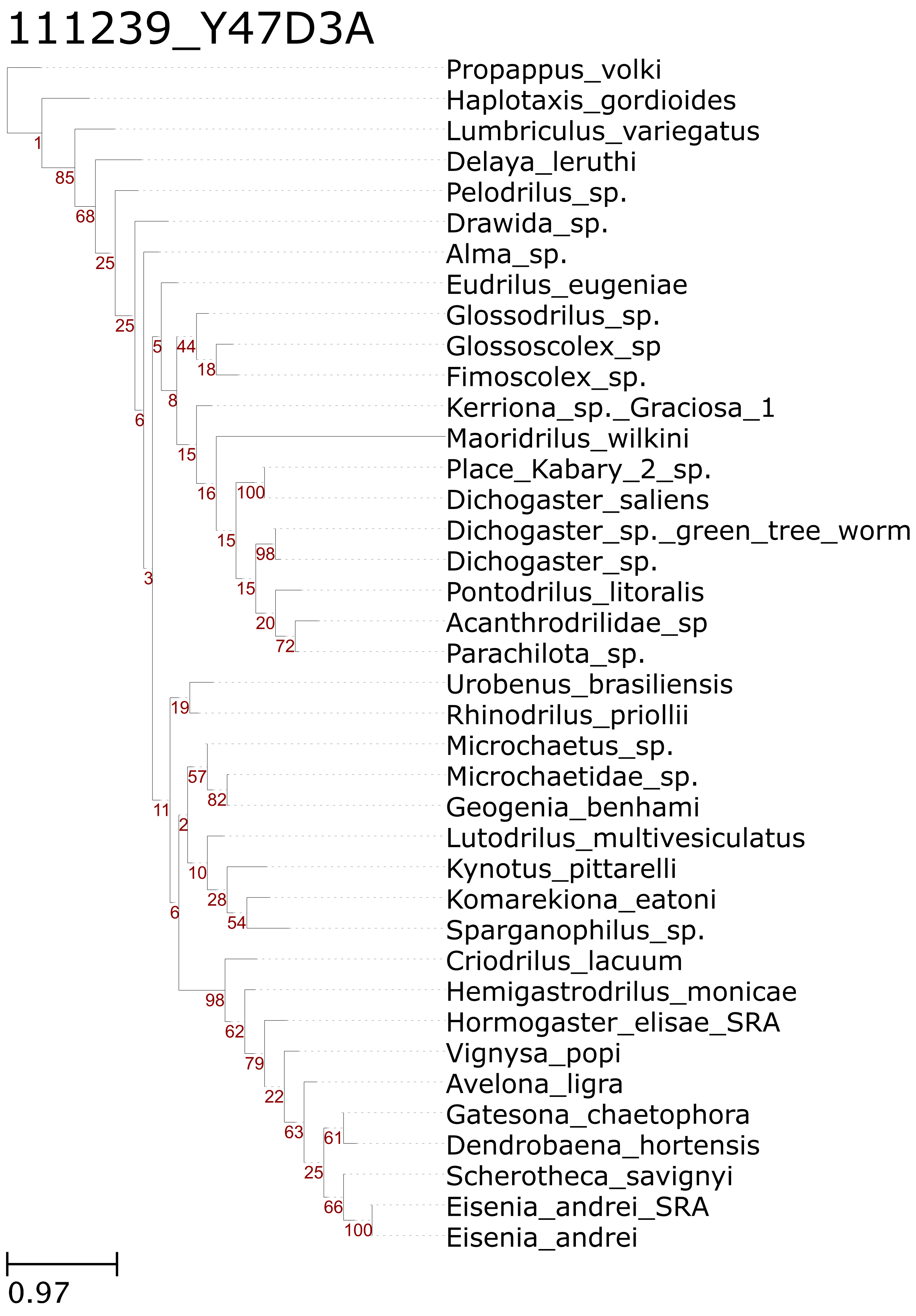
111239_Y47D3A
Propappus_volki
Haplotaxis_gordioides
Lumbriculus_variegatus
Delaya_leruthi
Pelodrilus_sp.
Drawida_sp.
Alma_sp.
Eudrilus_eugeniae
Glossodrilus_sp.
Glossoscolex_sp
Fimoscolex_sp.
Kerriona_sp._Graciosa_1
Maoridrilus_wilkini
Place_Kabary_2_sp.
Dichogaster_saliens
Dichogaster_sp._green_tree_worm
Dichogaster_sp.
Pontodrilus_litoralis
Acanthrodrilidae_sp
Parachilota_sp.
Urobenus_brasiliensis
Rhinodrilus_priollii
Microchaetus_sp.
Microchaetidae_sp.
Geogenia_benhami
Lutodrilus_multivesiculatus
Kynotus_pittarelli
Komarekiona_eatoni
Sparganophilus_sp.
Criodrilus_lacuum
Hemigastrodrilus_monicae
Hormogaster_elisae_SRA
Vignysa_popi
Avelona_ligra
Gatesona_chaetophora
Dendrobaena_hortensis
Scherotheca_savignyi
Eisenia_andrei_SRA
Eisenia_andrei
1
85
68
25
25
5
44
18
8
6
15
16
100
15
98
3
15
20
72
19
57
82
11
2
10
28
54
6
98
62
79
22
63
61
25
66
100
0.97

111242_B0564

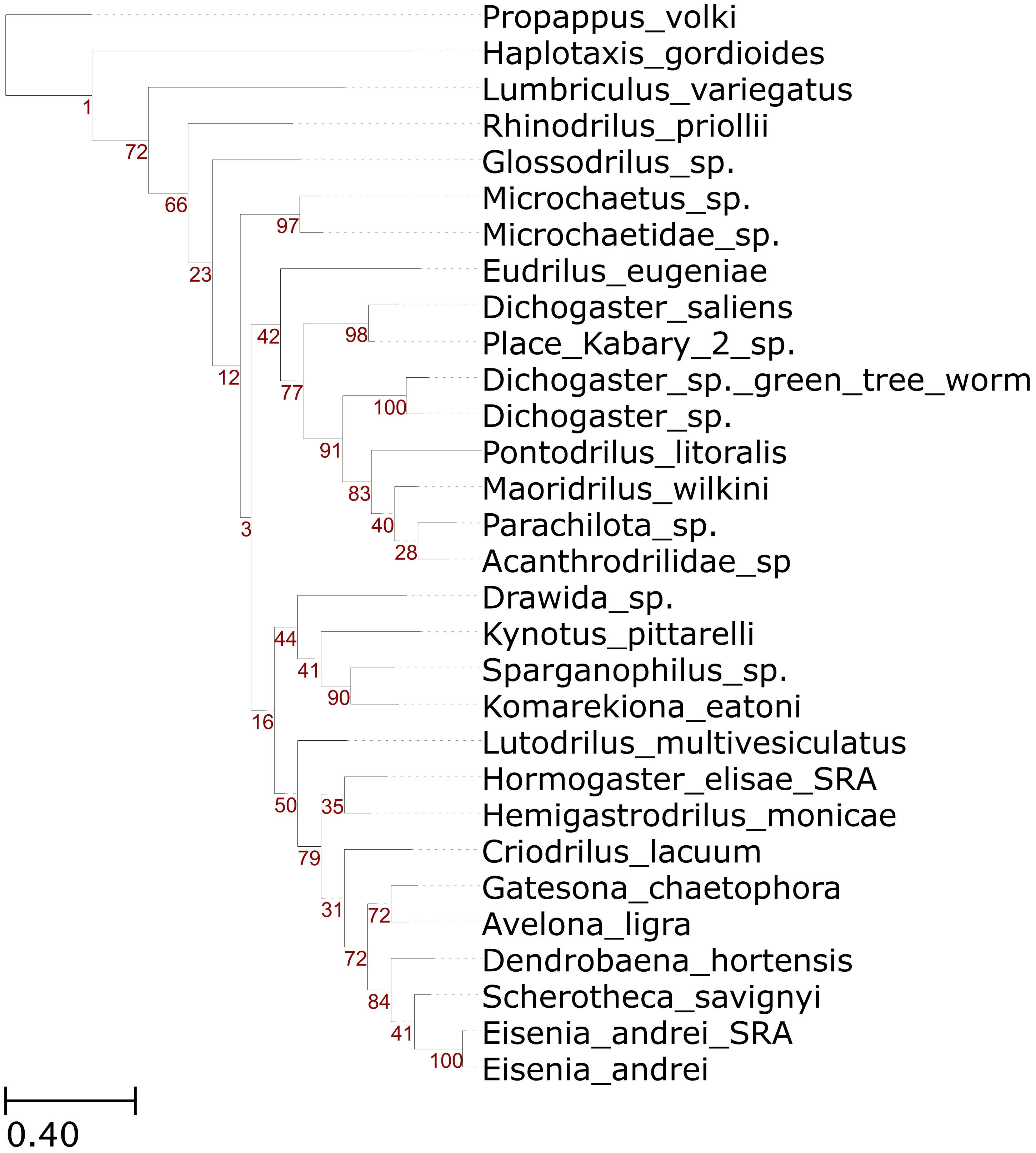

111263_Y106G6H

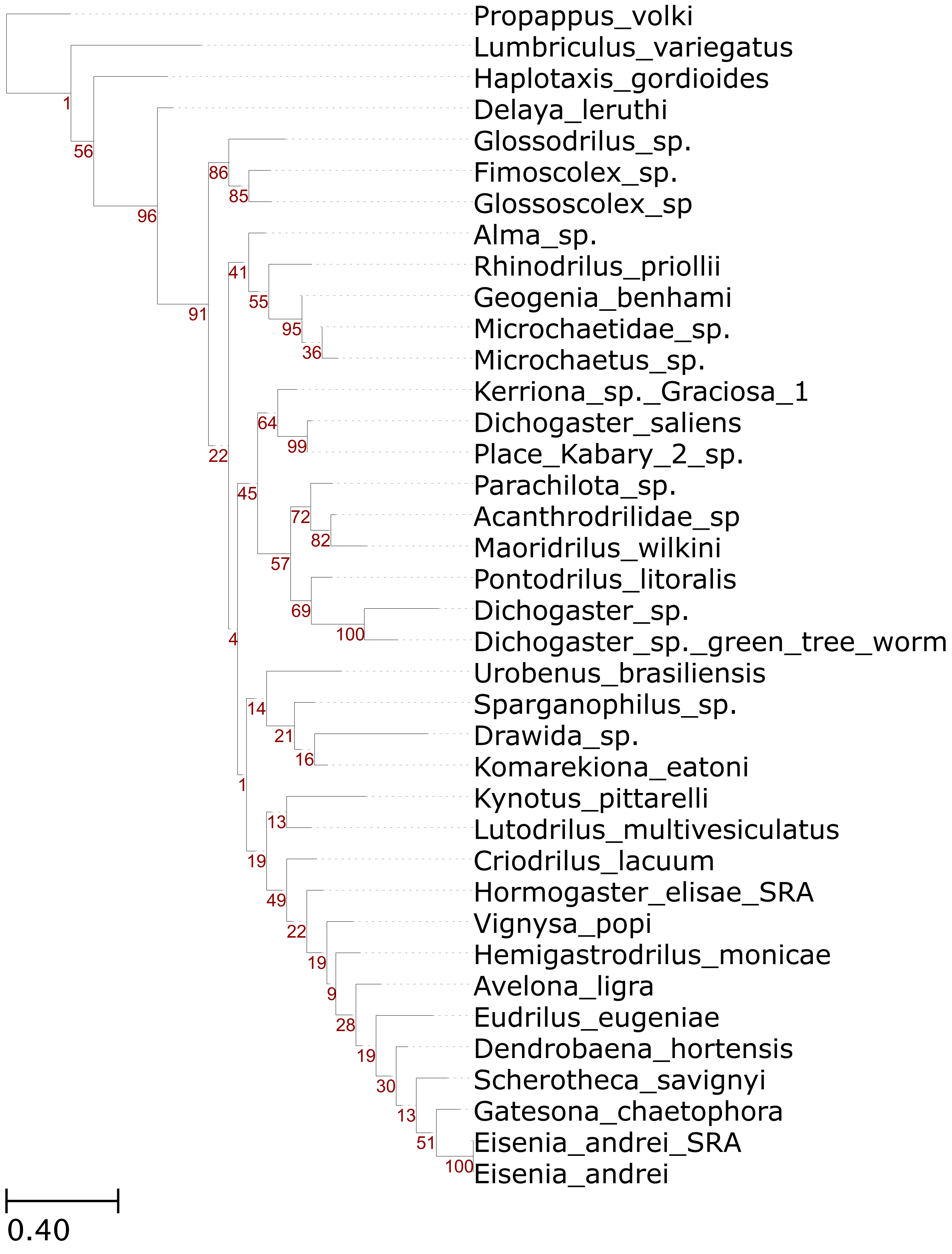

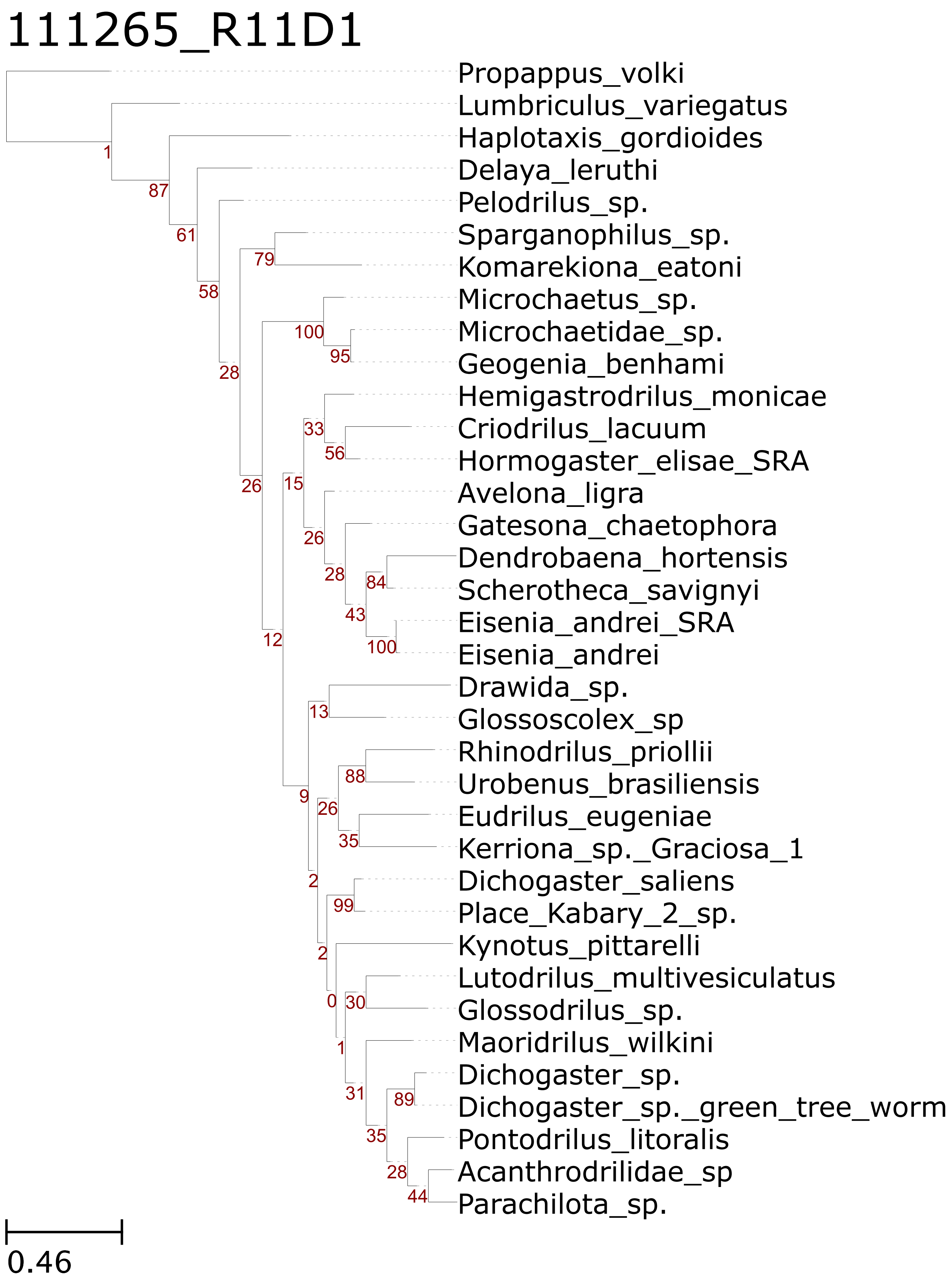
111265_R11D1
Propappus_volki
Lumbriculus_variegatus
Haplotaxis_gordioides
Delaya_leruthi
Pelodrilus_sp.
Sparganophilus_sp.
Komarekiona_eatoni
Microchaetus_sp.
Microchaetidae_sp.
Geogenia_benhami
Hemigastrodrilus_monicae
Criodrilus_lacuum
Hormogaster_elisae_SRA
Avelona_ligra
Gatesona_chaetophora
Dendrobaena_hortensis
Scherotheca_savignyi
Eisenia_andrei_SRA
Eisenia_andrei
Drawida_sp.
Glossoscolex_sp
Rhinodrilus_priollii
Urobenus_brasiliensis
Eudrilus_eugeniae
Kerriona_sp._Graciosa_1
Dichogaster_saliens
Place_Kabary_2_sp.
Kynotus_pittarelli
Lutodrilus_multivesiculatus
Glossodrilus_sp.
Maoridrilus_wilkini
Dichogaster_sp.
Dichogaster_sp._green_tree_worm
Pontodrilus_litoralis
Acanthrodrilidae_sp
Parachilota_sp.
1
87
61
58
79
28
100
95
26
33
56
15
26
28
84
43
100
12
13
88
9
26
35
2
99
2
0
30
1
31
89
35
28
44
0.46

111291_F44E7

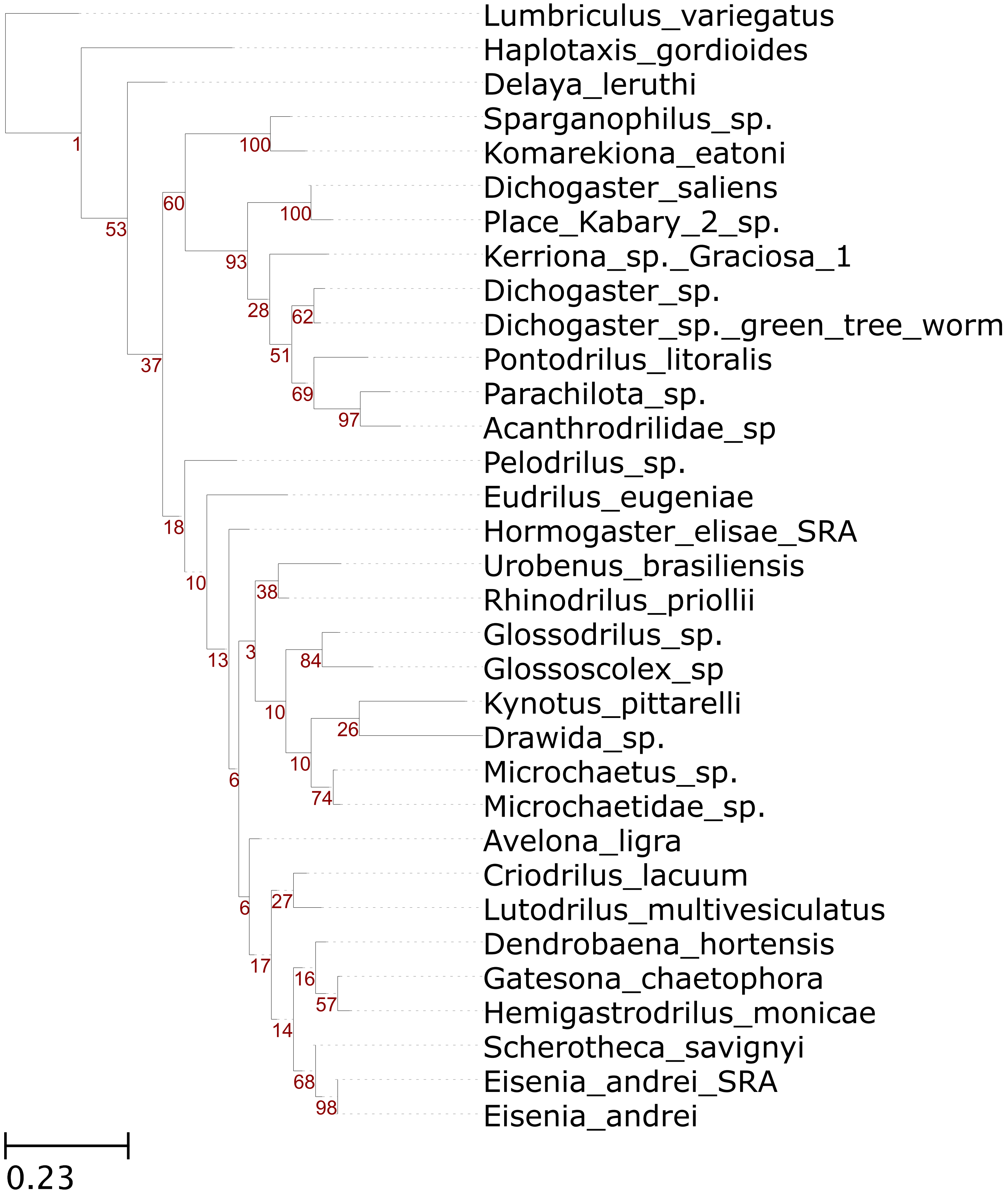

111321_Y66D12A

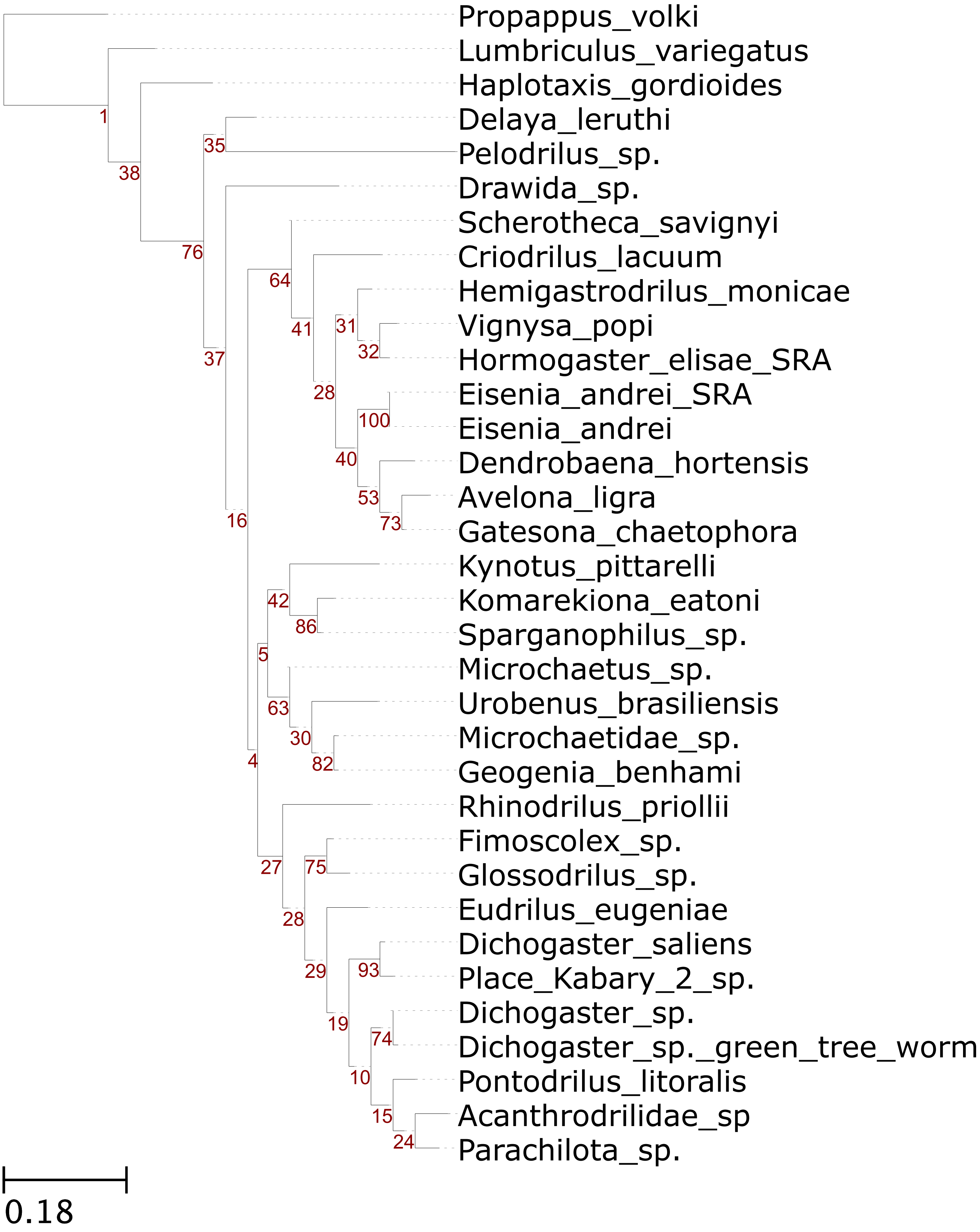

111330_F32D8

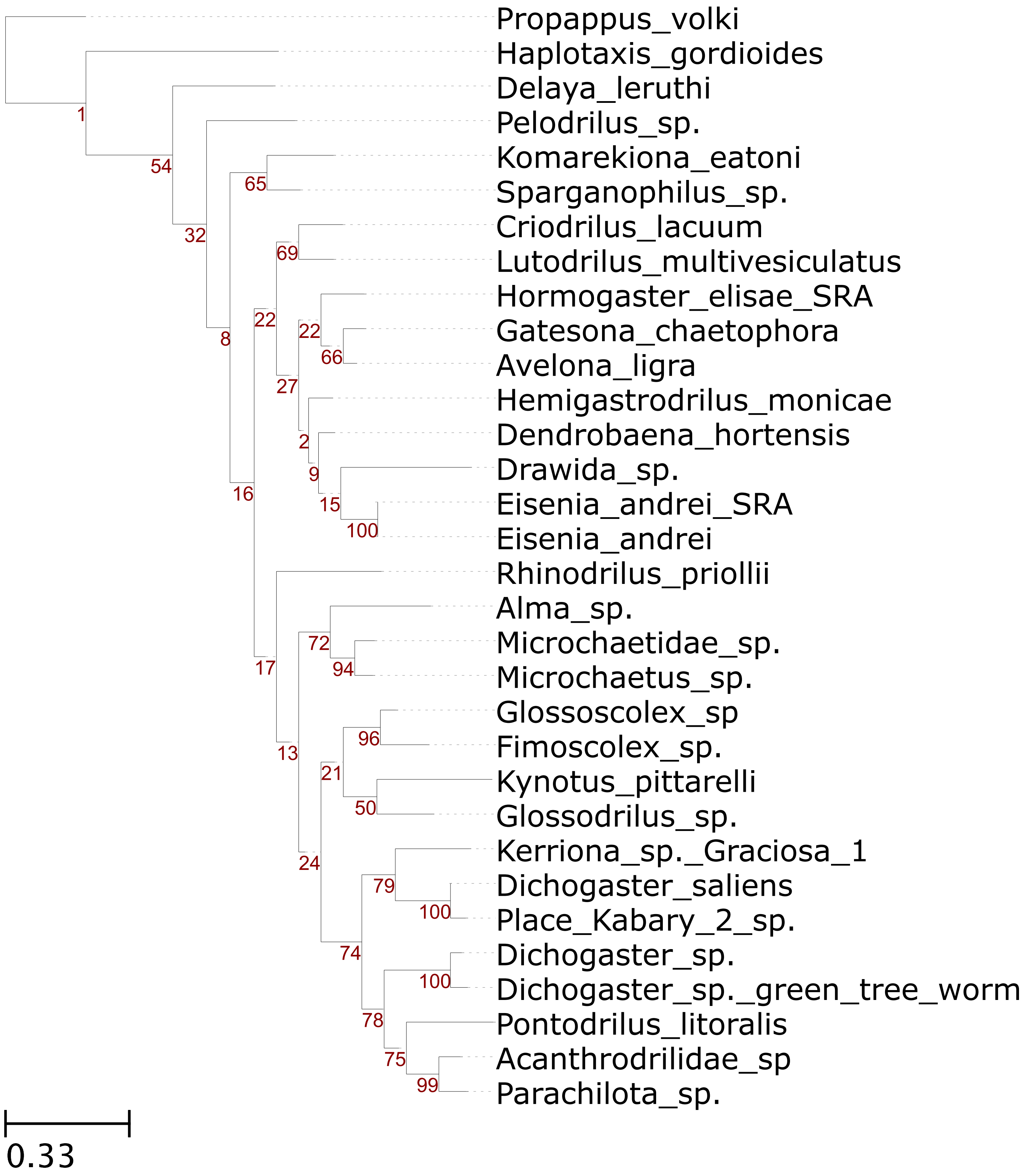

111335_C47B2

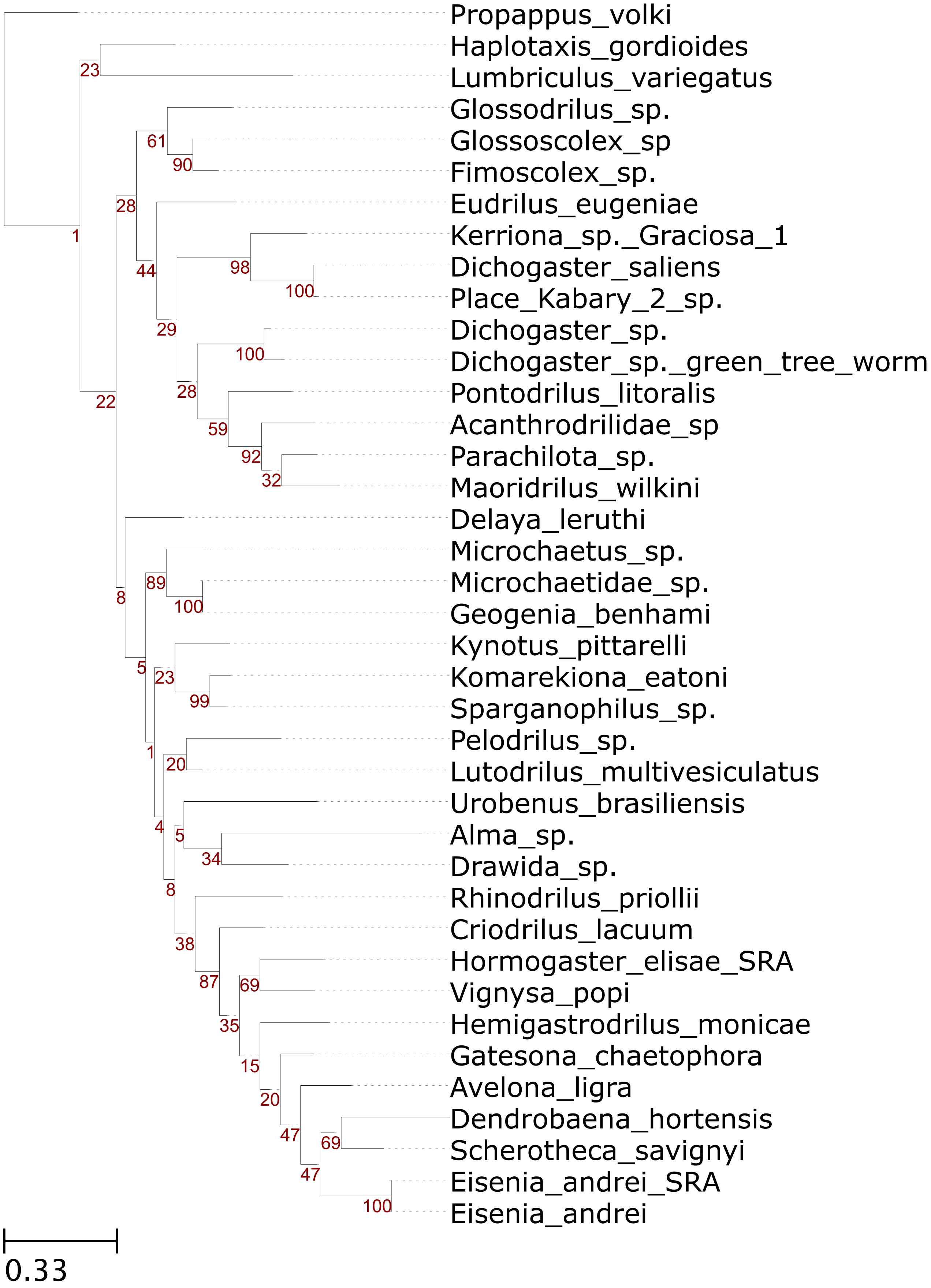

111366_CD4

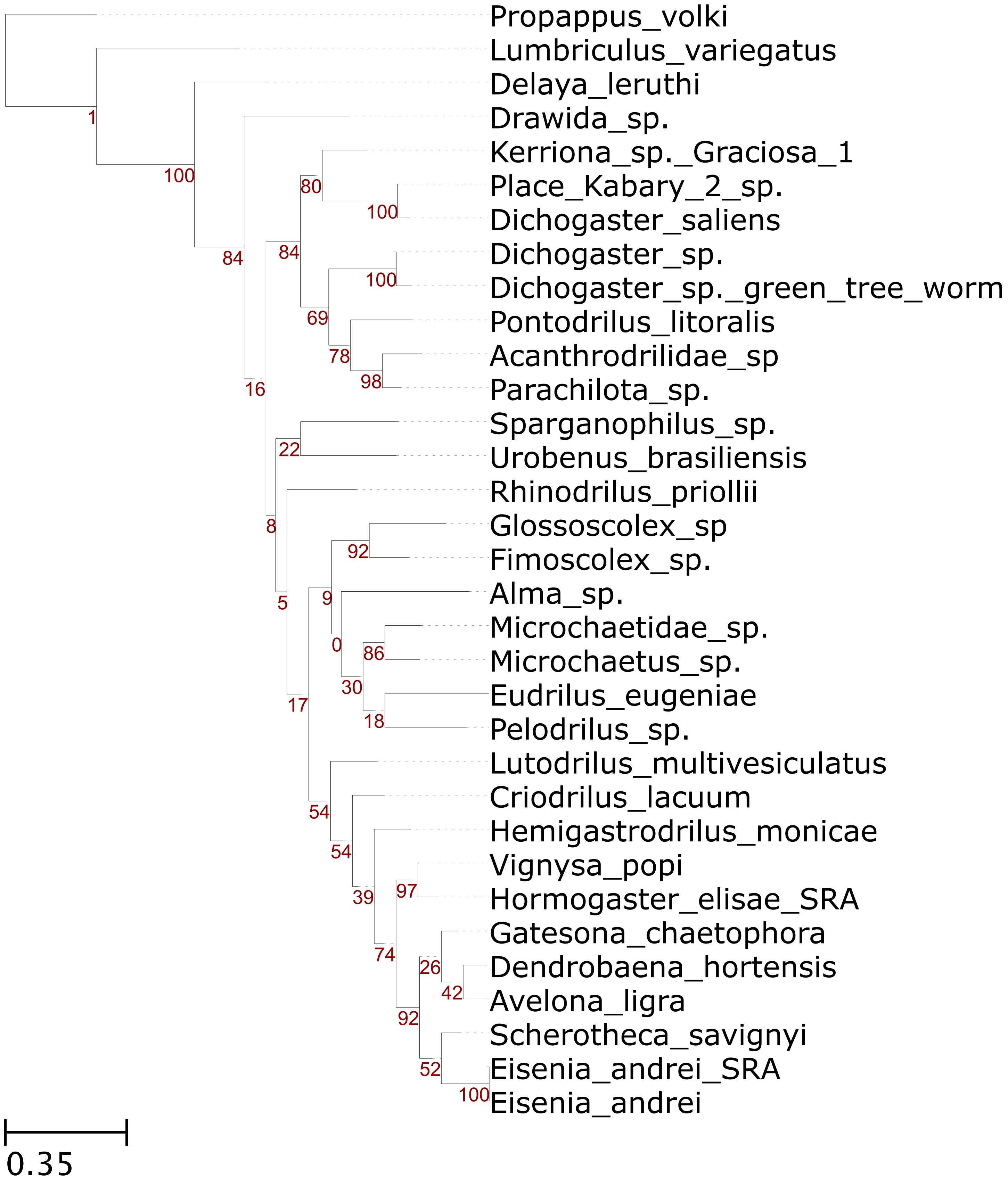

Propappus_volki
Lumbriculus_variegatus
Delaya_leruthi
Drawida_sp.
Kerriona_sp._Graciosa_1
Place_Kabary_2_sp.
Dichogaster_saliens
Dichogaster_sp.
Dichogaster_sp._green_tree_worm
Pontodrilus_litoralis
Acanthrodrilidae_sp
Parachilota_sp.
Sparganophilus_sp.
Urobenus_brasiliensis
Rhinodrilus_priollii
Glossoscolex_sp
Fimoscolex_sp.
Alma_sp.
Microchaetidae_sp.
Microchaetus_sp.
Eudrilus_eugeniae
Pelodrilus_sp.
Lutodrilus_multivesiculatus
Criodrilus_lacuum
Hemigastrodrilus_monicae
Vignysa_popi
Hormogaster_elisae_SRA
Gatesona_chaetophora
Dendrobaena_hortensis
Avelona_ligra
Scherotheca_savignyi
Eisenia_andrei_SRA
Eisenia_andrei
1
100
84
16
8
5
17
80
100
84
100
69
78
98
22
92
9
0
86
30
18
54
54
39
97
74
26
42
92
52
100
0.35

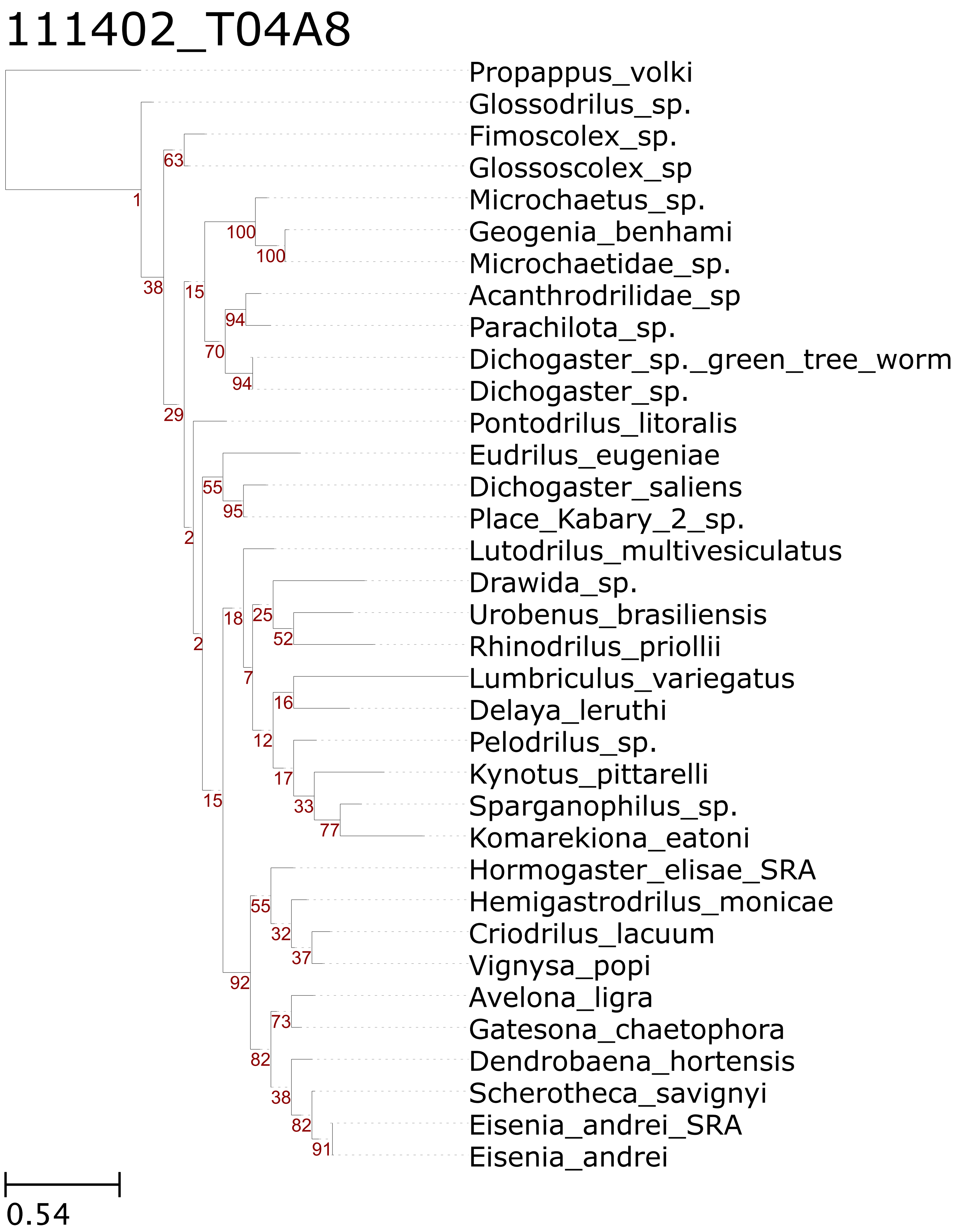
111402_T04A8
Propappus_volki
Glossodrilus_sp.
Fimoscolex_sp.
Glossoscolex_sp
Microchaetus_sp.
Geogenia_benhami
Microchaetidae_sp.
Acanthrodrilidae_sp
Parachilota_sp.
Dichogaster_sp._green_tree_worm
Dichogaster_sp.
Pontodrilus_litoralis
Eudrilus_eugeniae
Dichogaster_saliens
Place_Kabary_2_sp.
Lutodrilus_multivesiculatus
Drawida_sp.
Urobenus_brasiliensis
Rhinodrilus_priollii
Lumbriculus_variegatus
Delaya_leruthi
Pelodrilus_sp.
Kynotus_pittarelli
Sparganophilus_sp.
Komarekiona_eatoni
Hormogaster_elisae_SRA
Hemigastrodrilus_monicae
Criodrilus_lacuum
Vignysa_popi
Avelona_ligra
Gatesona_chaetophora
Dendrobaena_hortensis
Scherotheca_savignyi
Eisenia_andrei_SRA
Eisenia_andrei
0.54

111407_ZK1010

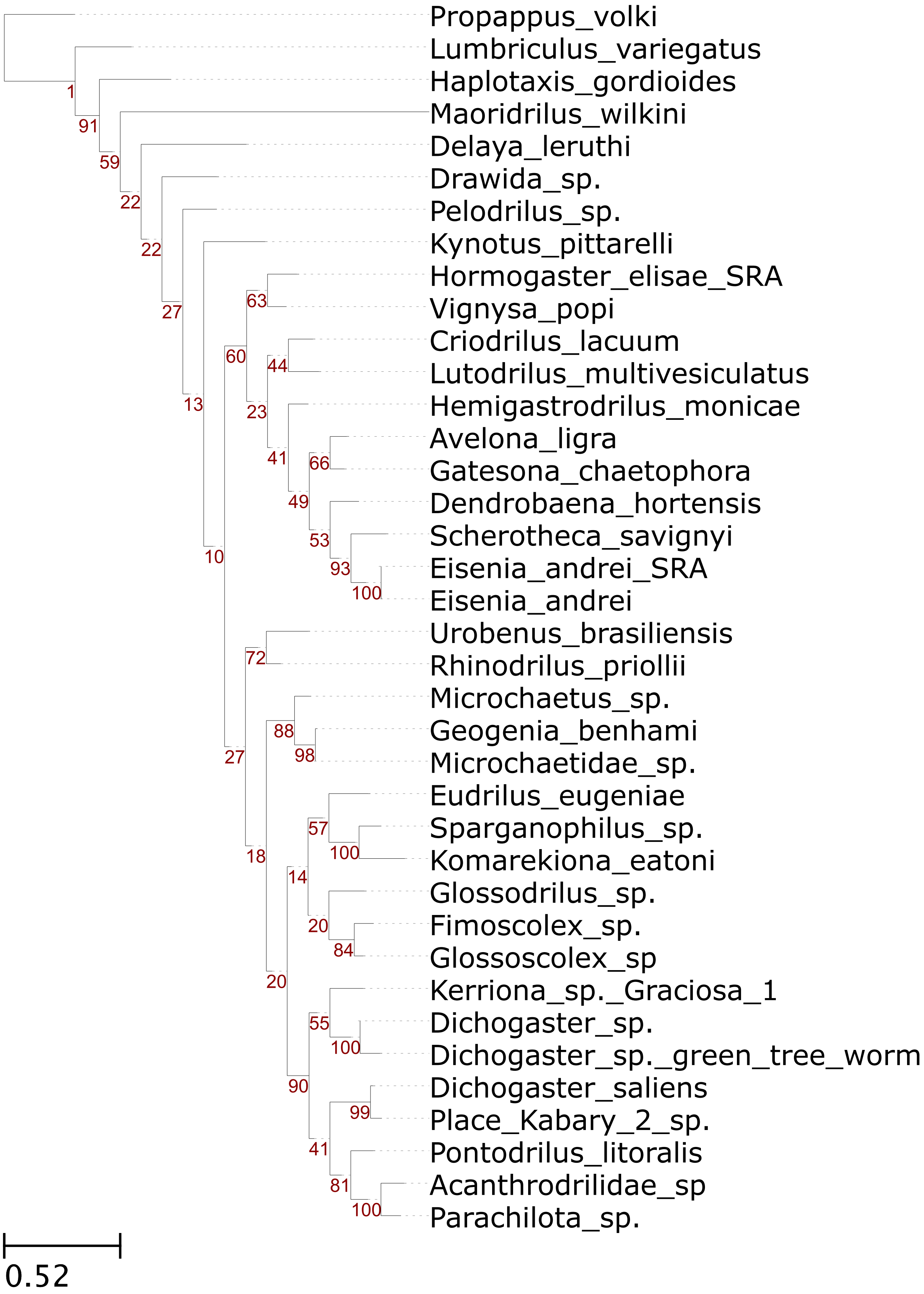

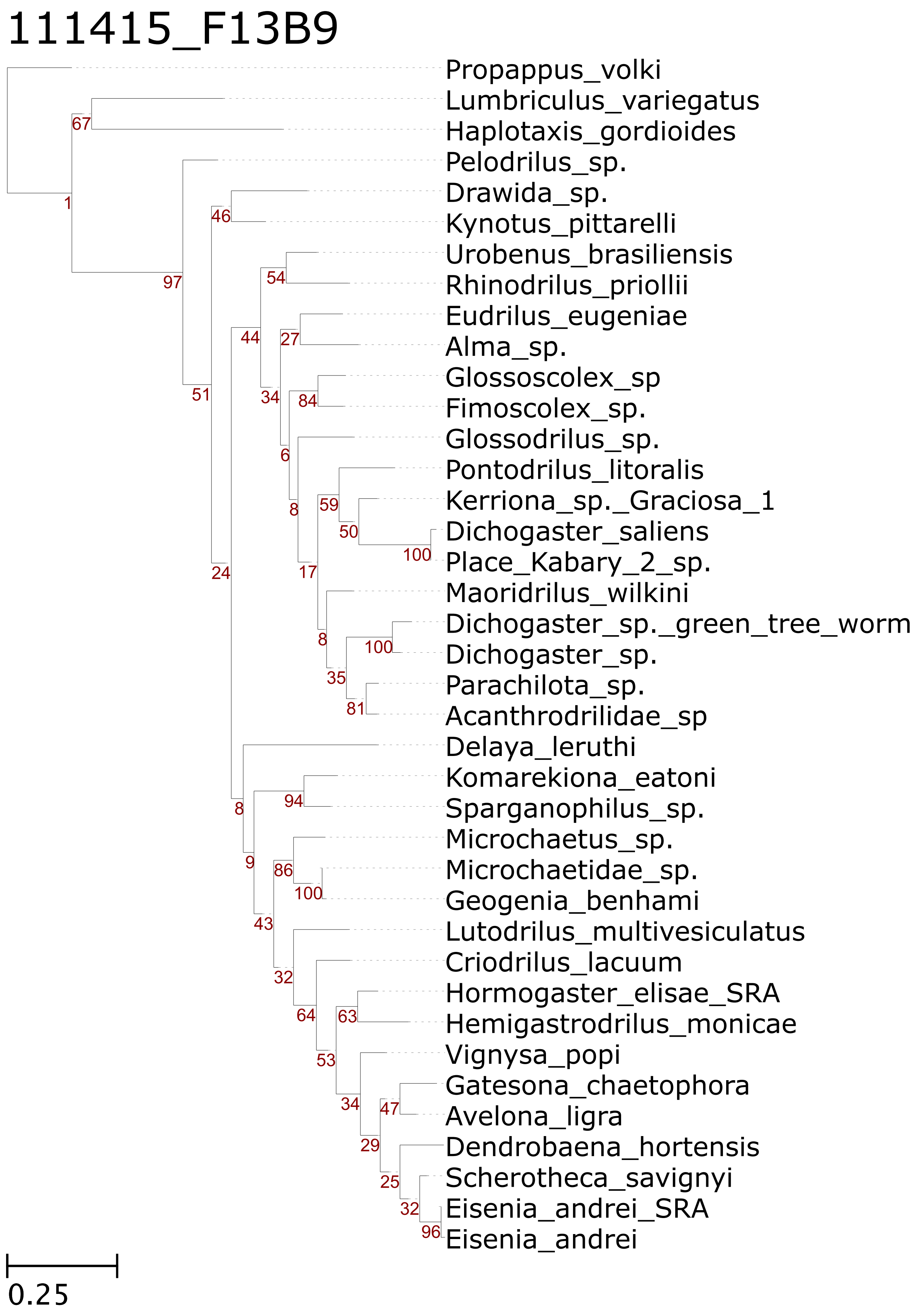
111415_F13B9
Propappus_volki
Lumbriculus_variegatus
Haplotaxis_gordioides
Pelodrilus_sp.
Drawida_sp.
Kynotus_pittarelli
Urobenus_brasiliensis
Rhinodrilus_priollii
Eudrilus_eugeniae
Alma_sp.
Glossoscolex_sp
Fimoscolex_sp.
Glossodrilus_sp.
Pontodrilus_litoralis
Kerriona_sp._Graciosa_1
Dichogaster_saliens
Place_Kabary_2_sp.
Maoridrilus_wilkini
Dichogaster_sp._green_tree_worm
Dichogaster_sp.
Parachilota_sp.
Acanthrodrilidae_sp
Delaya_leruthi
Komarekiona_eatoni
Sparganophilus_sp.
Microchaetus_sp.
Microchaetidae_sp.
Geogenia_benhami
Lutodrilus_multivesiculatus
Criodrilus_lacuum
Hormogaster_elisae_SRA
Hemigastrodrilus_monicae
Vignysa_popi
Gatesona_chaetophora
Avelona_ligra
Dendrobaena_hortensis
Scherotheca_savignyi
Eisenia_andrei_SRA
Eisenia_andrei
67
1
97
46
51
54
44
27
34
84
6
8
59
50
100
17
24
8
100
35
81
8
94
9
86
100
43
32
64
63
53
34
47
29
25
32
96
0.25

111417_F46B6

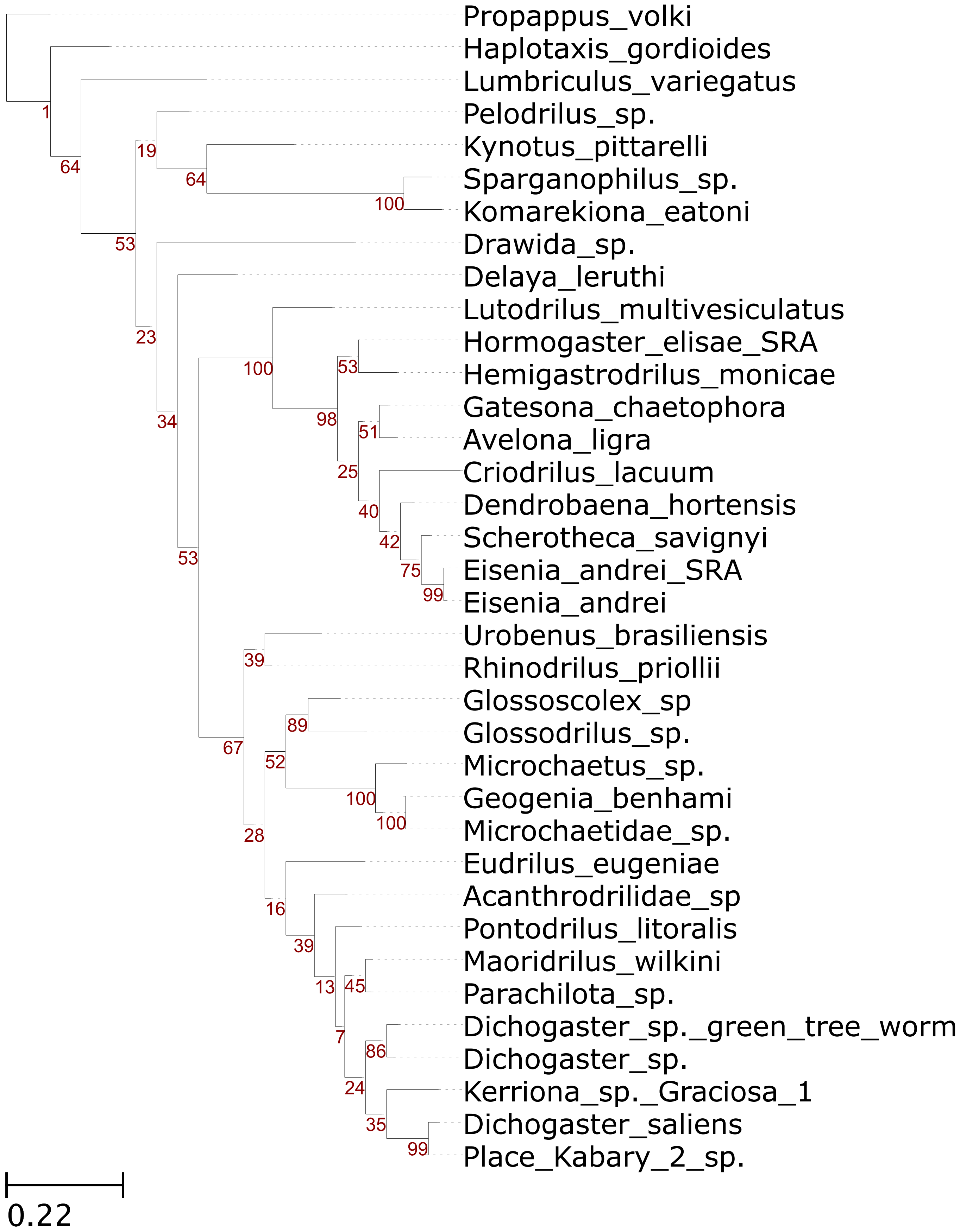

111432_C02F5

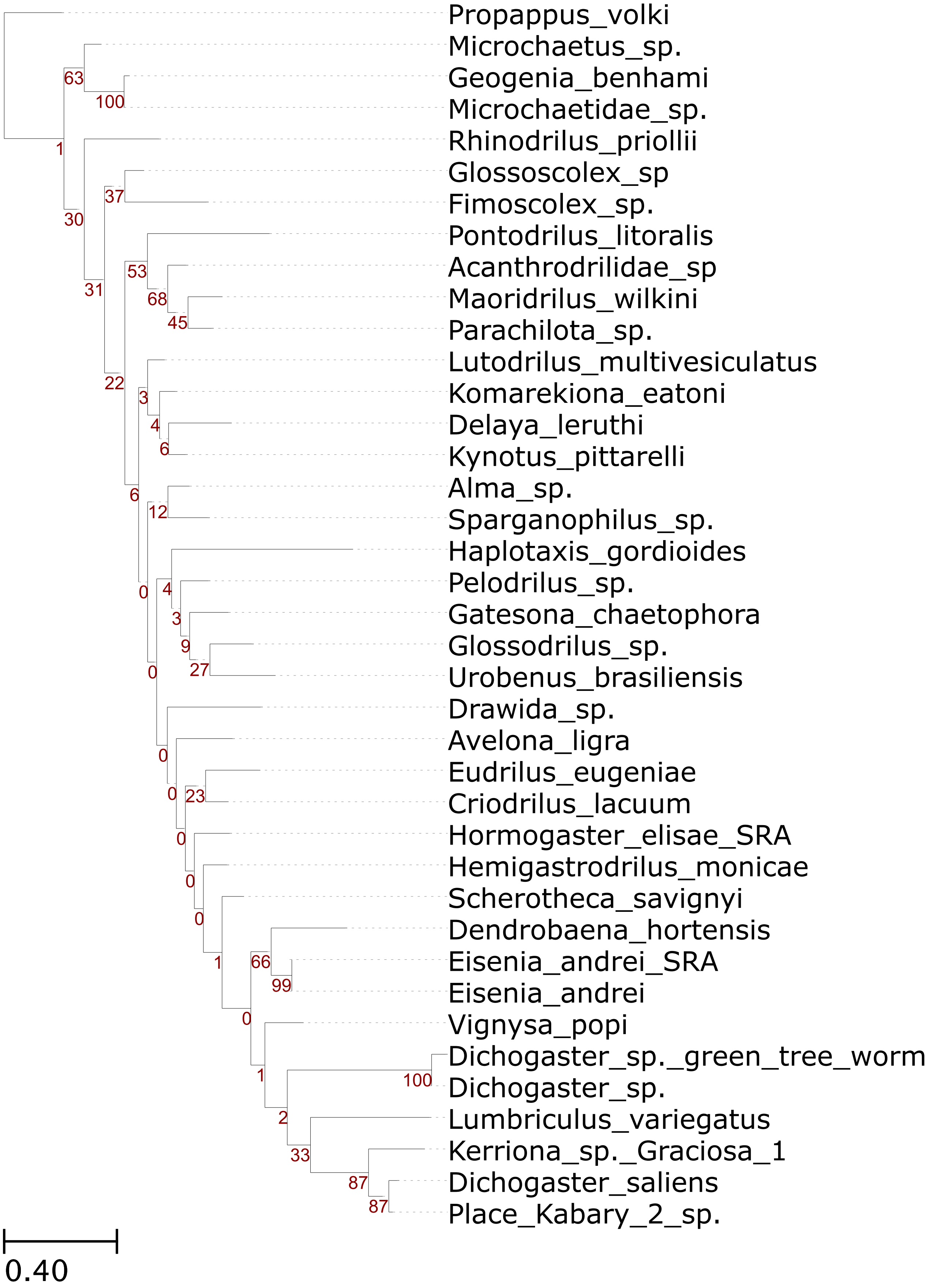

111434_T03F1

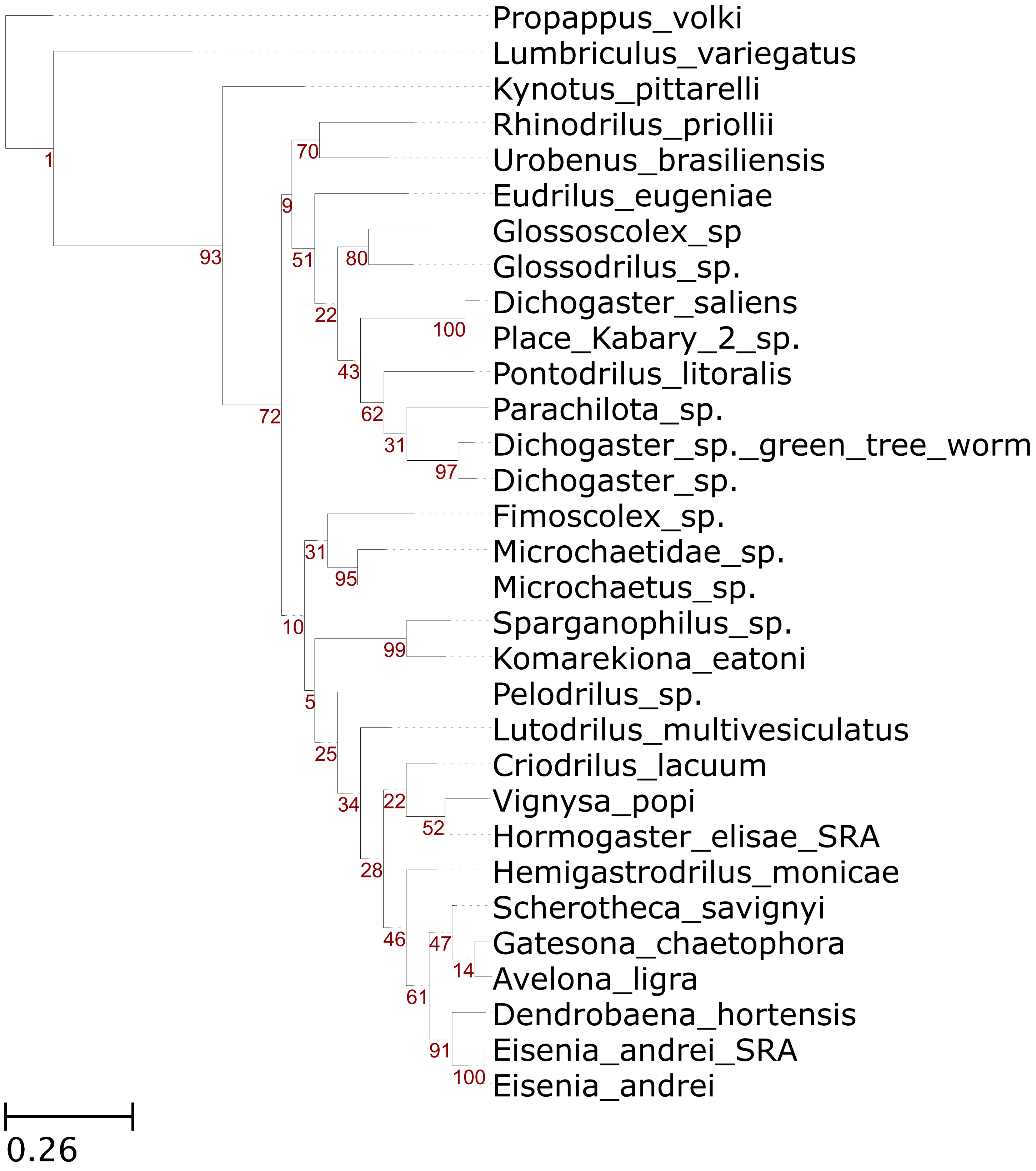

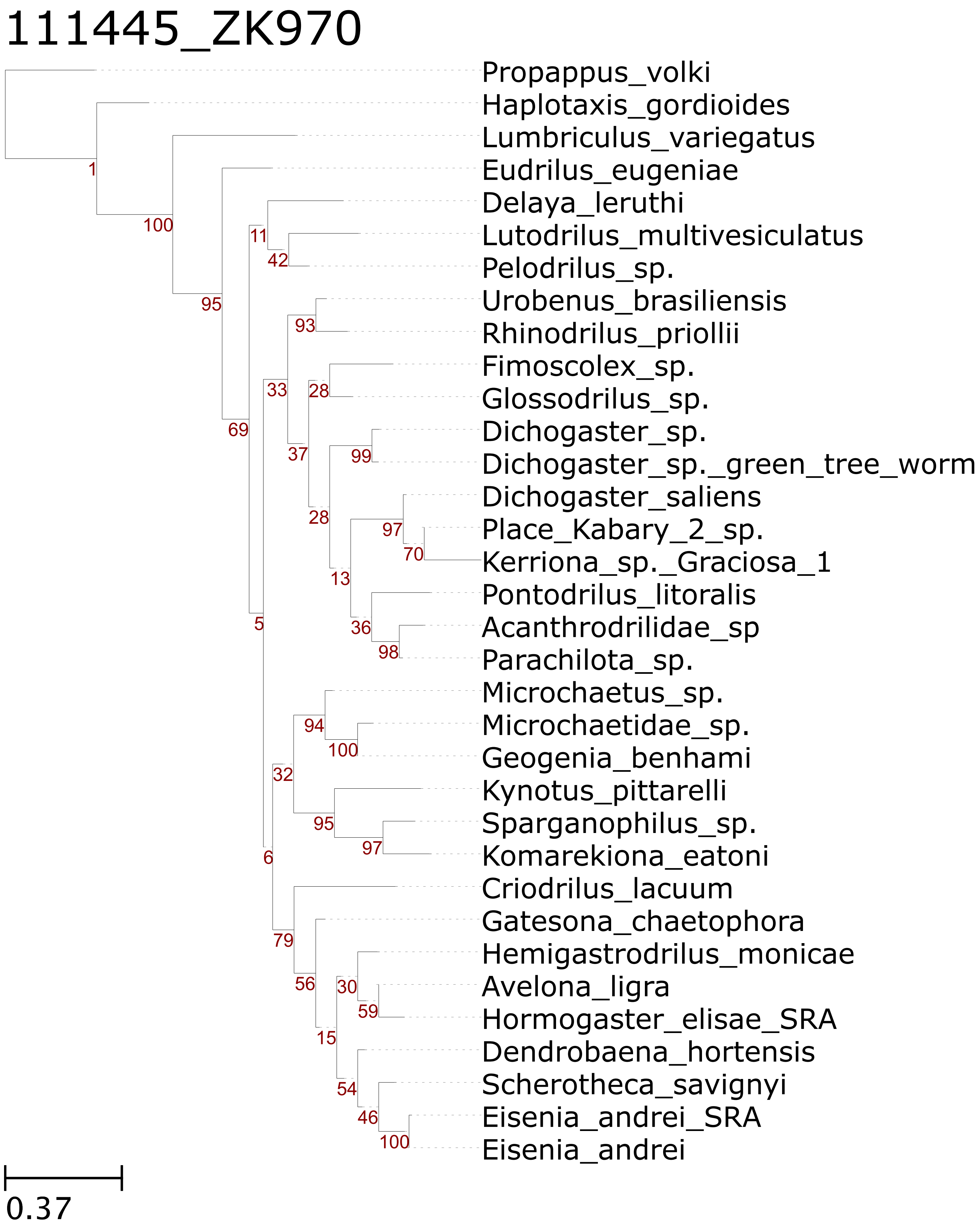

111445_ZK970
Propappus_volki
Haplotaxis_gordioides
Lumbriculus_variegatus
Eudrilus_eugeniae
Delaya_leruthi
Lutodrilus_multivesiculatus
Pelodrilus_sp.
Urobenus_brasiliensis
Rhinodrilus_priollii
Fimoscolex_sp.
Glossodrilus_sp.
Dichogaster_sp.
Dichogaster_sp._green_tree_worm
Dichogaster_saliens
Place_Kabary_2_sp.
Kerriona_sp._Graciosa_1
Pontodrilus_litoralis
Acanthrodrilidae_sp
Parachilota_sp.
Microchaetus_sp.
Microchaetidae_sp.
Geogenia_benhami
Kynotus_pittarelli
Sparganophilus_sp.
Komarekiona_eatoni
Criodrilus_lacuum
Gatesona_chaetophora
Hemigastrodrilus_monicae
Avelona_ligra
Hormogaster_elisae_SRA
Dendrobaena_hortensis
Scherotheca_savignyi
Eisenia_andrei_SRA
Eisenia_andrei
0.37

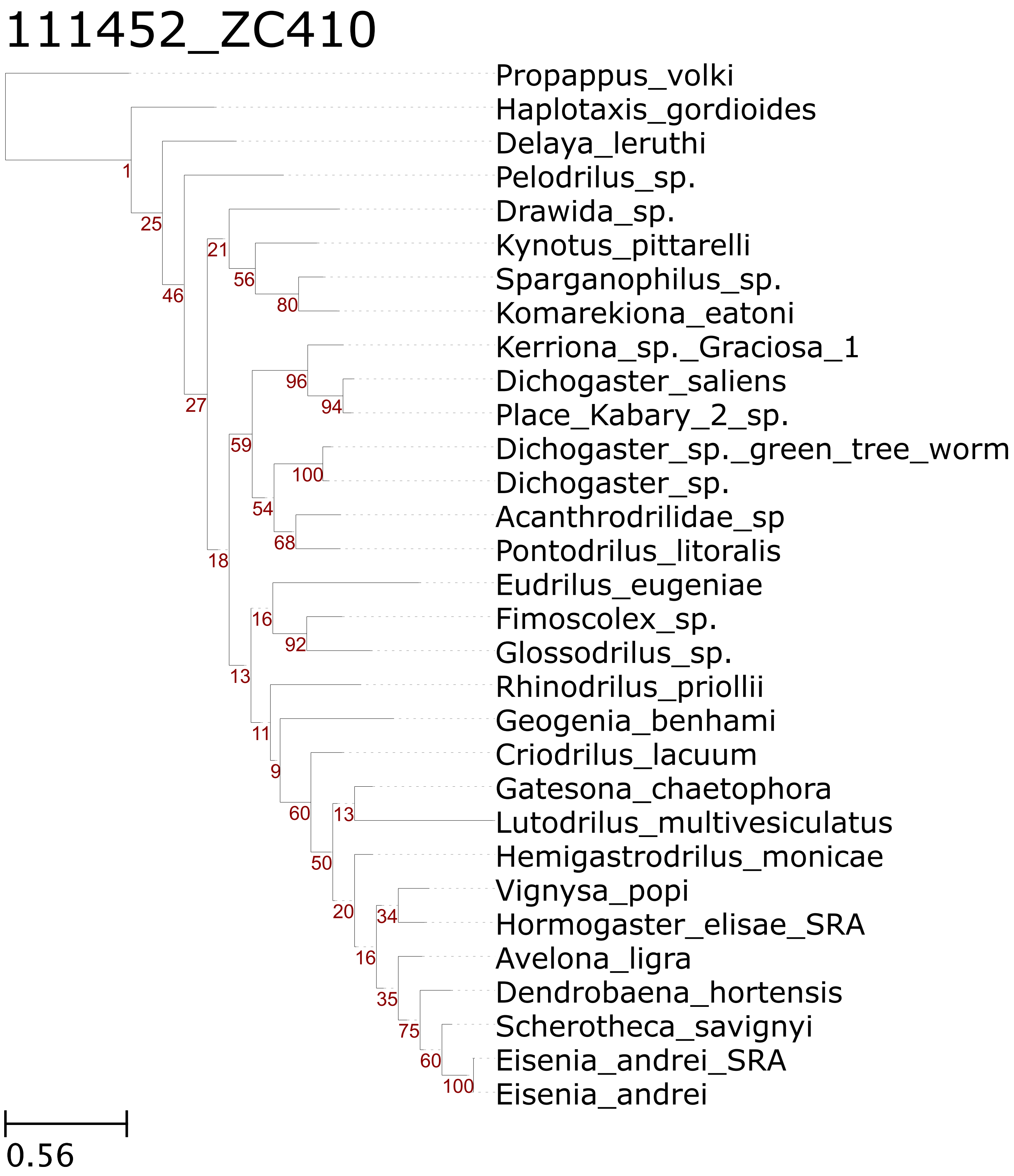
111452_ZC410
Propappus_volki
Haplotaxis_gordioides
Delaya_leruthi
Pelodrilus_sp.
Drawida_sp.
Kynotus_pittarelli
Sparganophilus_sp.
Komarekiona_eatoni
Kerriona_sp._Graciosa_1
Dichogaster_saliens
Place_Kabary_2_sp.
Dichogaster_sp._green_tree_worm
Dichogaster_sp.
Acanthrodrilidae_sp
Pontodrilus_litoralis
Eudrilus_eugeniae
Fimoscolex_sp.
Glossodrilus_sp.
Rhinodrilus_priollii
Geogenia_benhami
Criodrilus_lacuum
Gatesona_chaetophora
Lutodrilus_multivesiculatus
Hemigastrodrilus_monicae
Vignysa_popi
Hormogaster_elisae_SRA
Avelona_ligra
Dendrobaena_hortensis
Scherotheca_savignyi
Eisenia_andrei_SRA
Eisenia_andrei
1
25
46
27
21
56
80
96
94
59
100
54
68
18
16
92
13
11
9
60
13
50
20
34
16
35
75
60
100
0.56

111483_T10B11

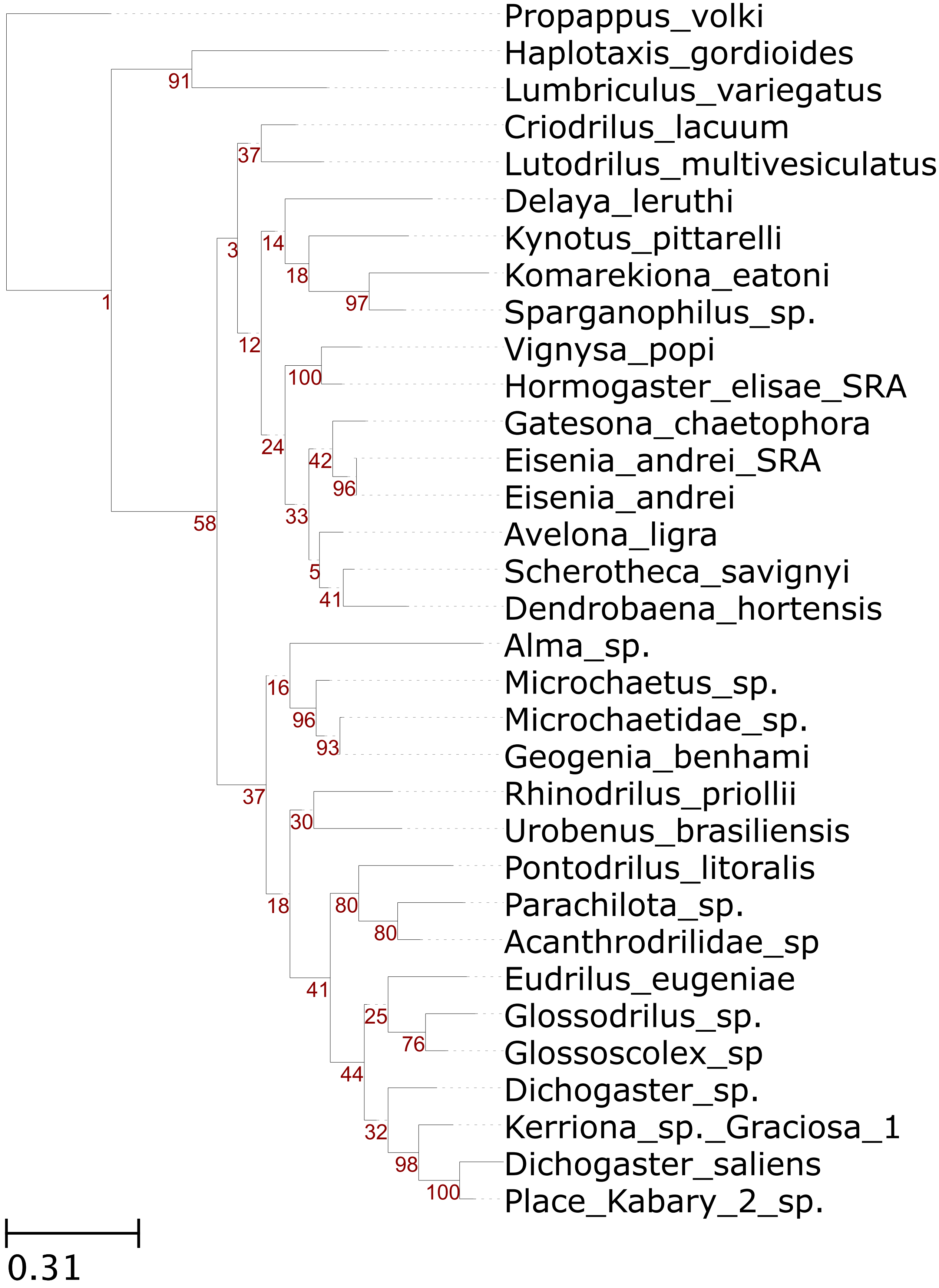

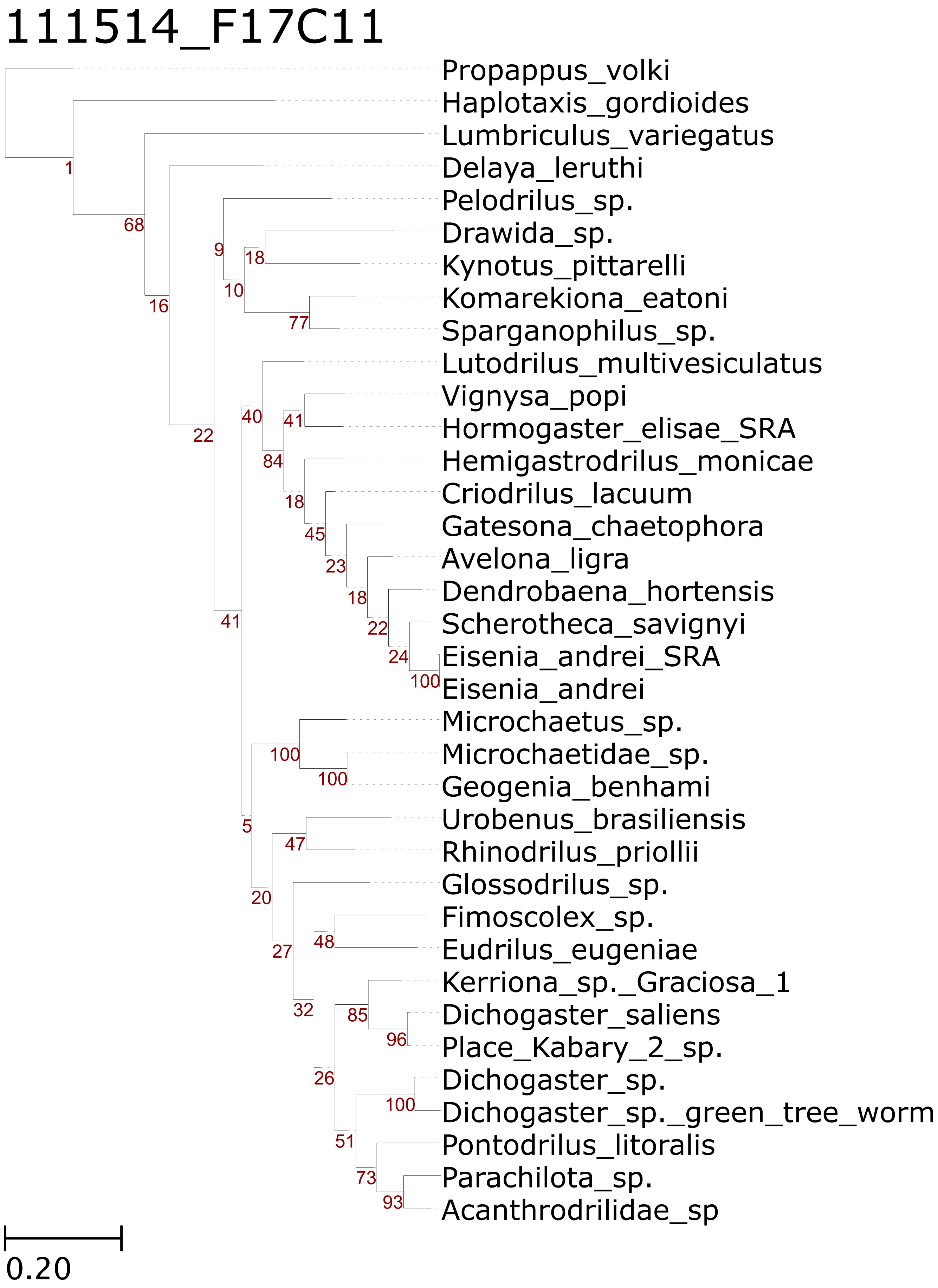
111514_F17C11
Propappus_volki
Haplotaxis_gordioides
Lumbriculus_variegatus
Delaya_leruthi
Pelodrilus_sp.
Drawida_sp.
Kynotus_pittarelli
Komarekiona_eatoni
Sparganophilus_sp.
Lutodrilus_multivesiculatus
Vignysa_popi
Hormogaster_elisae_SRA
Hemigastrodrilus_monicae
Criodrilus_lacuum
Gatesona_chaetophora
Avelona_ligra
Dendrobaena_hortensis
Scherotheca_savignyi
Eisenia_andrei_SRA
Eisenia_andrei
Microchaetus_sp.
Microchaetidae_sp.
Geogenia_benhami
Urobenus_brasiliensis
Rhinodrilus_priollii
Glossodrilus_sp.
Fimoscolex_sp.
Eudrilus_eugeniae
Kerriona_sp._Graciosa_1
Dichogaster_saliens
Place_Kabary_2_sp.
Dichogaster_sp.
Dichogaster_sp._green_tree_worm
Pontodrilus_litoralis
Parachilota_sp.
Acanthrodrilidae_sp
1
68
16
22
9
10
18
77
41
40
84
41
18
45
23
18
22
24
100
100
100
5
47
20
27
48
32
85
96
26
100
51
73
93
0.20

111608_C15F1

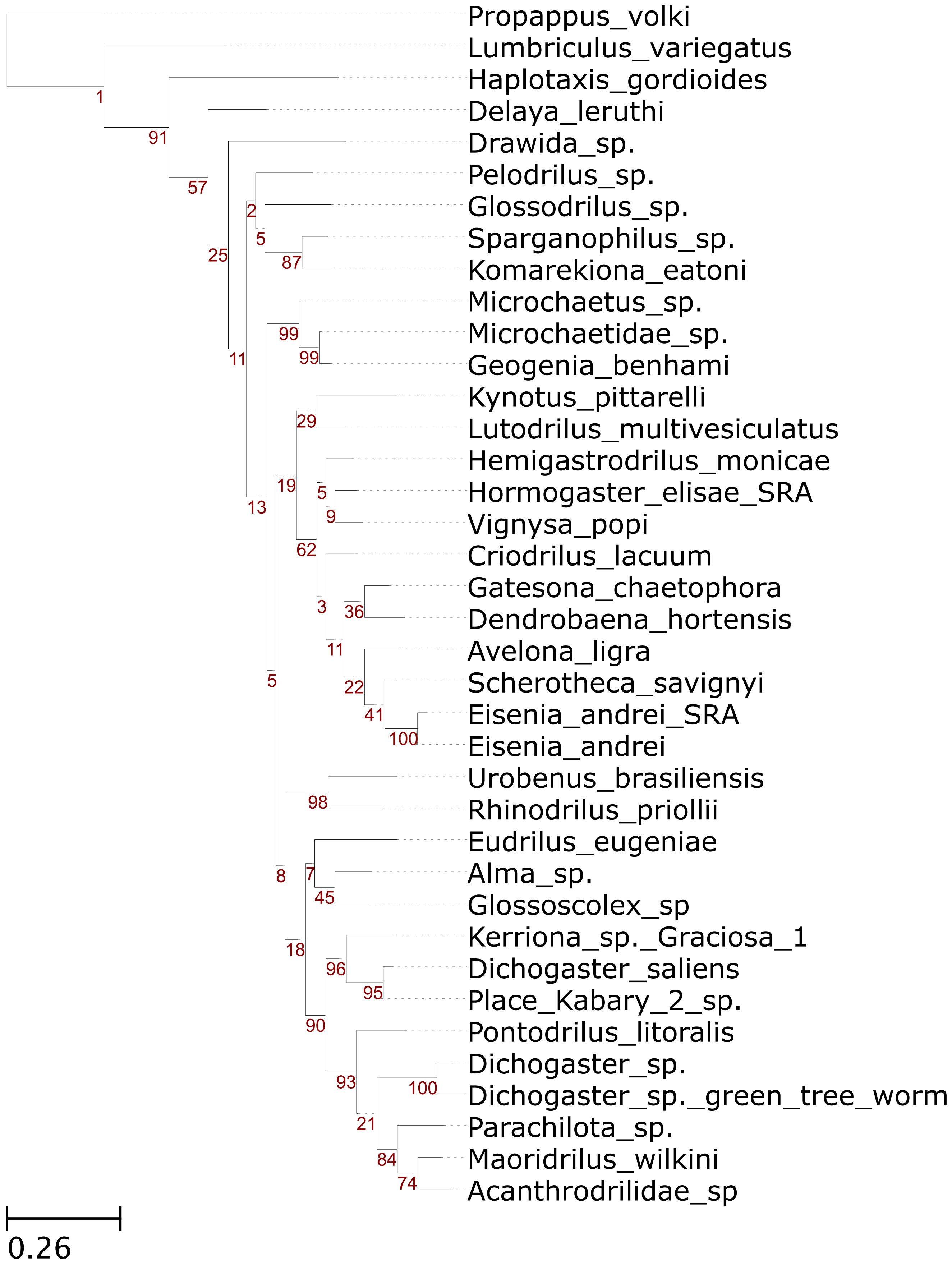

111614_T22C1

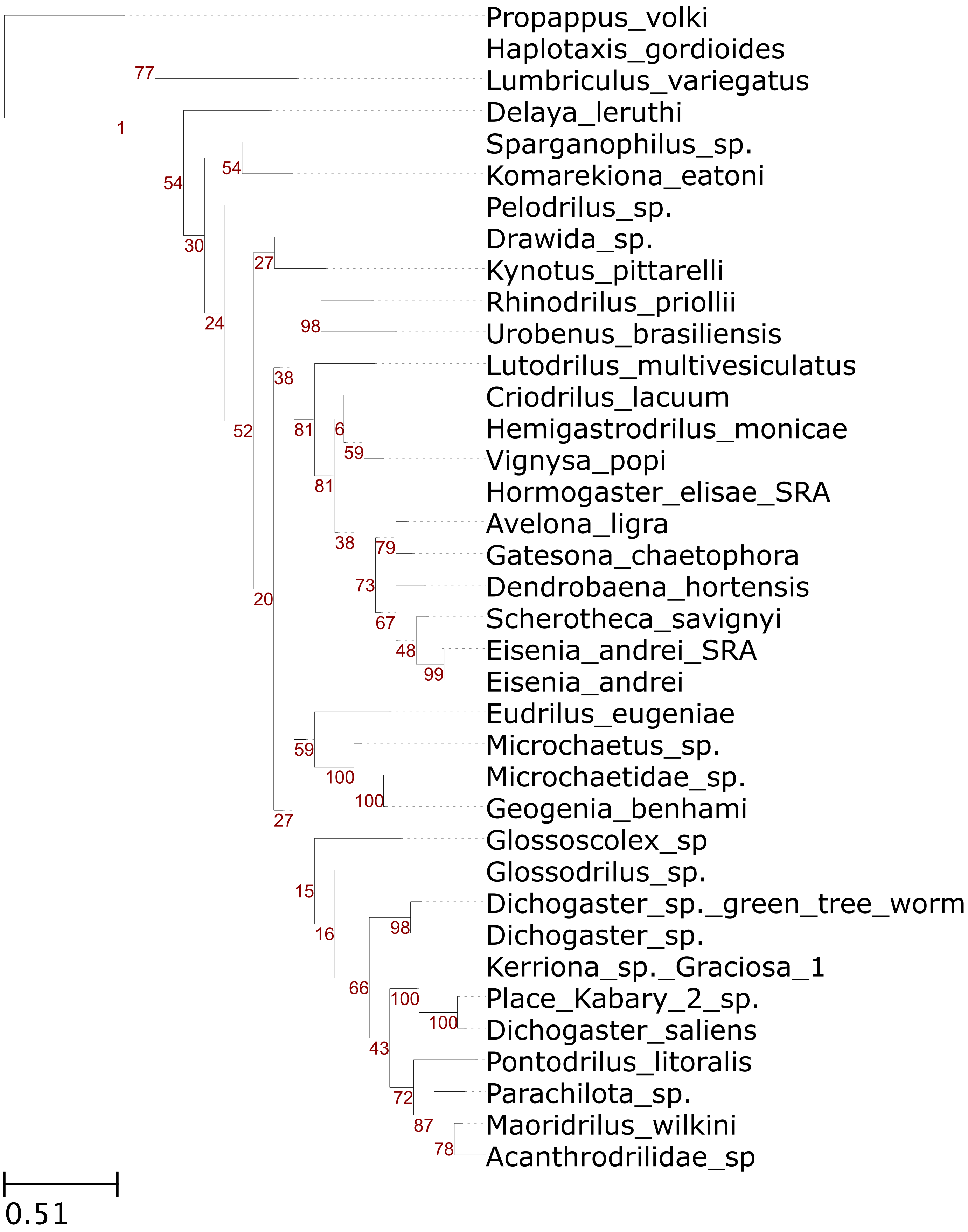

111623_F10D11

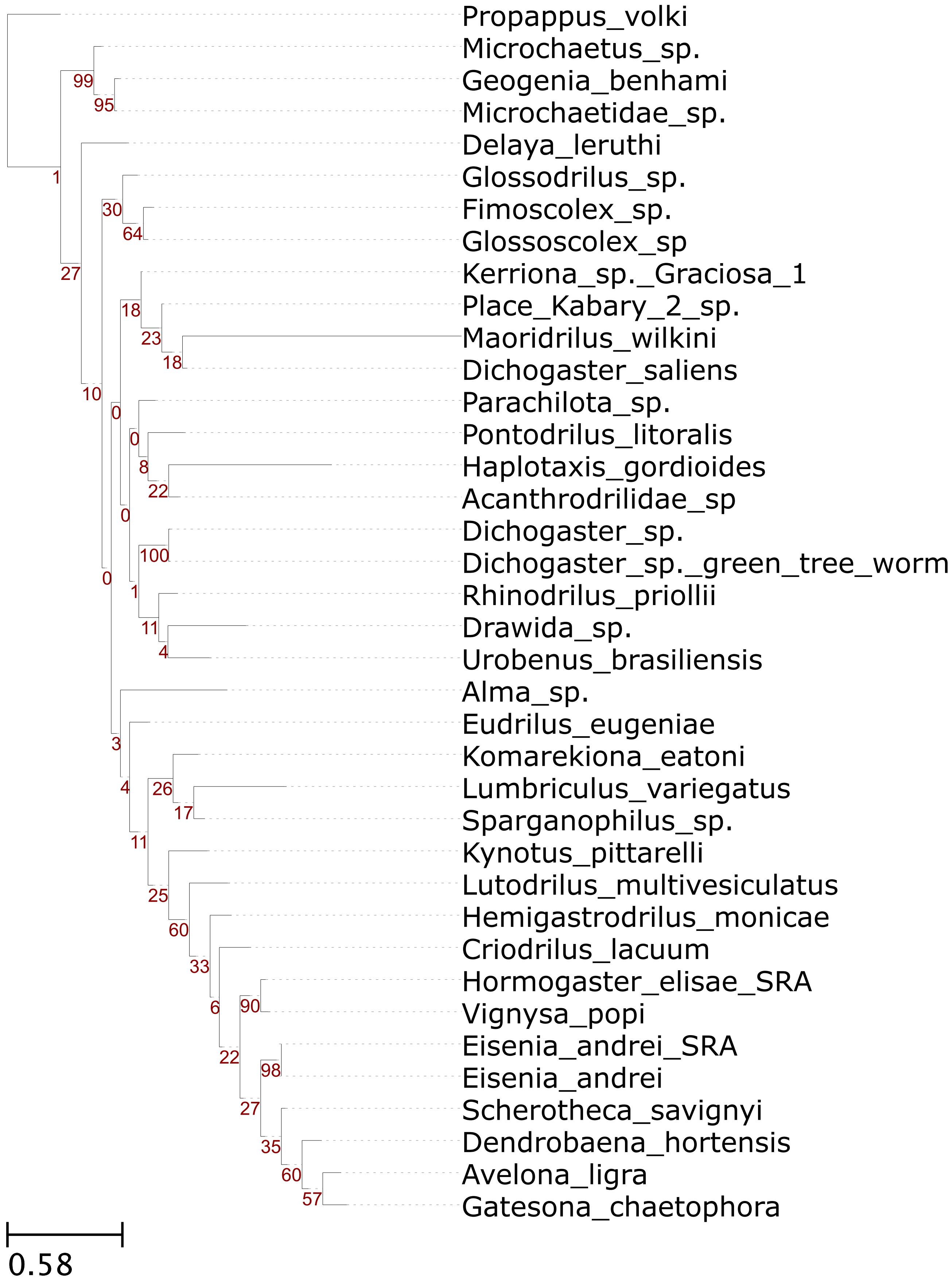

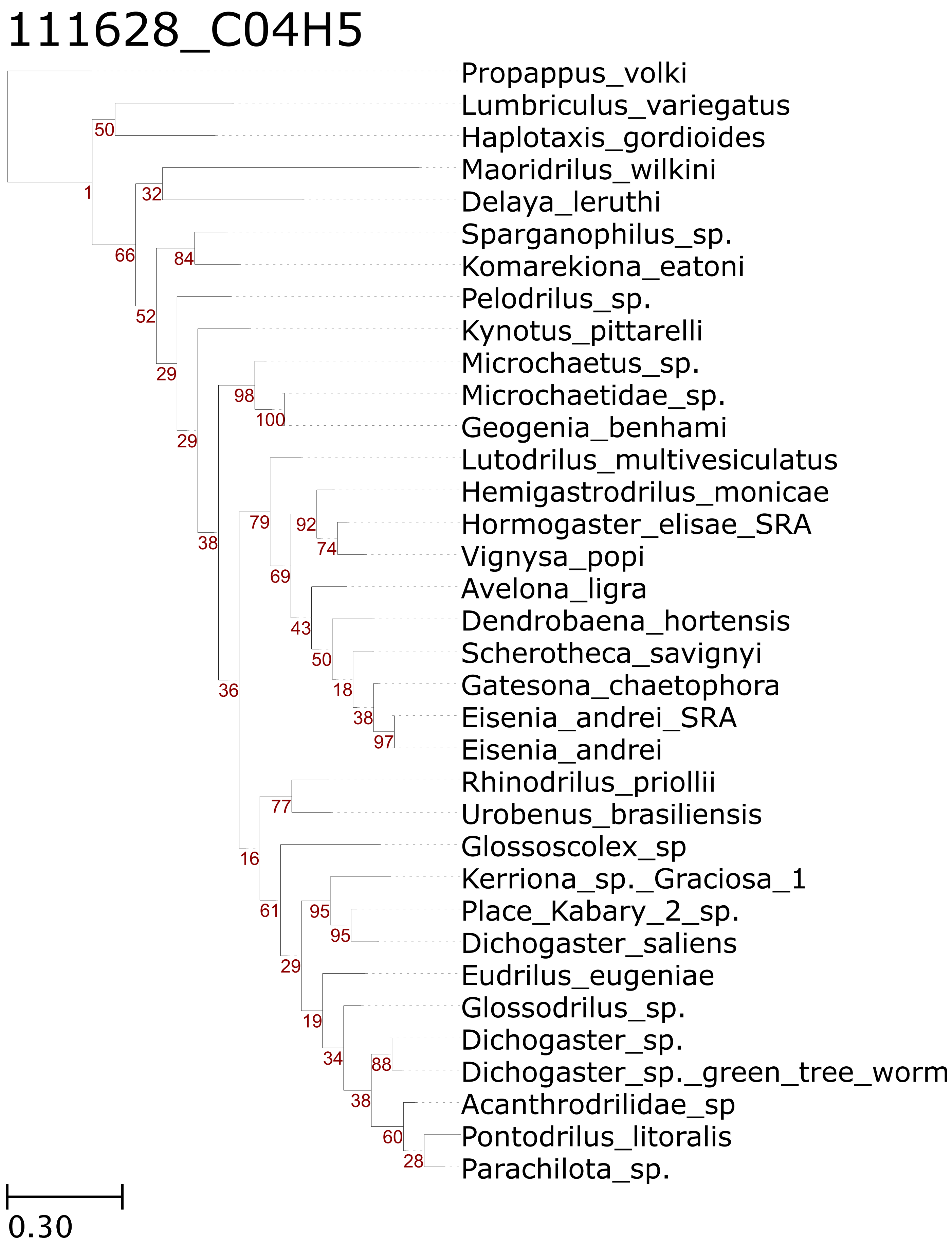
111628_C04H5
Propappus_volki
Lumbriculus_variegatus
Haplotaxis_gordioides
Maoridrilus_wilkini
Delaya_leruthi
Sparganophilus_sp.
Komarekiona_eatoni
Pelodrilus_sp.
Kynotus_pittarelli
Microchaetus_sp.
Microchaetidae_sp.
Geogenia_benhami
Lutodrilus_multivesiculatus
Hemigastrodrilus_monicae
Hormogaster_elisae_SRA
Vignysa_popi
Avelona_ligra
Dendrobaena_hortensis
Scherotheca_savignyi
Gatesona_chaetophora
Eisenia_andrei_SRA
Eisenia_andrei
Rhinodrilus_priollii
Urobenus_brasiliensis
Glossoscolex_sp
Kerriona_sp._Graciosa_1
Place_Kabary_2_sp.
Dichogaster_saliens
Eudrilus_eugeniae
Glossodrilus_sp.
Dichogaster_sp.
Dichogaster_sp._green_tree_worm
Acanthrodrilidae_sp
Pontodrilus_litoralis
Parachilota_sp.
50
1
32
66
84
52
29
98
100
29
38
79
92
74
69
43
50
18
38
97
36
77
16
61
95
95
29
19
34
88
38
60
28
0.30

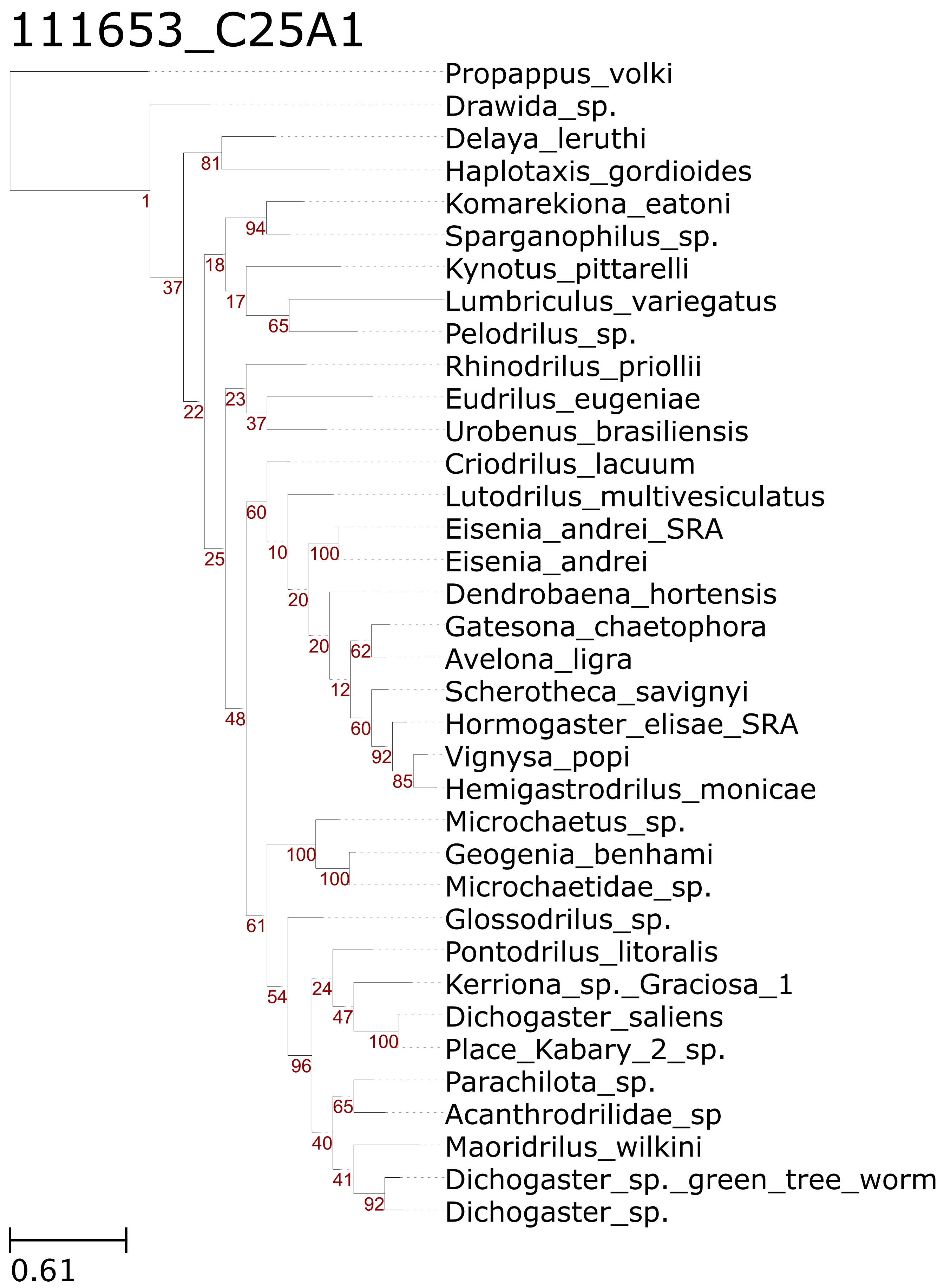
111653_C25A1
Propappus_volki
Drawida_sp.
Delaya_leruthi
Haplotaxis_gordioides
Komarekiona_eatoni
Sparganophilus_sp.
Kynotus_pittarelli
Lumbriculus_variegatus
Pelodrilus_sp.
Rhinodrilus_priollii
Eudrilus_eugeniae
Urobenus_brasiliensis
Criodrilus_lacuum
Lutodrilus_multivesiculatus
Eisenia_andrei_SRA
Eisenia_andrei
Dendrobaena_hortensis
Gatesona_chaetophora
Avelona_ligra
Scherotheca_savignyi
Hormogaster_elisae_SRA
Vignysa_popi
Hemigastrodrilus_monicae
Microchaetus_sp.
Geogenia_benhami
Microchaetidae_sp.
Glossodrilus_sp.
Pontodrilus_litoralis
Kerriona_sp._Graciosa_1
Dichogaster_saliens
Place_Kabary_2_sp.
Parachilota_sp.
Acanthrodrilidae_sp
Maoridrilus_wilkini
Dichogaster_sp._green_tree_worm
Dichogaster_sp.
0.61

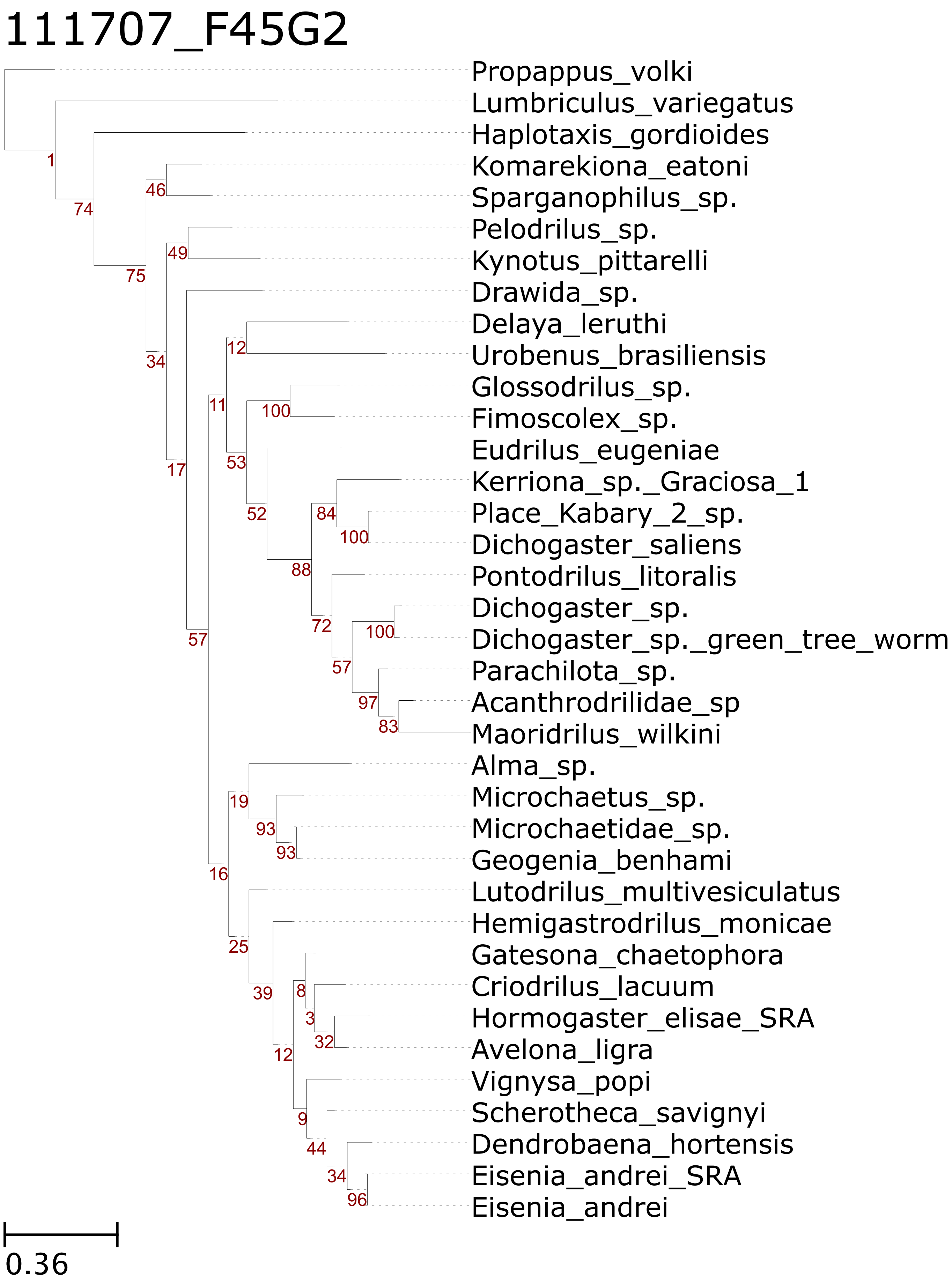
111707_F45G2
Propappus_volki
Lumbriculus_variegatus
Haplotaxis_gordioides
Komarekiona_eatoni
Sparganophilus_sp.
Pelodrilus_sp.
Kynotus_pittarelli
Drawida_sp.
Delaya_leruthi
Urobenus_brasiliensis
Glossodrilus_sp.
Fimoscolex_sp.
Eudrilus_eugeniae
Kerriona_sp._Graciosa_1
Place_Kabary_2_sp.
Dichogaster_saliens
Pontodrilus_litoralis
Dichogaster_sp.
Dichogaster_sp._green_tree_worm
Parachilota_sp.
Acanthrodrilidae_sp
Maoridrilus_wilkini
Alma_sp.
Microchaetus_sp.
Microchaetidae_sp.
Geogenia_benhami
Lutodrilus_multivesiculatus
Hemigastrodrilus_monicae
Gatesona_chaetophora
Criodrilus_lacuum
Hormogaster_elisae_SRA
Avelona_ligra
Vignysa_popi
Scherotheca_savignyi
Dendrobaena_hortensis
Eisenia_andrei_SRA
Eisenia_andrei
1
74
75
46
49
34
17
57
12
11
53
100
52
88
84
100
72
57
100
97
83
16
19
93
93
25
39
12
8
3
32
9
44
34
96
0.36

111731_F39H11

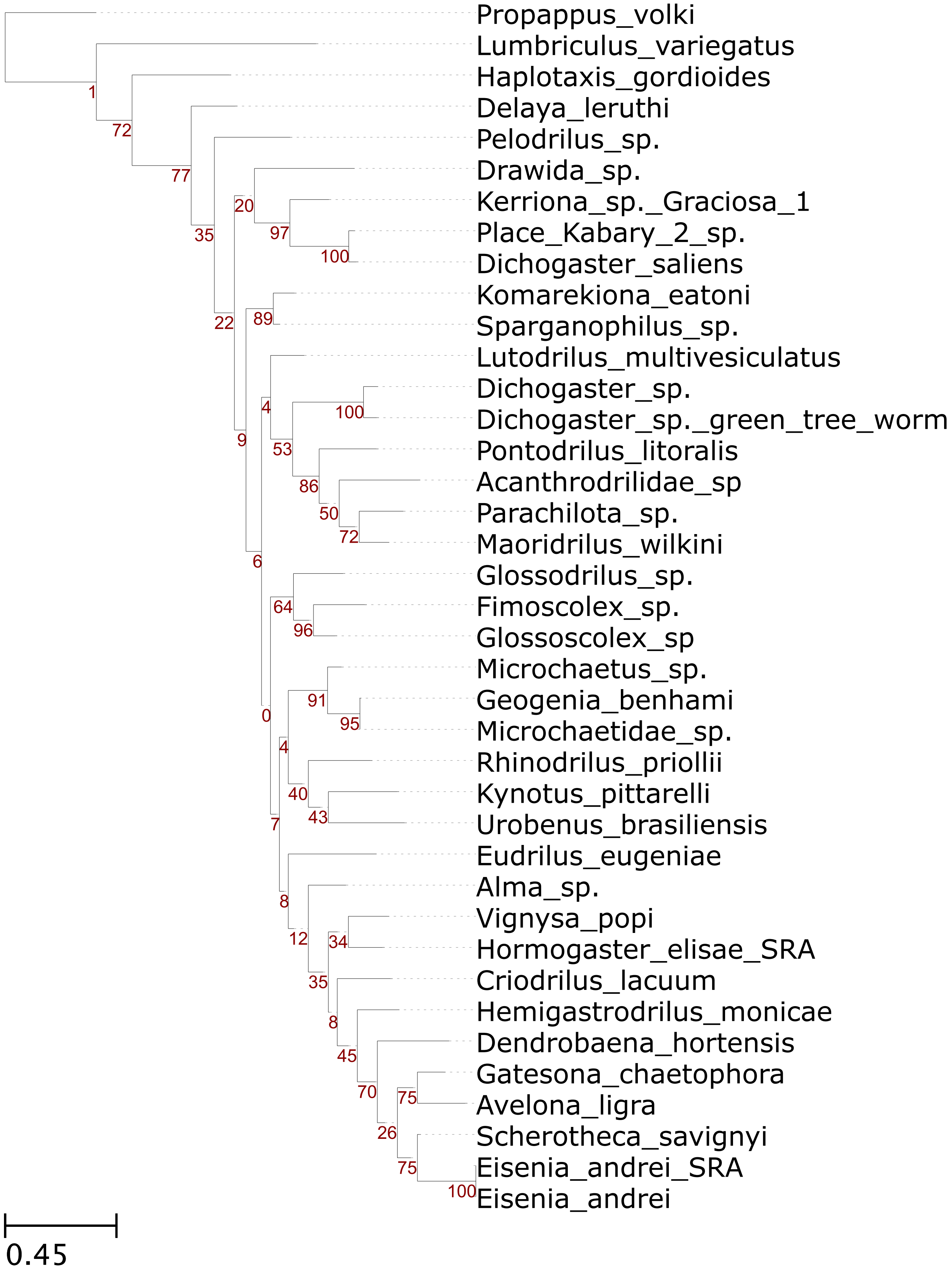

111734_T25B9

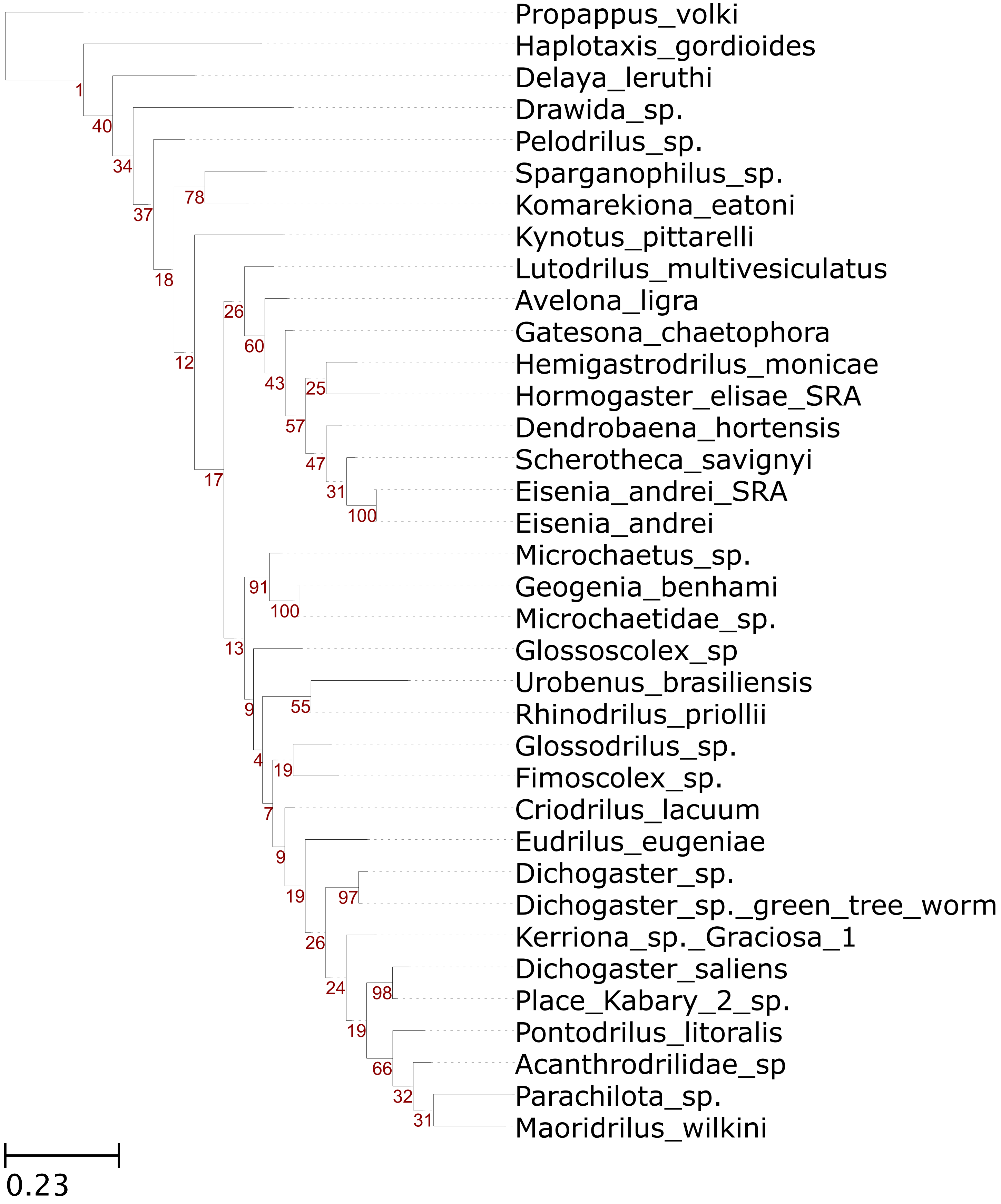

111739_M01B12

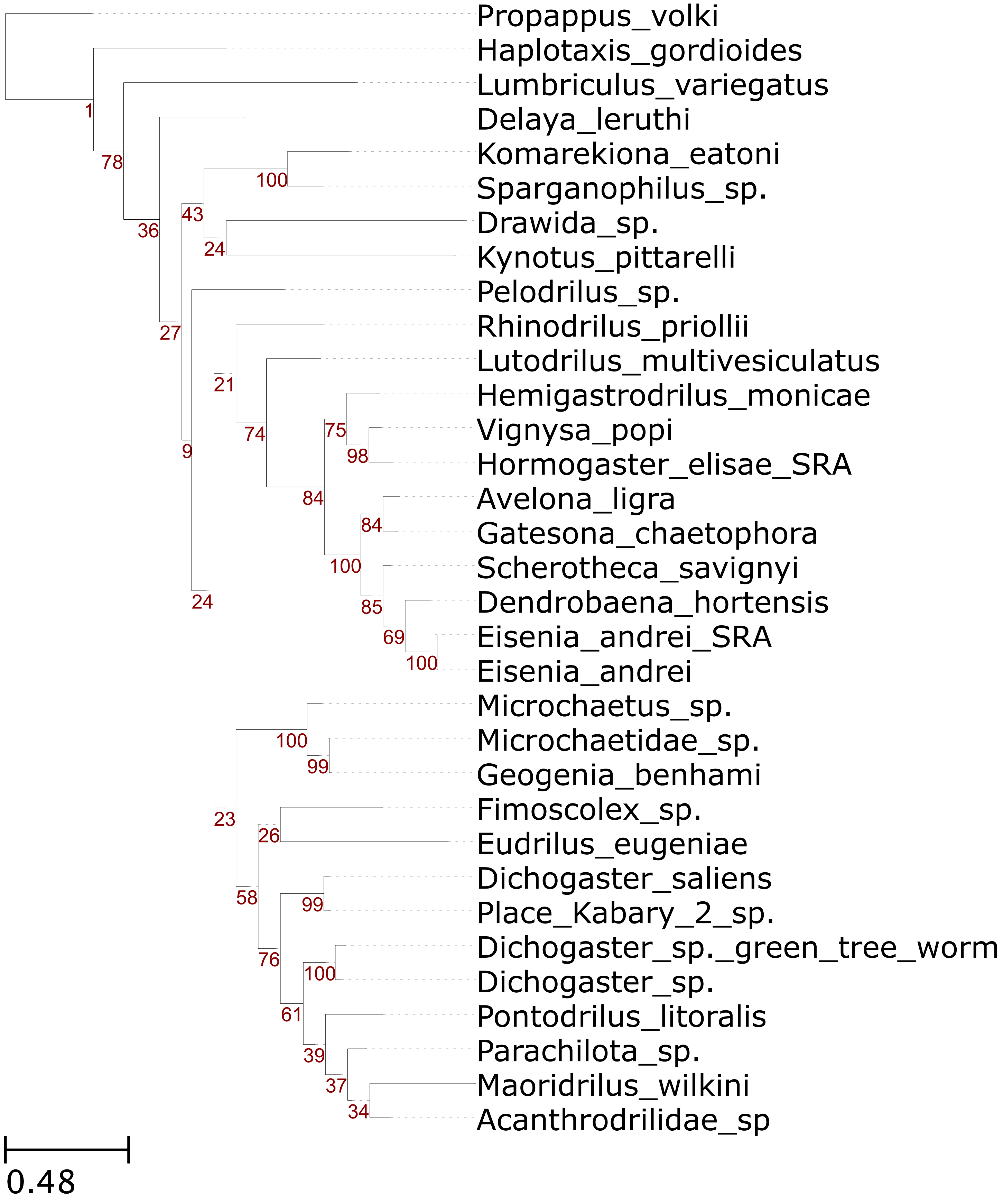

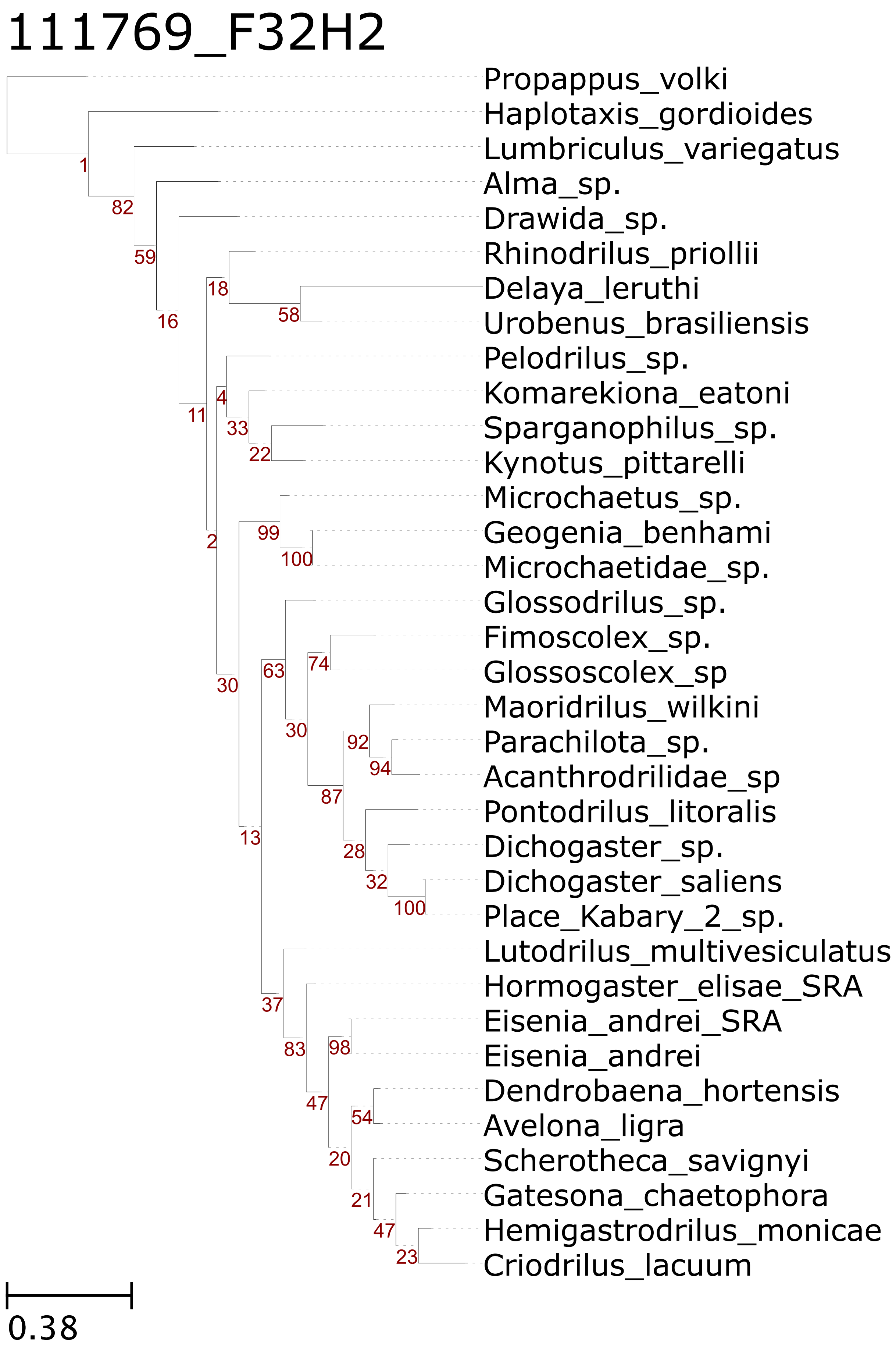

111769_F32H2
Propappus_volki
Haplotaxis_gordioides
Lumbriculus_variegatus
Alma_sp.
Drawida_sp.
Rhinodrilus_priollii
Delaya_leruthi
Urobenus_brasiliensis
Pelodrilus_sp.
Komarekiona_eatoni
Sparganophilus_sp.
Kynotus_pittarelli
Microchaetus_sp.
Geogenia_benhami
Microchaetidae_sp.
Glossodrilus_sp.
Fimoscolex_sp.
Glossoscolex_sp
Maoridrilus_wilkini
Parachilota_sp.
Acanthrodrilidae_sp
Pontodrilus_litoralis
Dichogaster_sp.
Dichogaster_saliens
Place_Kabary_2_sp.
Lutodrilus_multivesiculatus
Hormogaster_elisae_SRA
Eisenia_andrei_SRA
Eisenia_andrei
Dendrobaena_hortensis
Avelona_ligra
Scherotheca_savignyi
Gatesona_chaetophora
Hemigastrodrilus_monicae
Criodrilus_lacuum
1
82
59
16
18
58
11
4
33
22
2
99
100
30
63
74
30
92
94
87
13
28
32
100
37
83
98
47
54
20
21
47
23
0.38

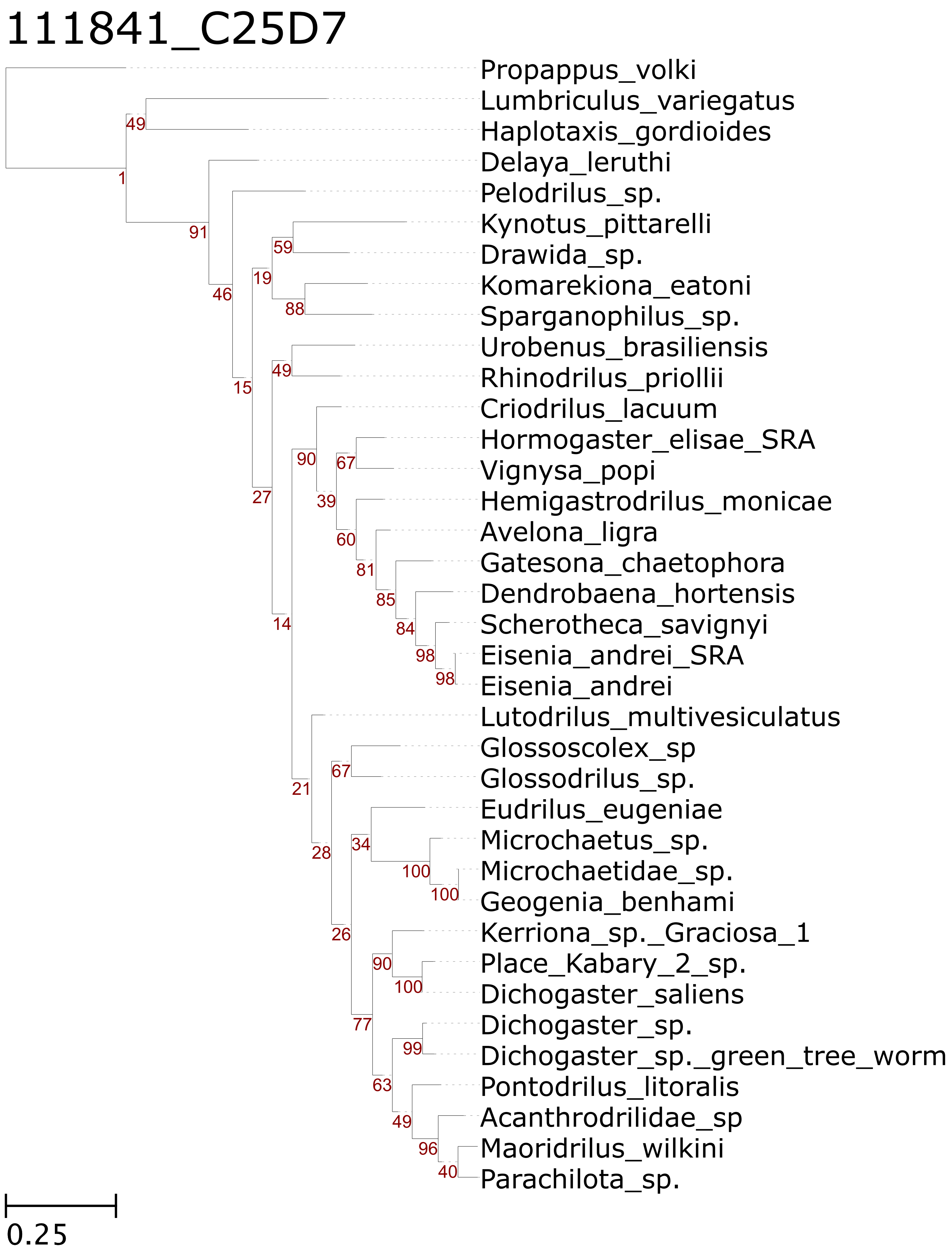
111841_C25D7
Propappus_volki
Lumbriculus_variegatus
Haplotaxis_gordioides
Delaya_leruthi
Pelodrilus_sp.
Kynotus_pittarelli
Drawida_sp.
Komarekiona_eatoni
Sparganophilus_sp.
Urobenus_brasiliensis
Rhinodrilus_priollii
Criodrilus_lacuum
Hormogaster_elisae_SRA
Vignysa_popi
Hemigastrodrilus_monicae
Avelona_ligra
Gatesona_chaetophora
Dendrobaena_hortensis
Scherotheca_savignyi
Eisenia_andrei_SRA
Eisenia_andrei
Lutodrilus_multivesiculatus
Glossoscolex_sp
Glossodrilus_sp.
Eudrilus_eugeniae
Microchaetus_sp.
Microchaetidae_sp.
Geogenia_benhami
Kerriona_sp._Graciosa_1
Place_Kabary_2_sp.
Dichogaster_saliens
Dichogaster_sp.
Dichogaster_sp._green_tree_worm
Pontodrilus_litoralis
Acanthrodrilidae_sp
Maoridrilus_wilkini
Parachilota_sp.
0.25

111870_R07E5

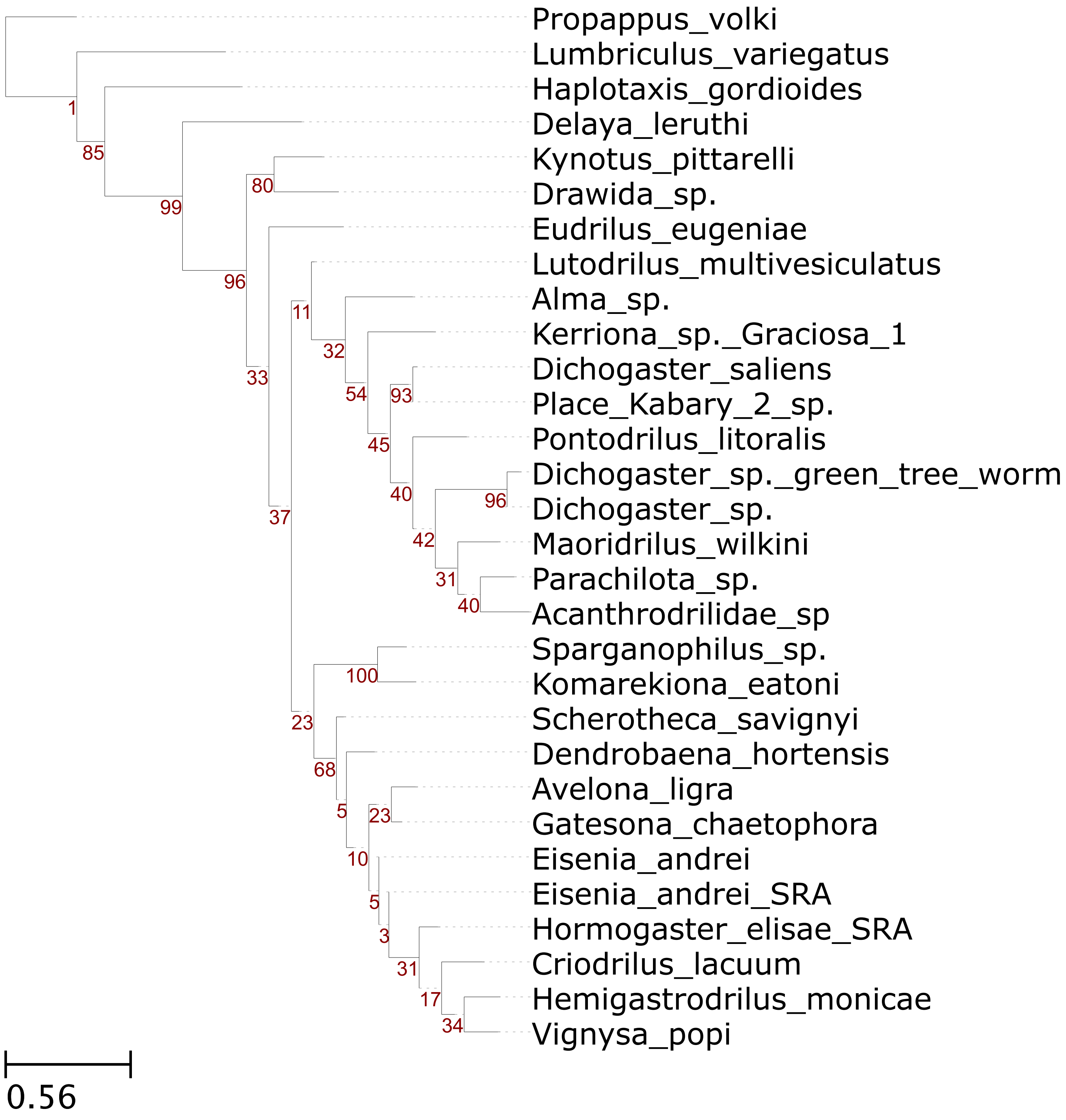

111900_Y55F3AM

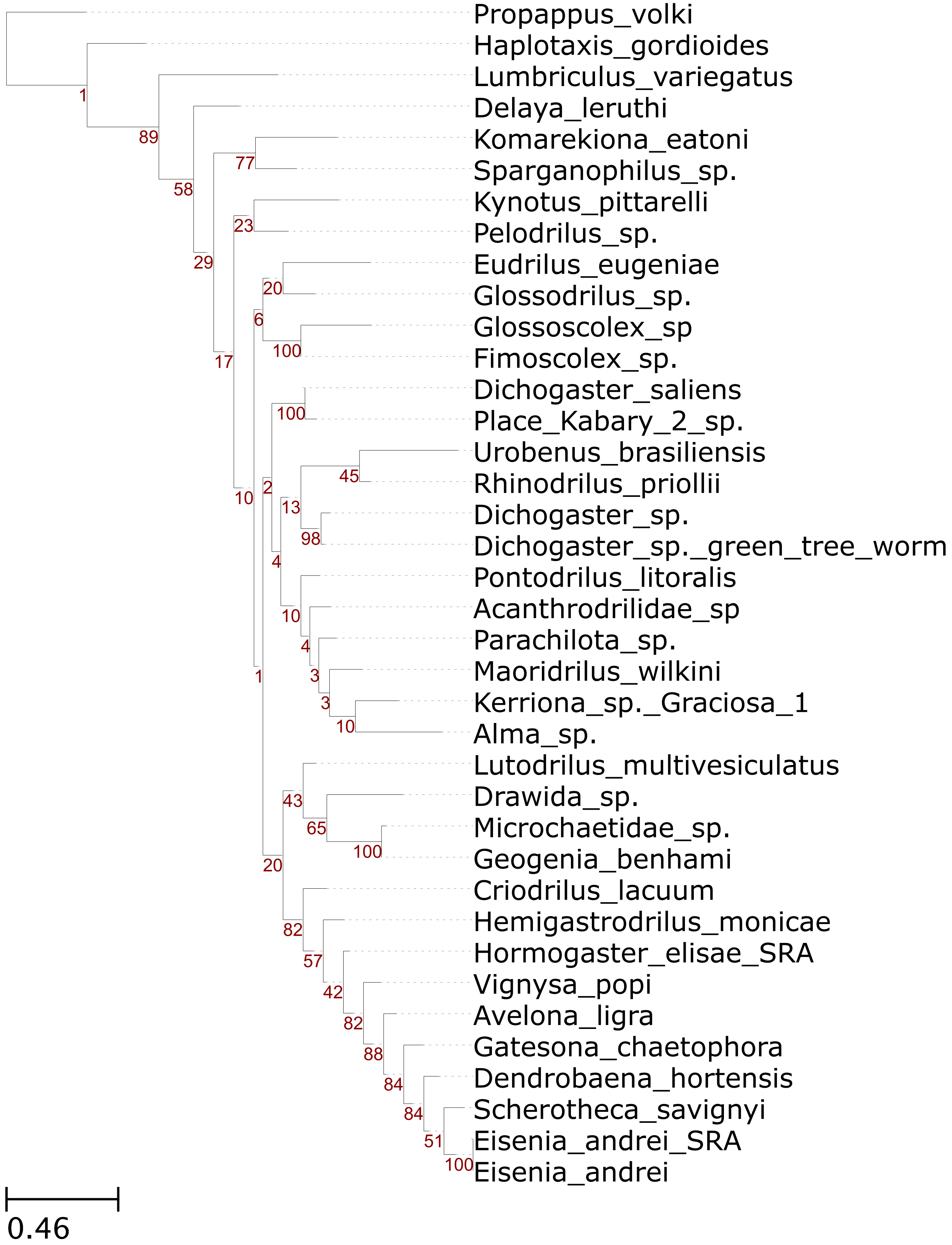

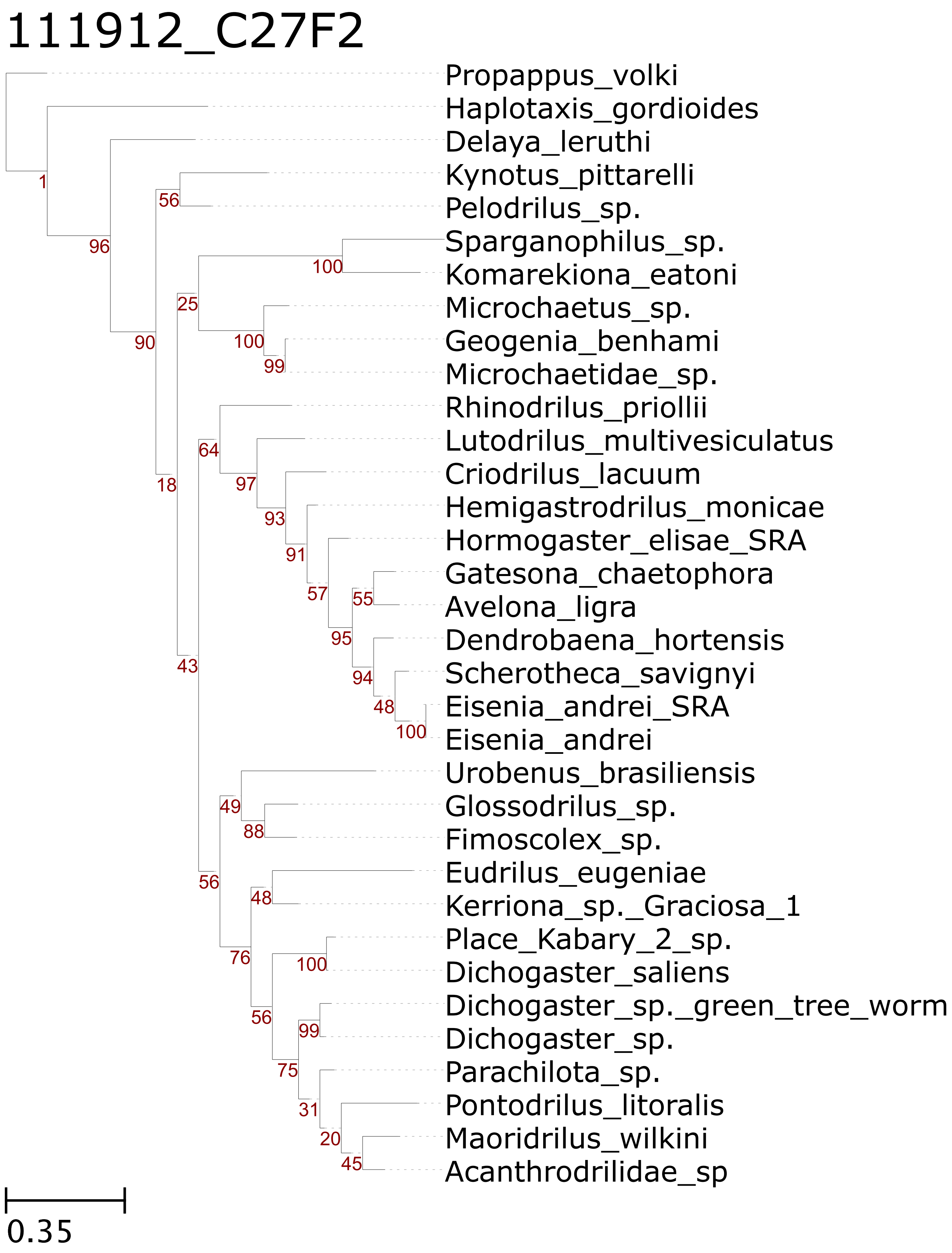
111912_C27F2
Propappus_volki
Haplotaxis_gordioides
Delaya_leruthi
Kynotus_pittarelli
Pelodrilus_sp.
Sparganophilus_sp.
Komarekiona_eatoni
Microchaetus_sp.
Geogenia_benhami
Microchaetidae_sp.
Rhinodrilus_priollii
Lutodrilus_multivesiculatus
Criodrilus_lacuum
Hemigastrodrilus_monicae
Hormogaster_elisae_SRA
Gatesona_chaetophora
Avelona_ligra
Dendrobaena_hortensis
Scherotheca_savignyi
Eisenia_andrei_SRA
Eisenia_andrei
Urobenus_brasiliensis
Glossodrilus_sp.
Fimoscolex_sp.
Eudrilus_eugeniae
Kerriona_sp._Graciosa_1
Place_Kabary_2_sp.
Dichogaster_saliens
Dichogaster_sp._green_tree_worm
Dichogaster_sp.
Parachilota_sp.
Pontodrilus_litoralis
Maoridrilus_wilkini
Acanthrodrilidae_sp
1
96
56
90
100
25
100
99
18
64
97
93
91
57
55
95
94
48
100
43
49
88
56
48
76
100
56
99
75
31
20
45
0.35

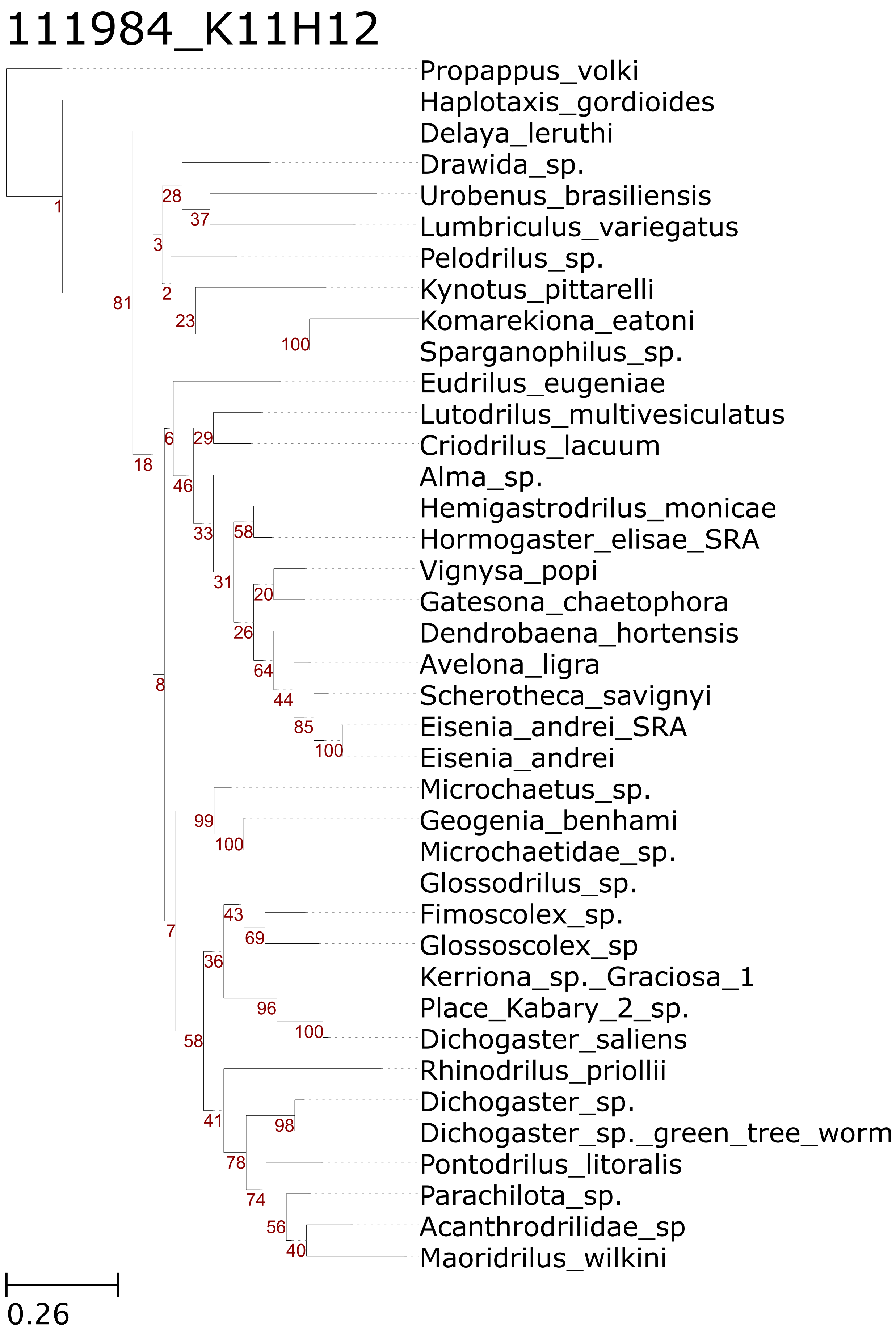
111984_K11H12
Propappus_volki
Haplotaxis_gordioides
Delaya_leruthi
Drawida_sp.
Urobenus_brasiliensis
Lumbriculus_variegatus
Pelodrilus_sp.
Kynotus_pittarelli
Komarekiona_eatoni
Sparganophilus_sp.
Eudrilus_eugeniae
Lutodrilus_multivesiculatus
Criodrilus_lacuum
Alma_sp.
Hemigastrodrilus_monicae
Hormogaster_elisae_SRA
Vignysa_popi
Gatesona_chaetophora
Dendrobaena_hortensis
Avelona_ligra
Scherotheca_savignyi
Eisenia_andrei_SRA
Eisenia_andrei
Microchaetus_sp.
Geogenia_benhami
Microchaetidae_sp.
Glossodrilus_sp.
Fimoscolex_sp.
Glossoscolex_sp
Kerriona_sp._Graciosa_1
Place_Kabary_2_sp.
Dichogaster_saliens
Rhinodrilus_priollii
Dichogaster_sp.
Dichogaster_sp._green_tree_worm
Pontodrilus_litoralis
Parachilota_sp.
Acanthrodrilidae_sp
Maoridrilus_wilkini
1
81
28
37
3
2
23
100
18
6
29
46
33
58
31
20
26
64
44
85
100
8
99
100
7
43
69
36
96
100
58
41
98
78
74
56
40
0.26

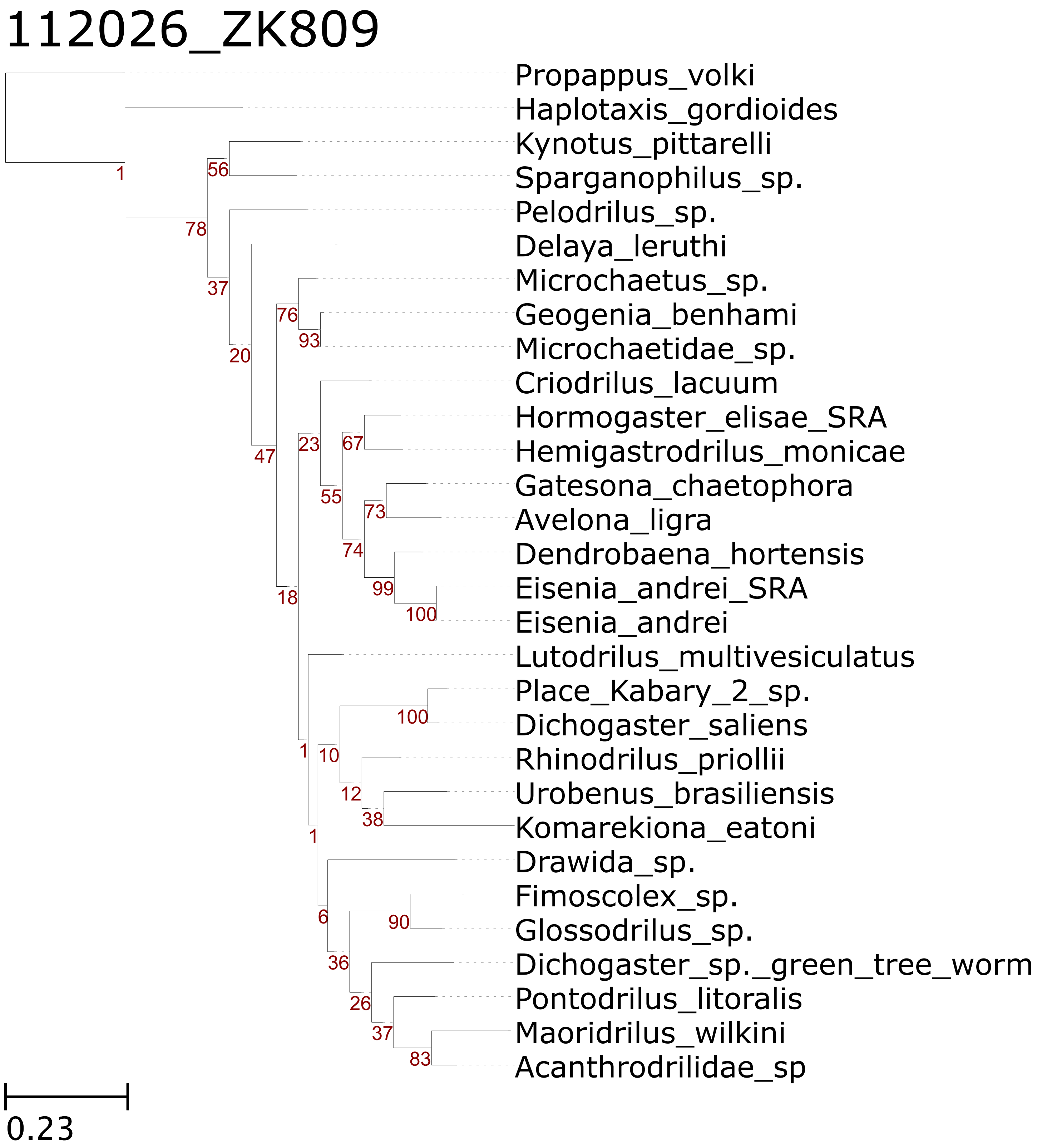
112026_ZK809
Propappus_volki
Haplotaxis_gordioides
Kynotus_pittarelli
Sparganophilus_sp.
Pelodrilus_sp.
Delaya_leruthi
Microchaetus_sp.
Geogenia_benhami
Microchaetidae_sp.
Criodrilus_lacuum
Hormogaster_elisae_SRA
Hemigastrodrilus_monicae
Gatesona_chaetophora
Avelona_ligra
Dendrobaena_hortensis
Eisenia_andrei_SRA
Eisenia_andrei
Lutodrilus_multivesiculatus
Place_Kabary_2_sp.
Dichogaster_saliens
Rhinodrilus_priollii
Urobenus_brasiliensis
Komarekiona_eatoni
Drawida_sp.
Fimoscolex_sp.
Glossodrilus_sp.
Dichogaster_sp._green_tree_worm
Pontodrilus_litoralis
Maoridrilus_wilkini
Acanthrodrilidae_sp
1
56
78
37
20
47
76
93
18
23
67
55
74
73
99
100
1
100
10
12
38
1
6
90
36
26
37
83
0.23

112048_F54C4

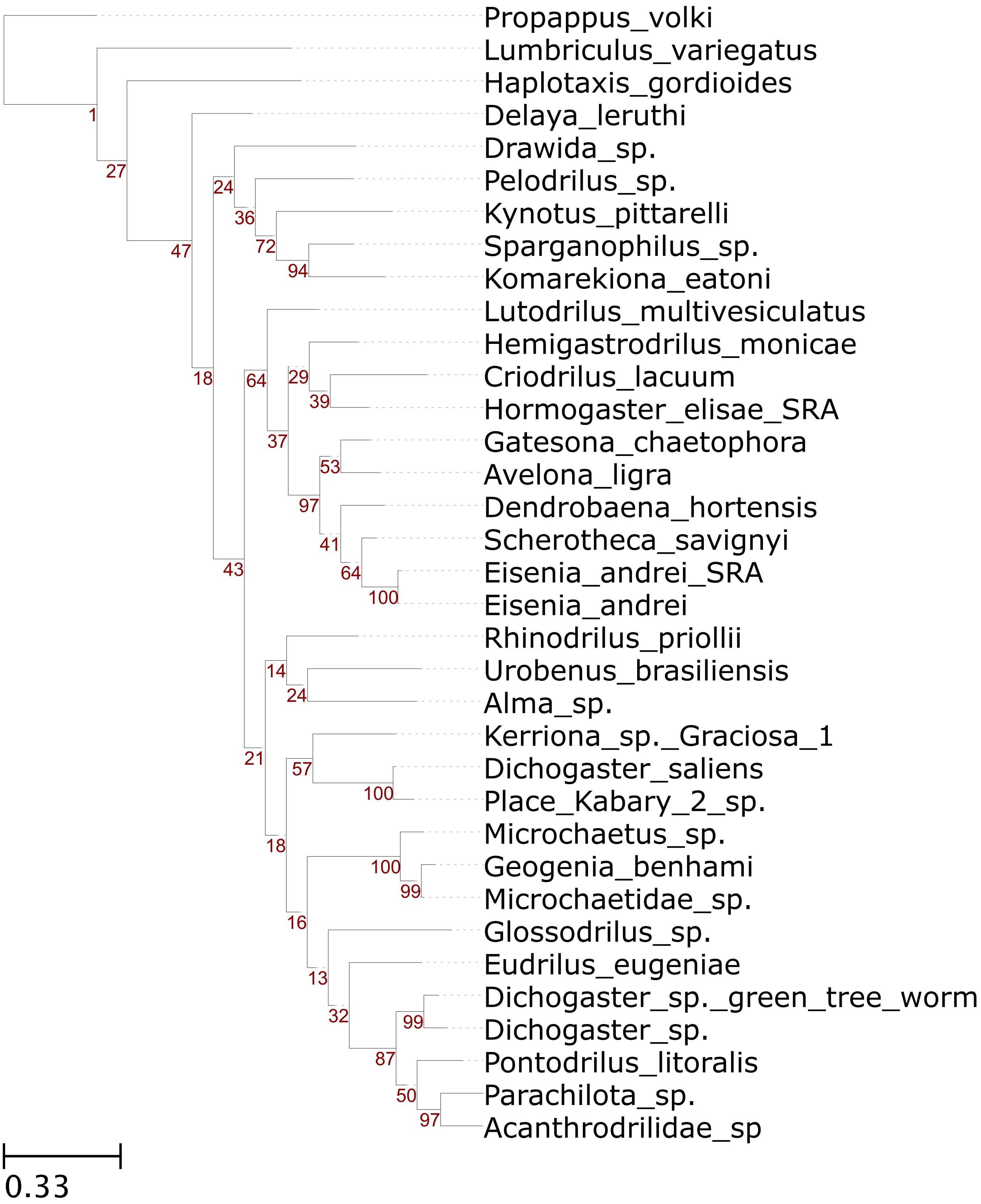

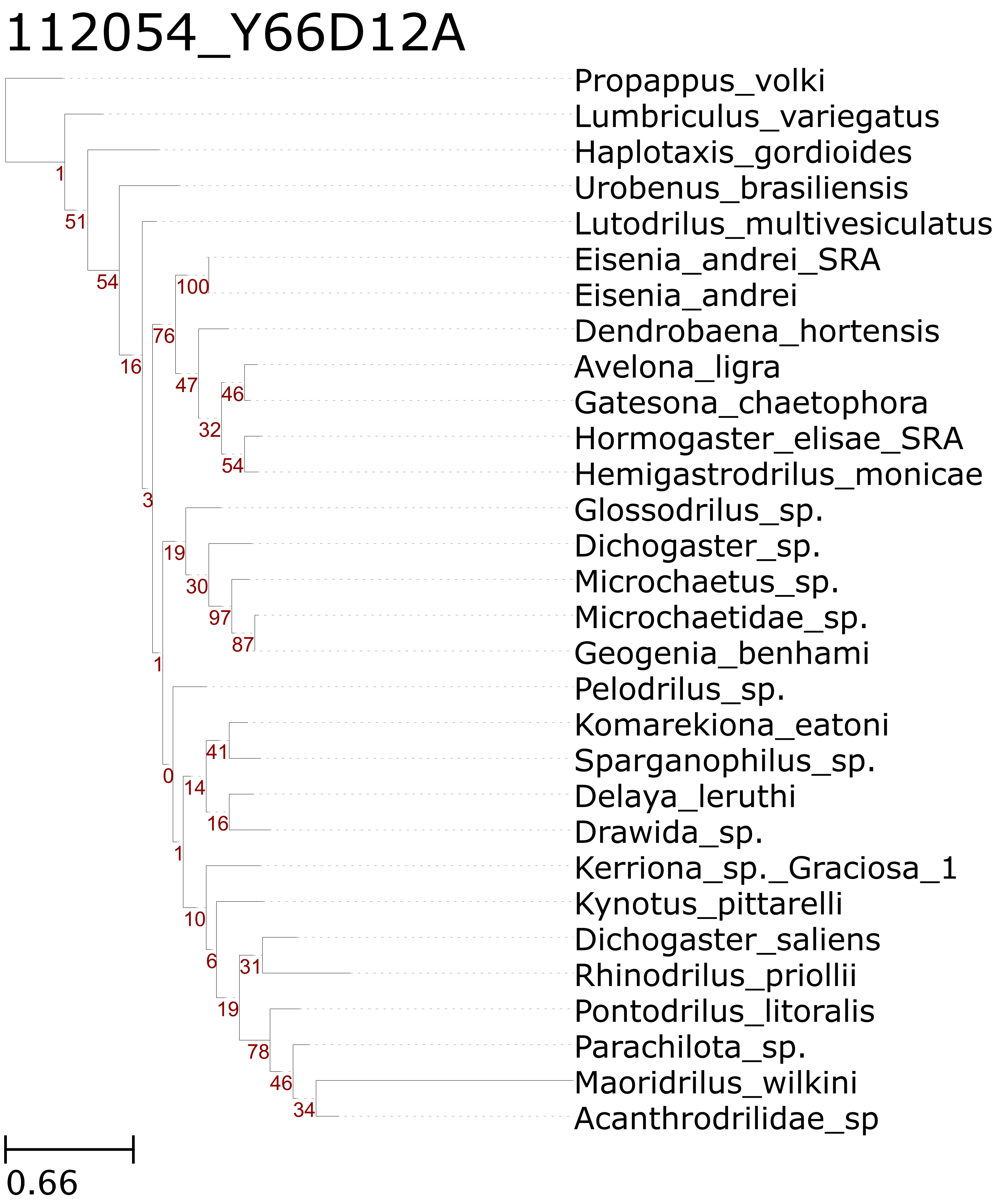

112054_Y66D12A
Propappus_volki
Lumbriculus_variegatus
Haplotaxis_gordioides
Urobenus_brasiliensis
Lutodrilus_multivesiculatus
Eisenia_andrei_SRA
Eisenia_andrei
Dendrobaena_hortensis
Avelona_ligra
Gatesona_chaetophora
Hormogaster_elisae_SRA
Hemigastrodrilus_monicae
Glossodrilus_sp.
Dichogaster_sp.
Microchaetus_sp.
Microchaetidae_sp.
Geogenia_benhami
Pelodrilus_sp.
Komarekiona_eatoni
Sparganophilus_sp.
Delaya_leruthi
Drawida_sp.
Kerriona_sp._Graciosa_1
Kynotus_pittarelli
Dichogaster_saliens
Rhinodrilus_priollii
Pontodrilus_litoralis
Parachilota_sp.
Maoridrilus_wilkini
Acanthrodrilidae_sp
1
51
54
16
3
76
100
47
46
32
54
19
30
97
87
1
0
41
14
16
1
10
6
31
19
78
46
34
0.66

112088_W09C5

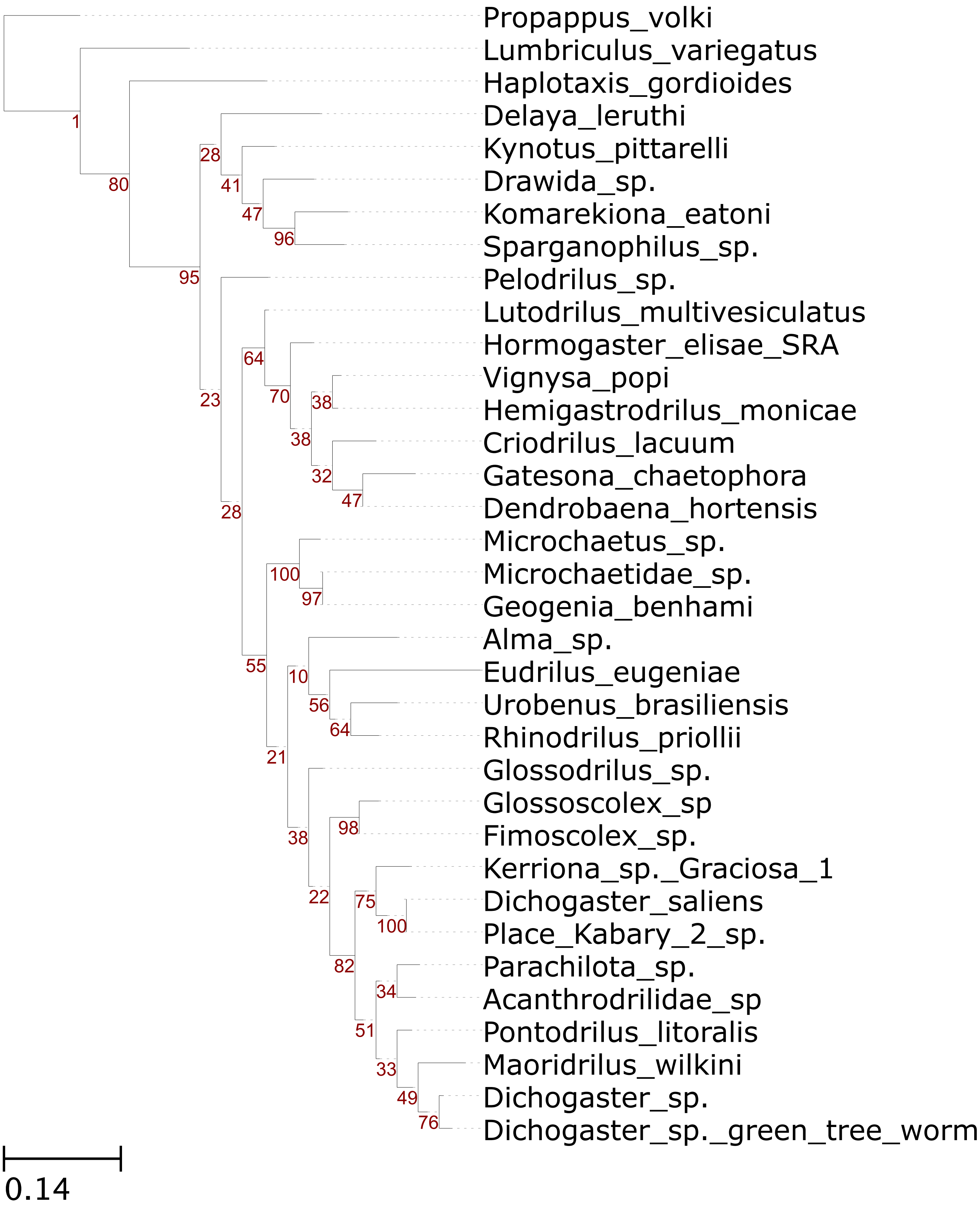

112113_C35D10

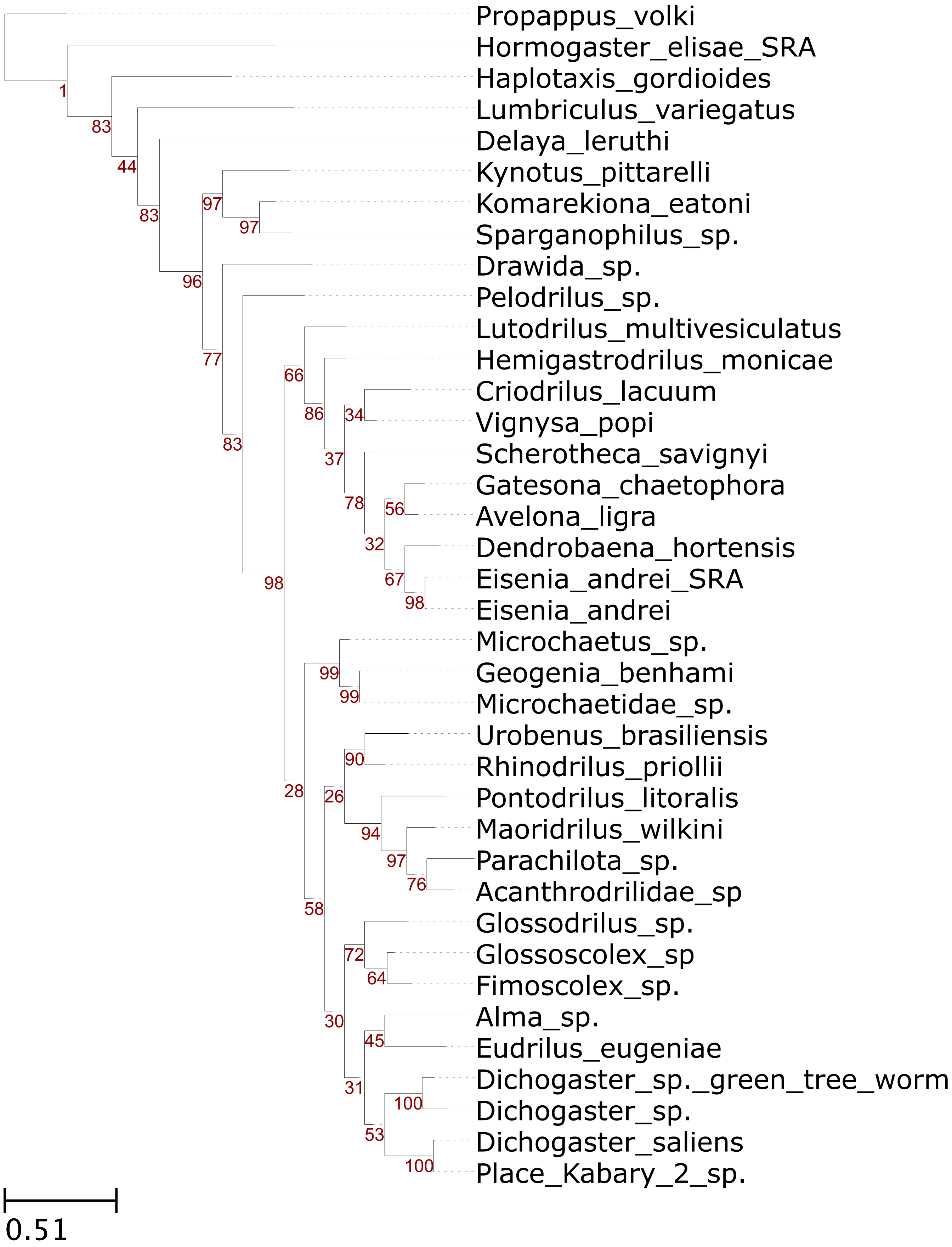

112126_F26A3

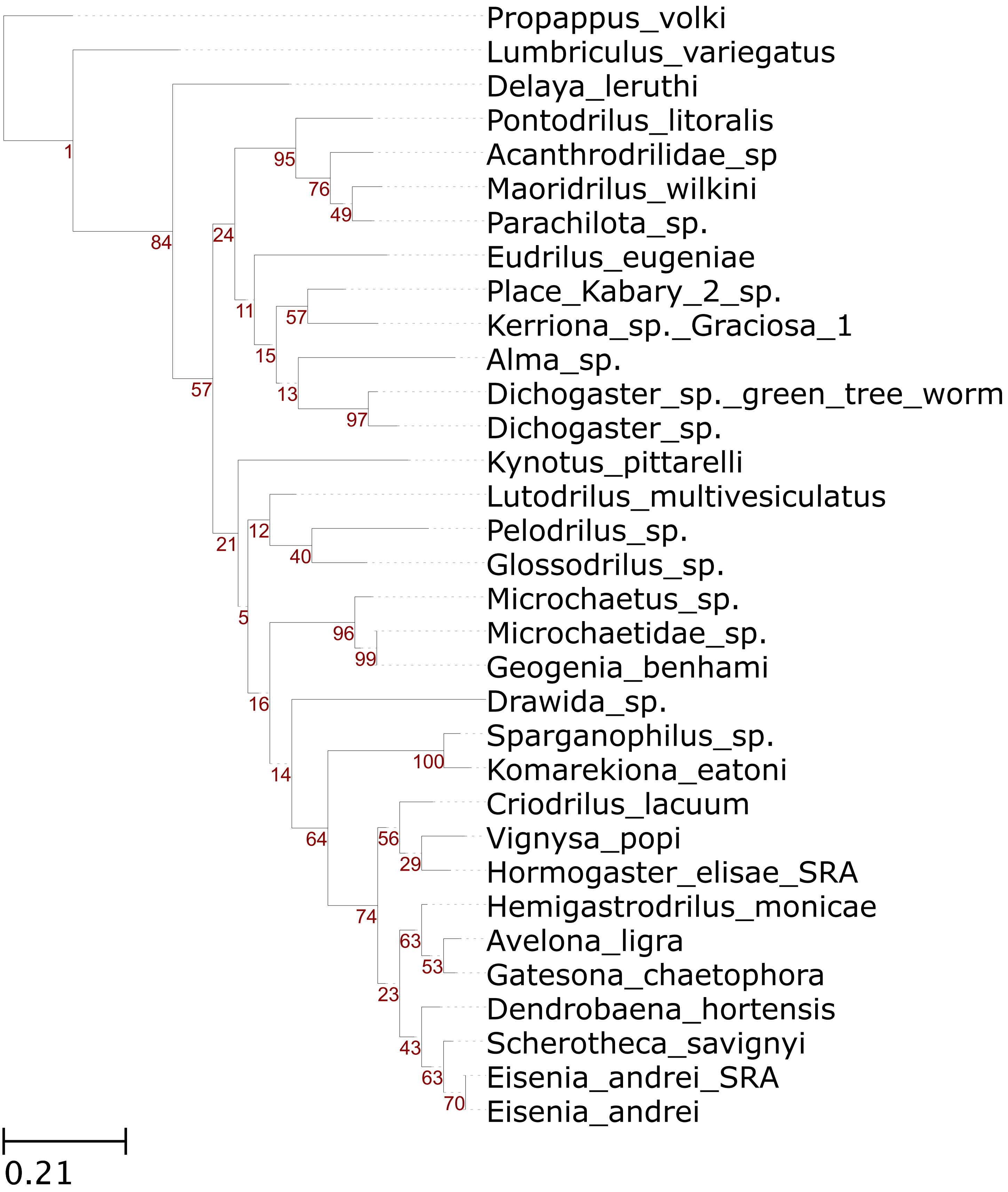

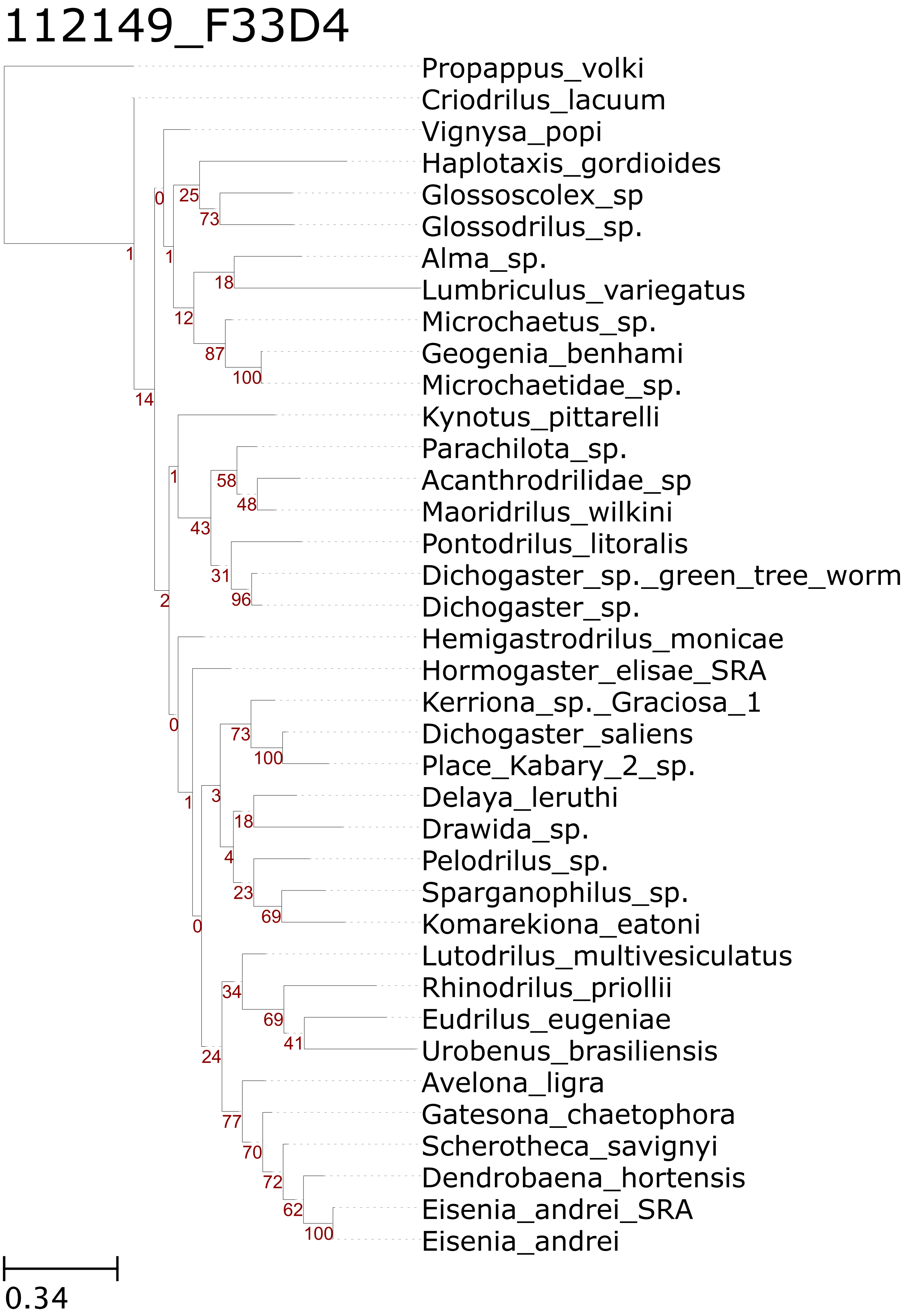
112149_F33D4
Propappus_volki
Criodrilus_lacuum
Vignysa_popi
Haplotaxis_gordioides
Glossoscolex_sp
Glossodrilus_sp.
Alma_sp.
Lumbriculus_variegatus
Microchaetus_sp.
Geogenia_benhami
Microchaetidae_sp.
Kynotus_pittarelli
Parachilota_sp.
Acanthrodrilidae_sp
Maoridrilus_wilkini
Pontodrilus_litoralis
Dichogaster_sp._green_tree_worm
Dichogaster_sp.
Hemigastrodrilus_monicae
Hormogaster_elisae_SRA
Kerriona_sp._Graciosa_1
Dichogaster_saliens
Place_Kabary_2_sp.
Delaya_leruthi
Drawida_sp.
Pelodrilus_sp.
Sparganophilus_sp.
Komarekiona_eatoni
Lutodrilus_multivesiculatus
Rhinodrilus_priollii
Eudrilus_eugeniae
Urobenus_brasiliensis
Avelona_ligra
Gatesona_chaetophora
Scherotheca_savignyi
Dendrobaena_hortensis
Eisenia_andrei_SRA
Eisenia_andrei
0.34

112180_W04D2

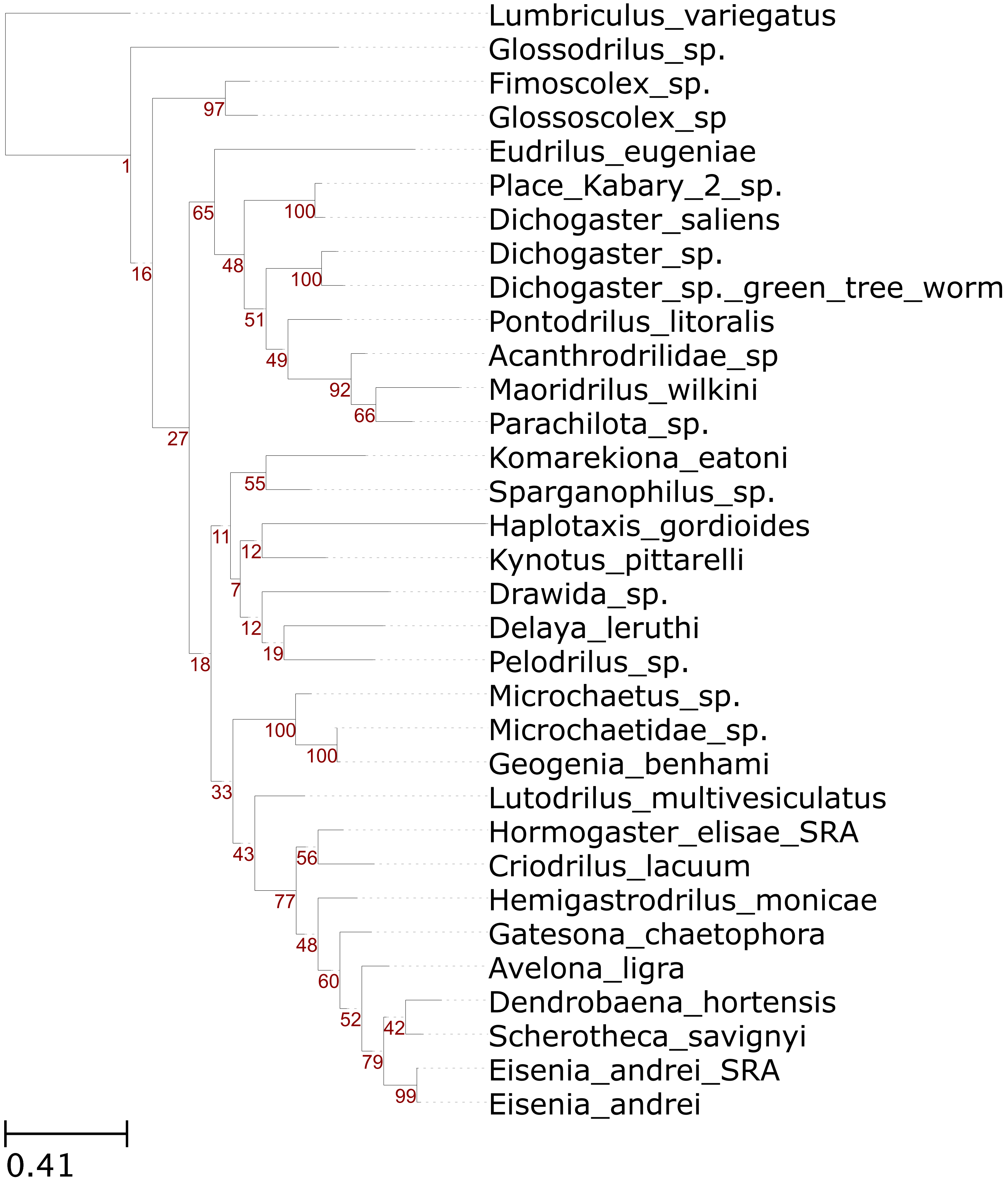

112197_Y54E5B

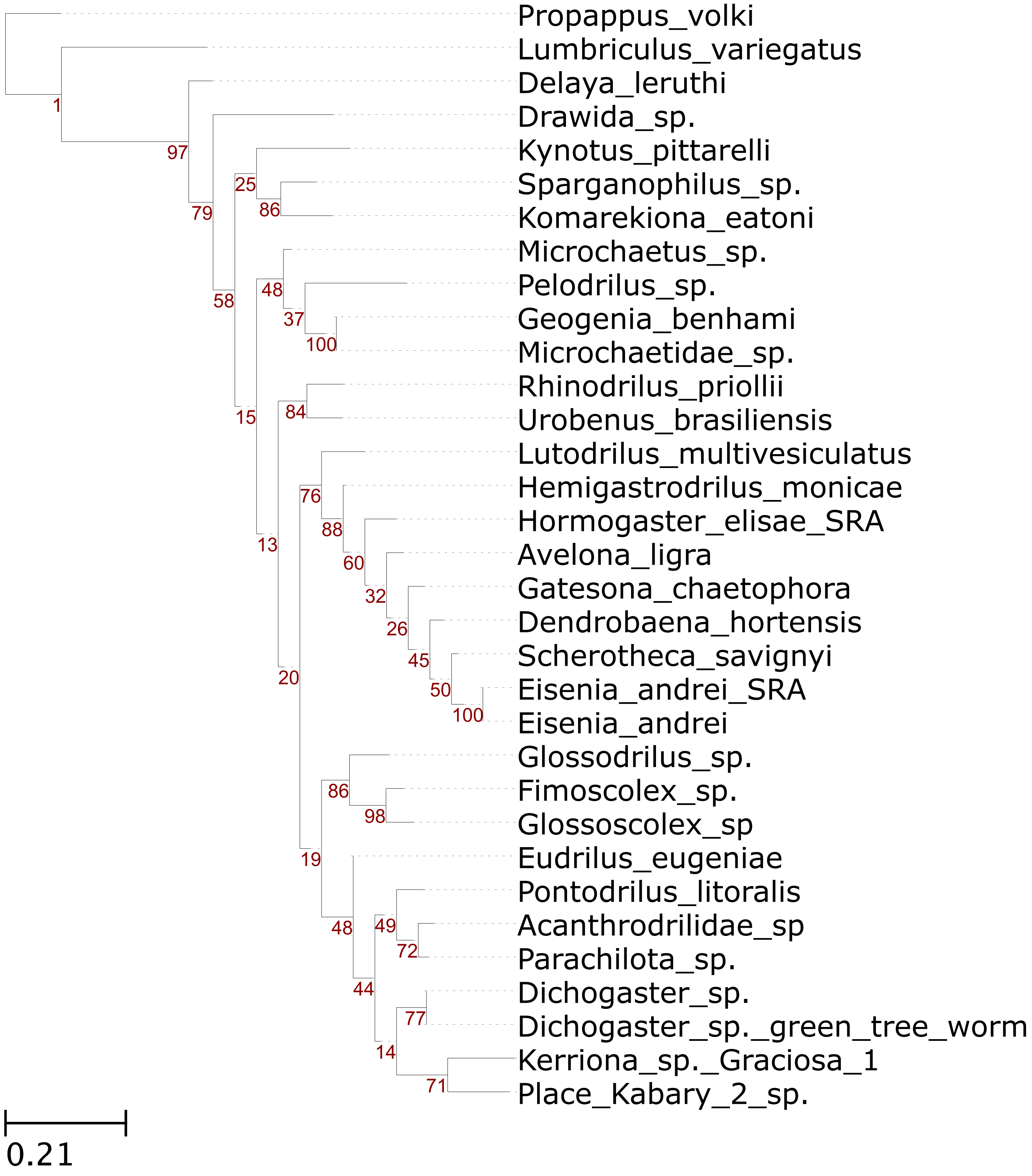

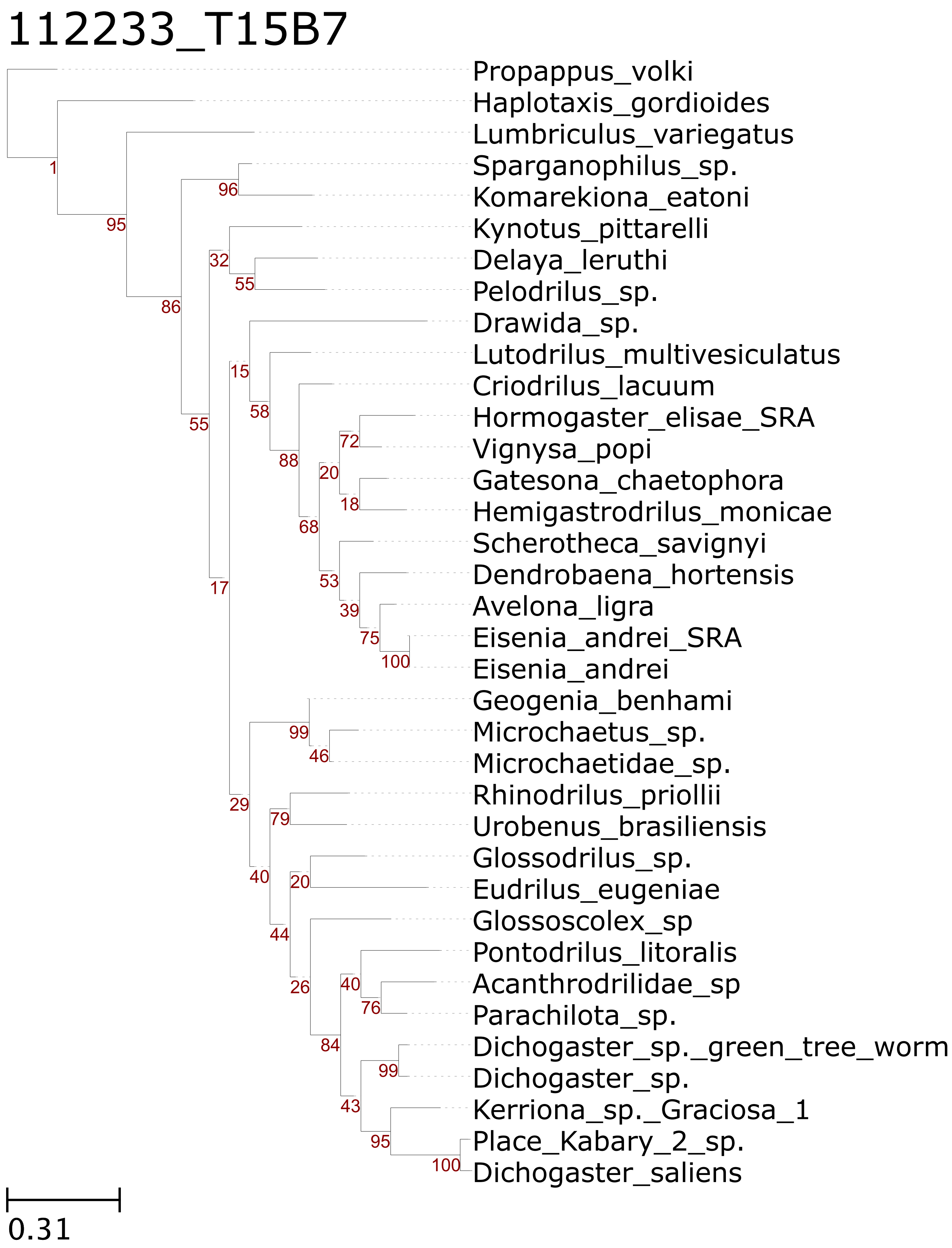
112233_T15B7
Propappus_volki
Haplotaxis_gordioides
Lumbriculus_variegatus
Sparganophilus_sp.
Komarekiona_eatoni
Kynotus_pittarelli
Delaya_leruthi
Pelodrilus_sp.
Drawida_sp.
Lutodrilus_multivesiculatus
Criodrilus_lacuum
Hormogaster_elisae_SRA
Vignysa_popi
Gatesona_chaetophora
Hemigastrodrilus_monicae
Scherotheca_savignyi
Dendrobaena_hortensis
Avelona_ligra
Eisenia_andrei_SRA
Eisenia_andrei
Geogenia_benhami
Microchaetus_sp.
Microchaetidae_sp.
Rhinodrilus_priollii
Urobenus_brasiliensis
Glossodrilus_sp.
Eudrilus_eugeniae
Glossoscolex_sp
Pontodrilus_litoralis
Acanthrodrilidae_sp
Parachilota_sp.
Dichogaster_sp._green_tree_worm
Dichogaster_sp.
Kerriona_sp._Graciosa_1
Place_Kabary_2_sp.
Dichogaster_saliens
1
95
86
96
55
32
55
15
58
88
72
20
18
68
53
39
75
100
17
99
46
29
79
40
20
44
26
40
76
84
99
43
95
100
0.31

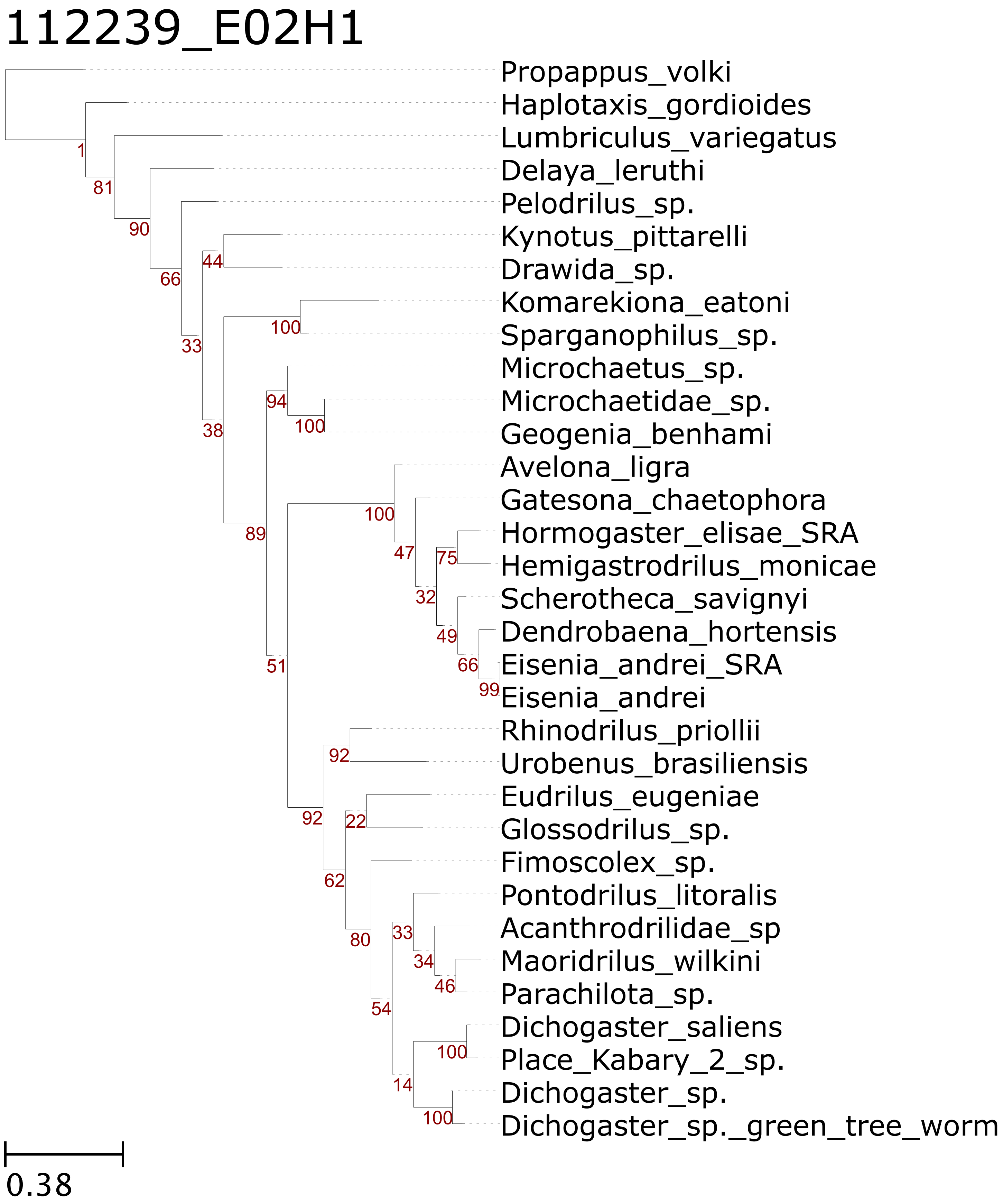
112239_E02H1
Propappus_volki
Haplotaxis_gordioides
Lumbriculus_variegatus
Delaya_leruthi
Pelodrilus_sp.
Kynotus_pittarelli
Drawida_sp.
Komarekiona_eatoni
Sparganophilus_sp.
Microchaetus_sp.
Microchaetidae_sp.
Geogenia_benhami
Avelona_ligra
Gatesona_chaetophora
Hormogaster_elisae_SRA
Hemigastrodrilus_monicae
Scherotheca_savignyi
Dendrobaena_hortensis
Eisenia_andrei_SRA
Eisenia_andrei
Rhinodrilus_priollii
Urobenus_brasiliensis
Eudrilus_eugeniae
Glossodrilus_sp.
Fimoscolex_sp.
Pontodrilus_litoralis
Acanthrodrilidae_sp
Maoridrilus_wilkini
Parachilota_sp.
Dichogaster_saliens
Place_Kabary_2_sp.
Dichogaster_sp.
Dichogaster_sp._green_tree_worm
1
81
90
66
44
33
100
38
94
100
89
100
47
75
32
49
66
99
51
92
92
22
62
80
33
34
46
54
100
14
100
0.38

112270_D2096

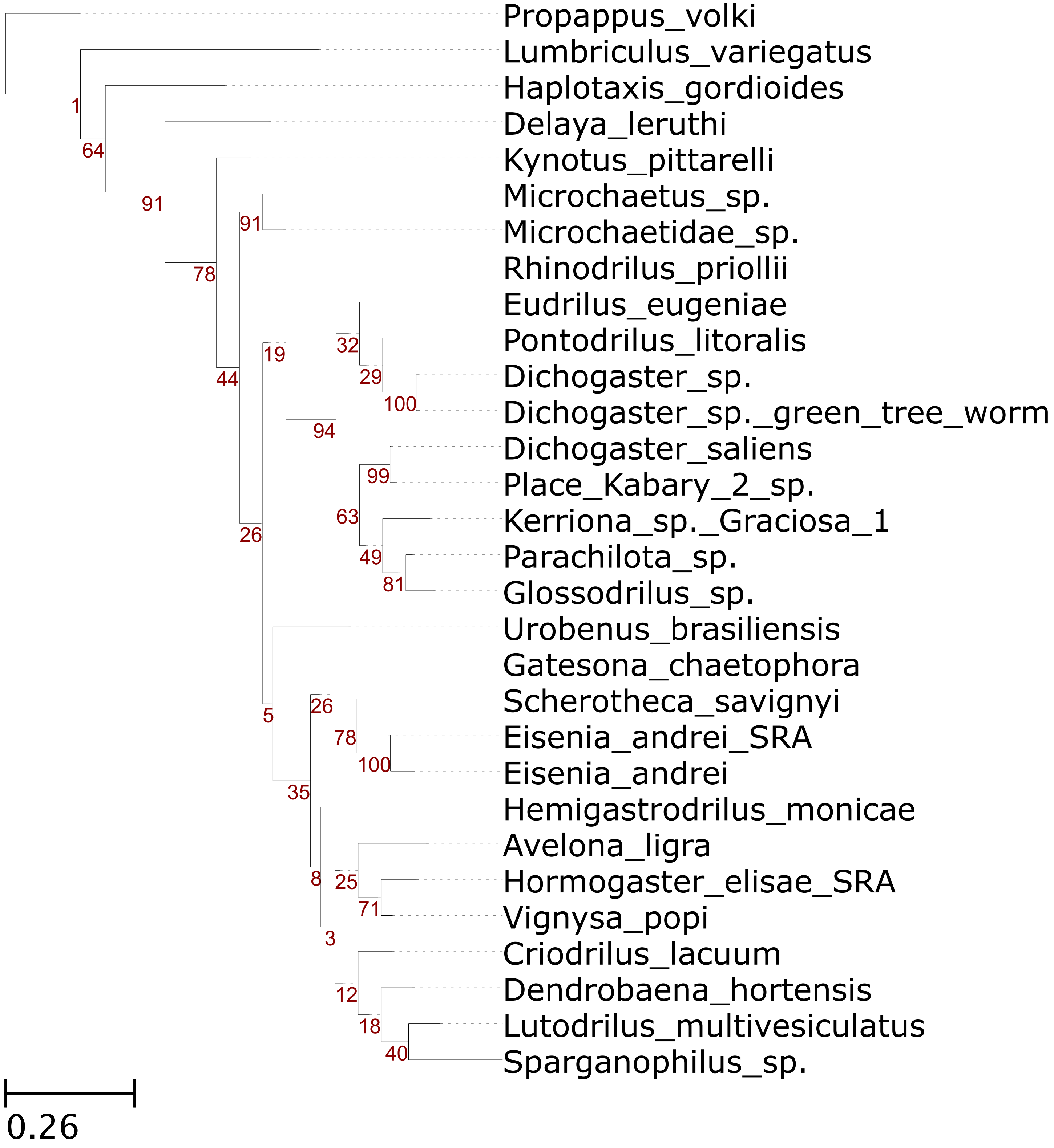

112312_F19B6

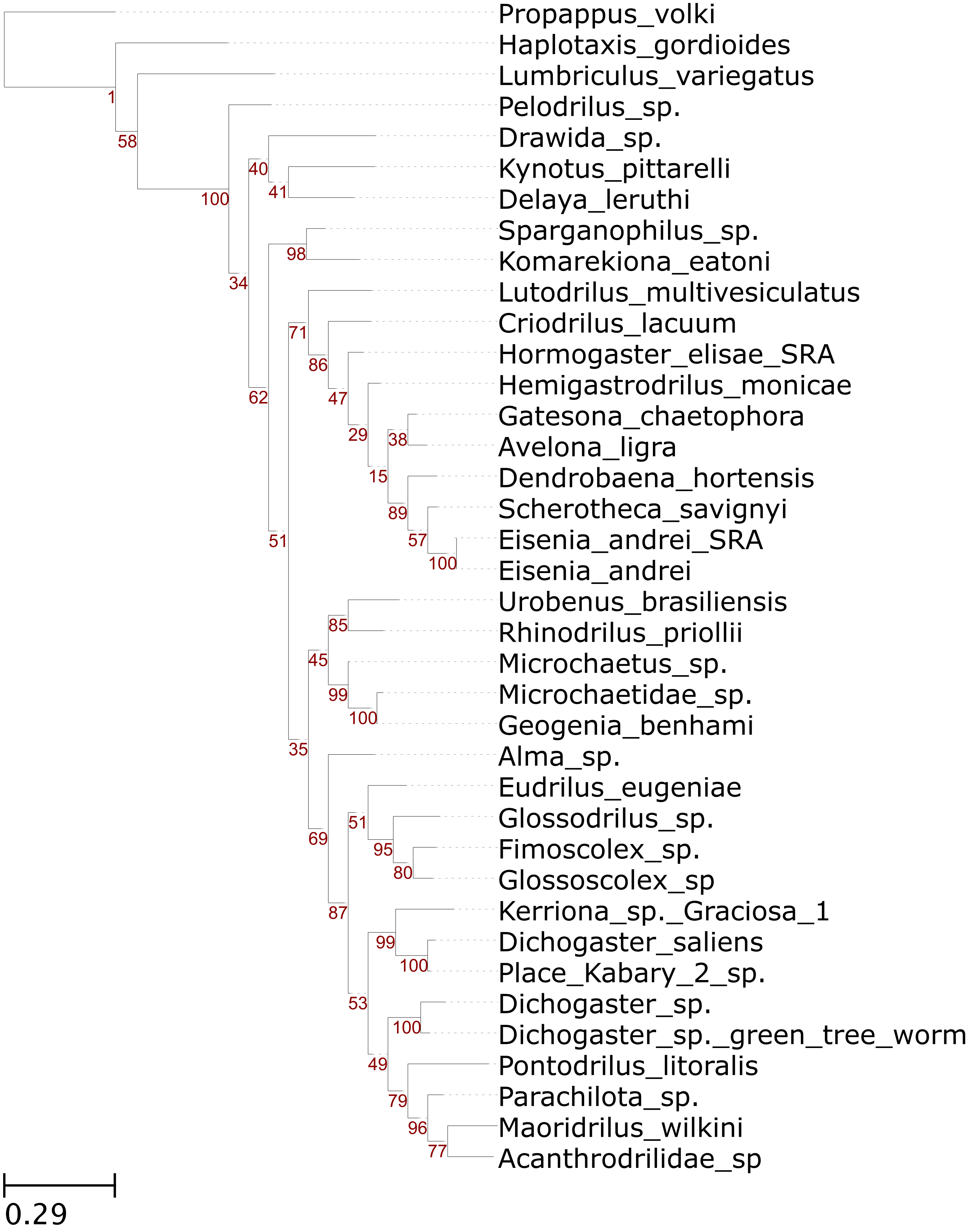

112319_W02A11

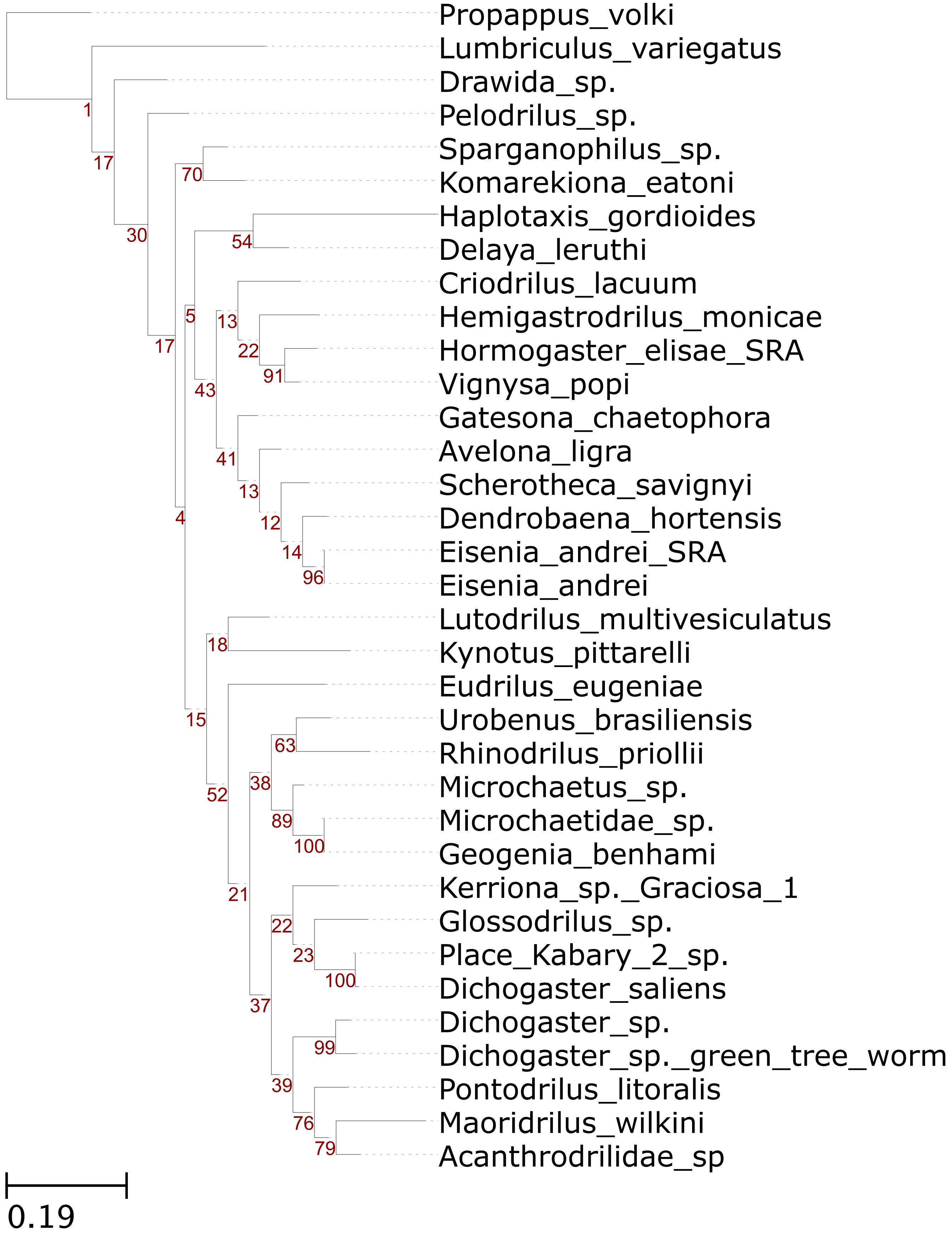

112324_C05D11

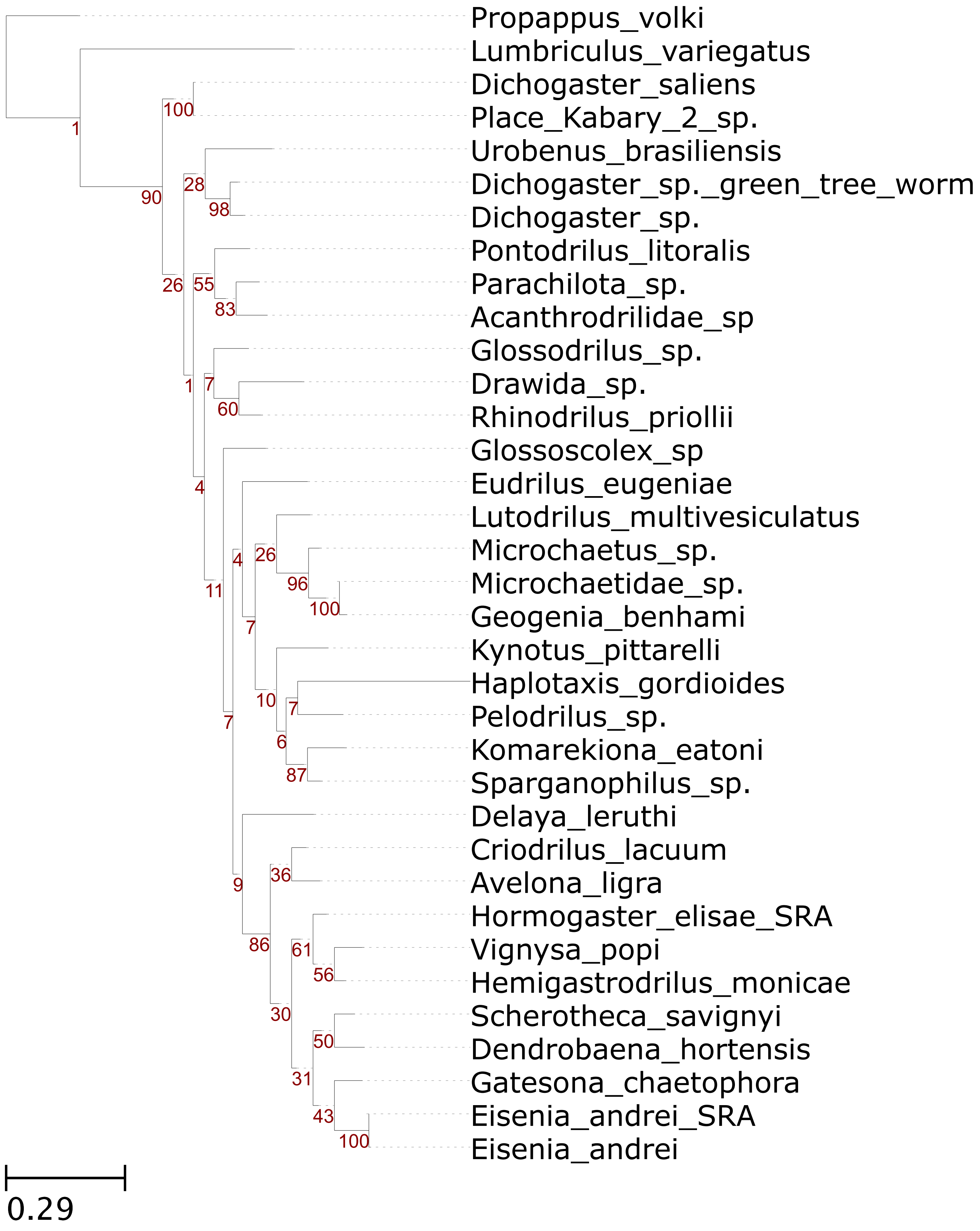

112345_R12H7

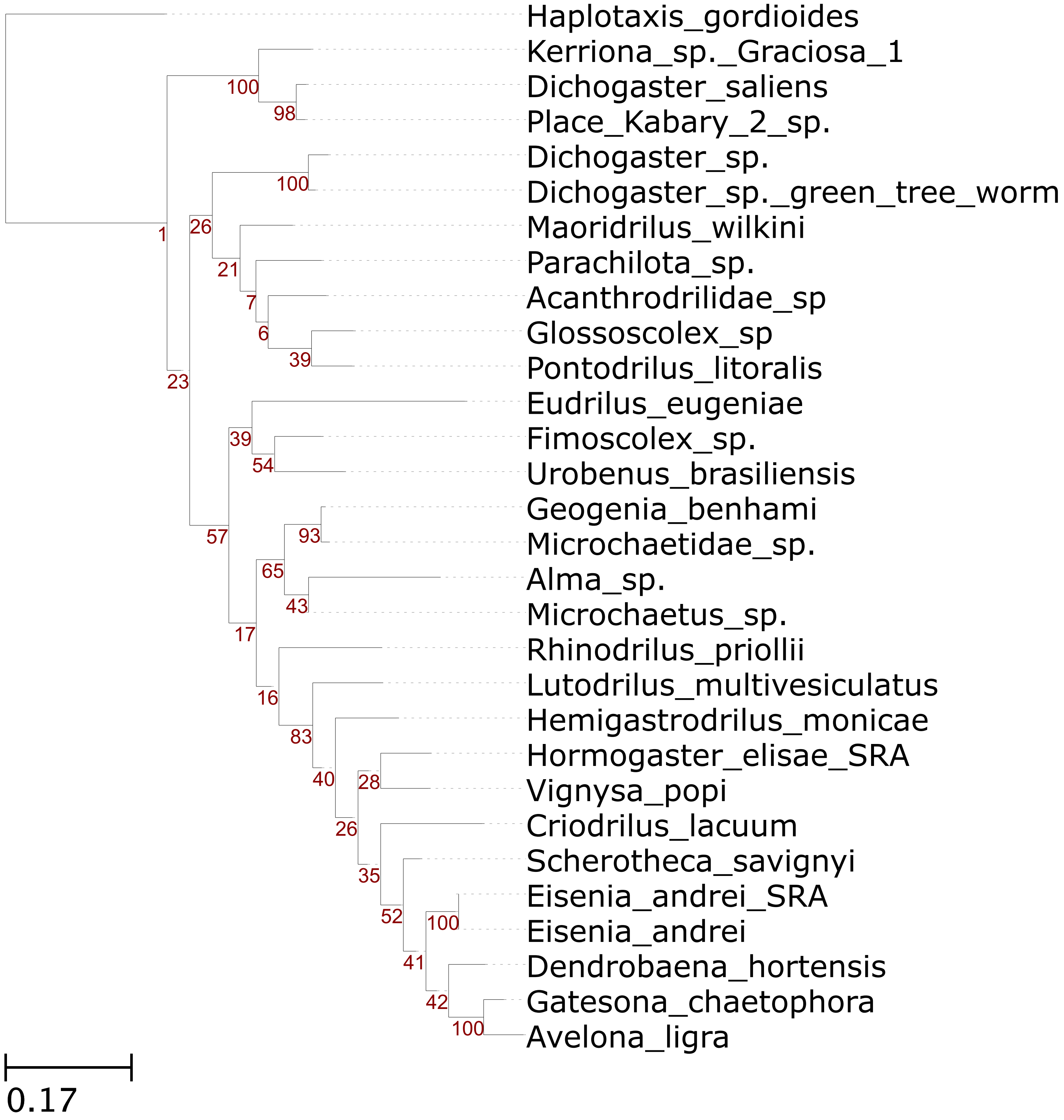

111678_Y82E9BR

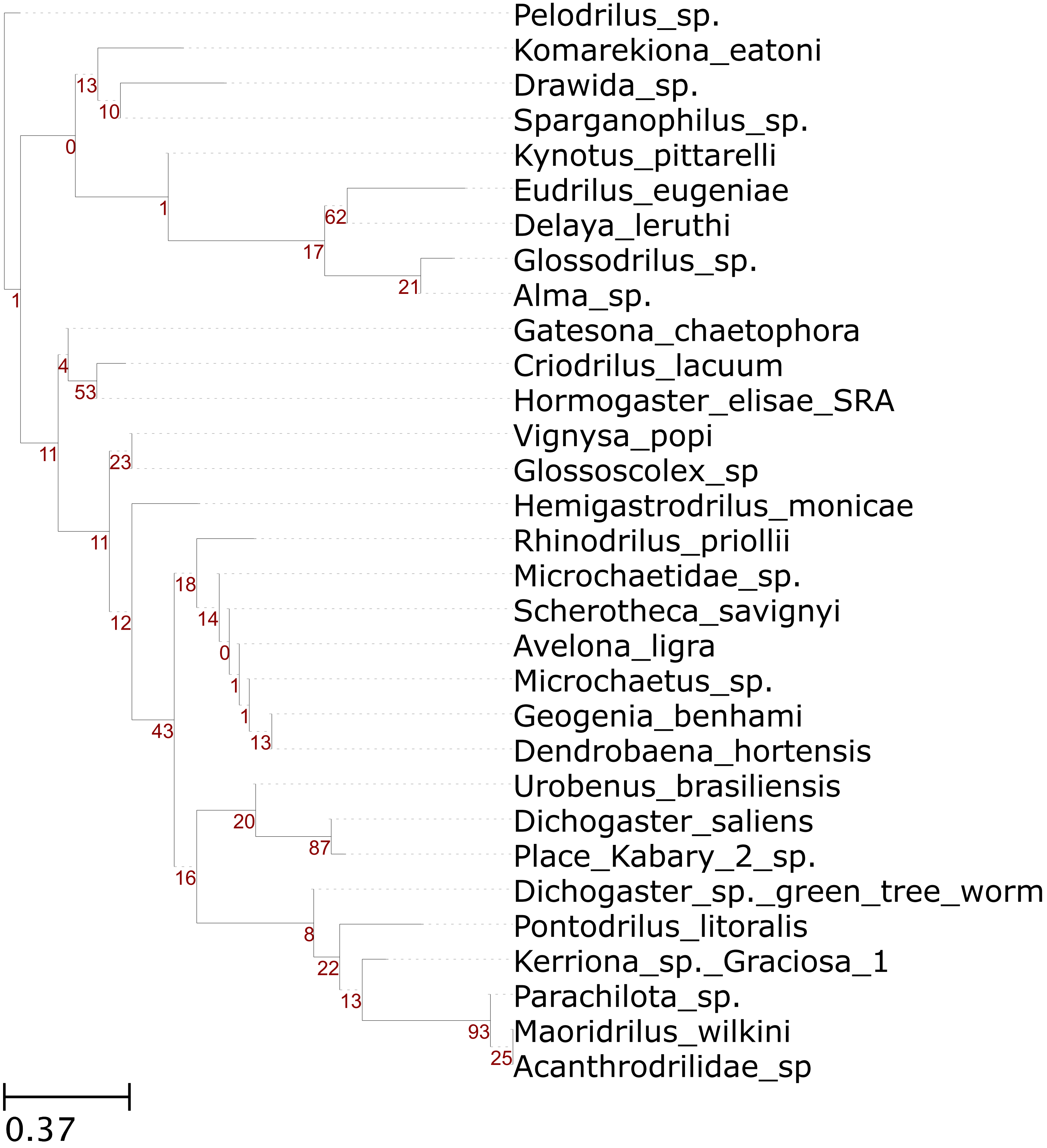

112116_C25A1

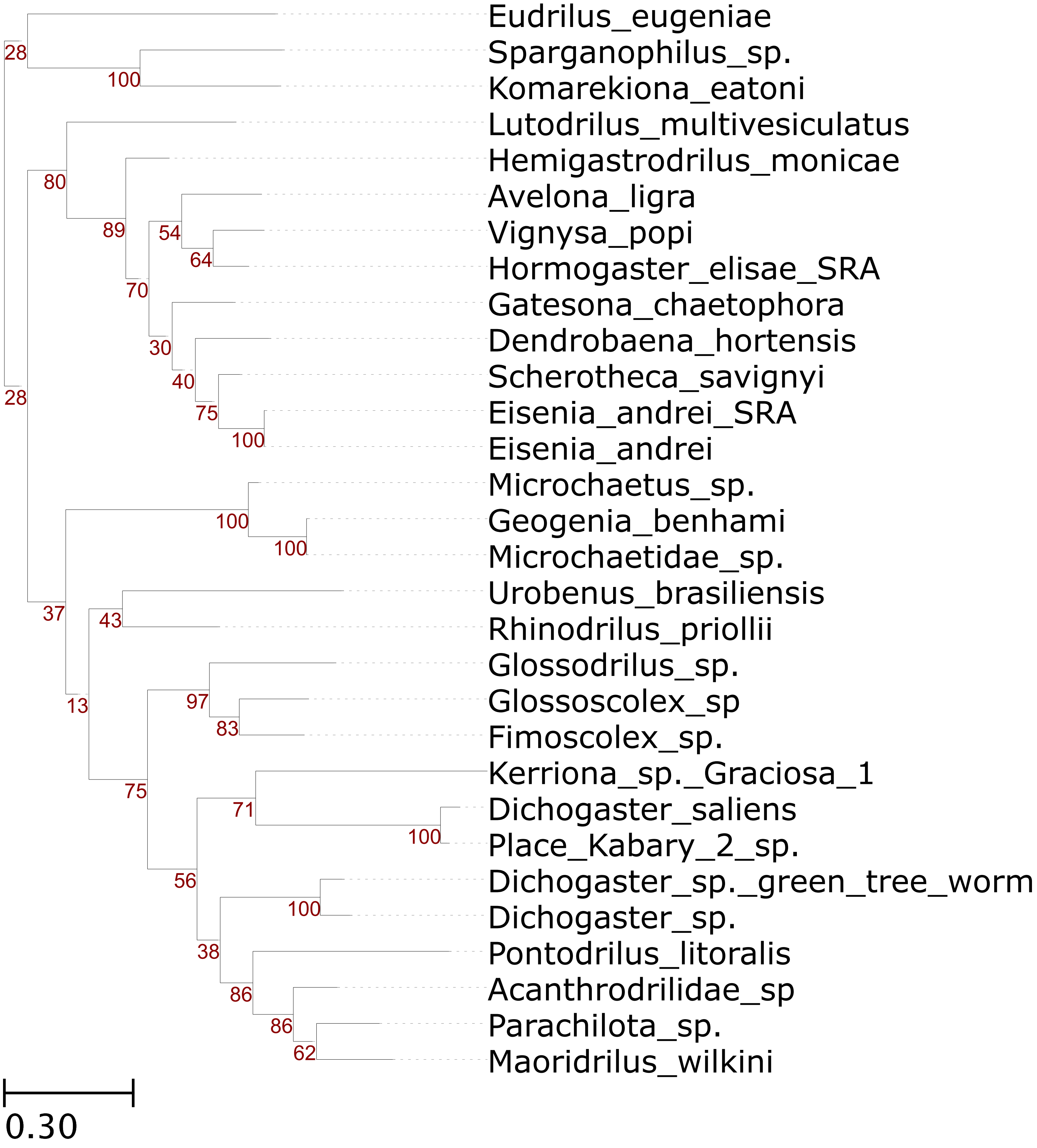

111525_C01A2

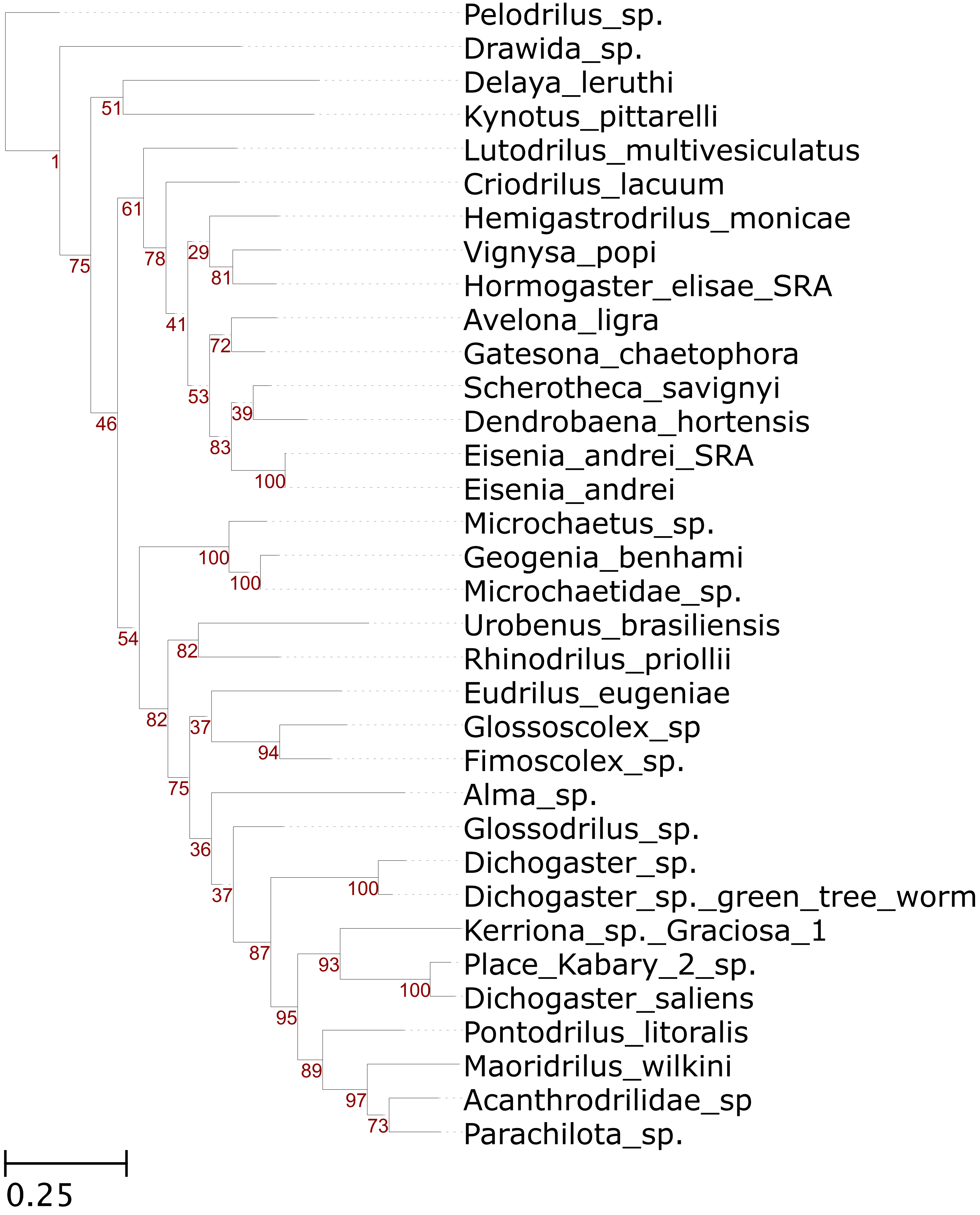

Supplement: Supplementary file 2 — ML phylograms with bootstrap support values of all 55 genes (OGs) that were used to construct the 75% data matrix from which “?Haplotaxidae sp.” had been excluded. These genes passed through the TreSpEx and BaCoCa filters described in the text, and included all sites (i.e., sites comprising >50% gaps were not deleted). The title for each tree lists the tree number, the orthogroup number in the HaMStR Lophotrochozoa core ortholog set (e.g., “111,230” for tree 1) and the gene/transcript name (e.g., “C43H8.2” for tree 1). The gene/transcript name can be looked up in online databases (e.g., EnsemblMetazoa; http://metazoa.ensembl.org). For example, for the first tree, C43H8 is a transcript of WBGene00016622, repressor of RNA polymerase III transcription MAF1. (PDF 14509 kb) [file 12862_2017_973_MOESM2_ESM.pdf]
