# Supplementary figures and images for: Phylogenomic analyses of Crassiclitellata support major Northern and Southern Hemisphere clades and a Pangaean origin for earthworms
Source: BMC Evol Biol. 2017 May 30;17:123. doi: 10.1186/s12862-017-0973-4 (PMC5450073; doi:10.1186/s12862-017-0973-4)

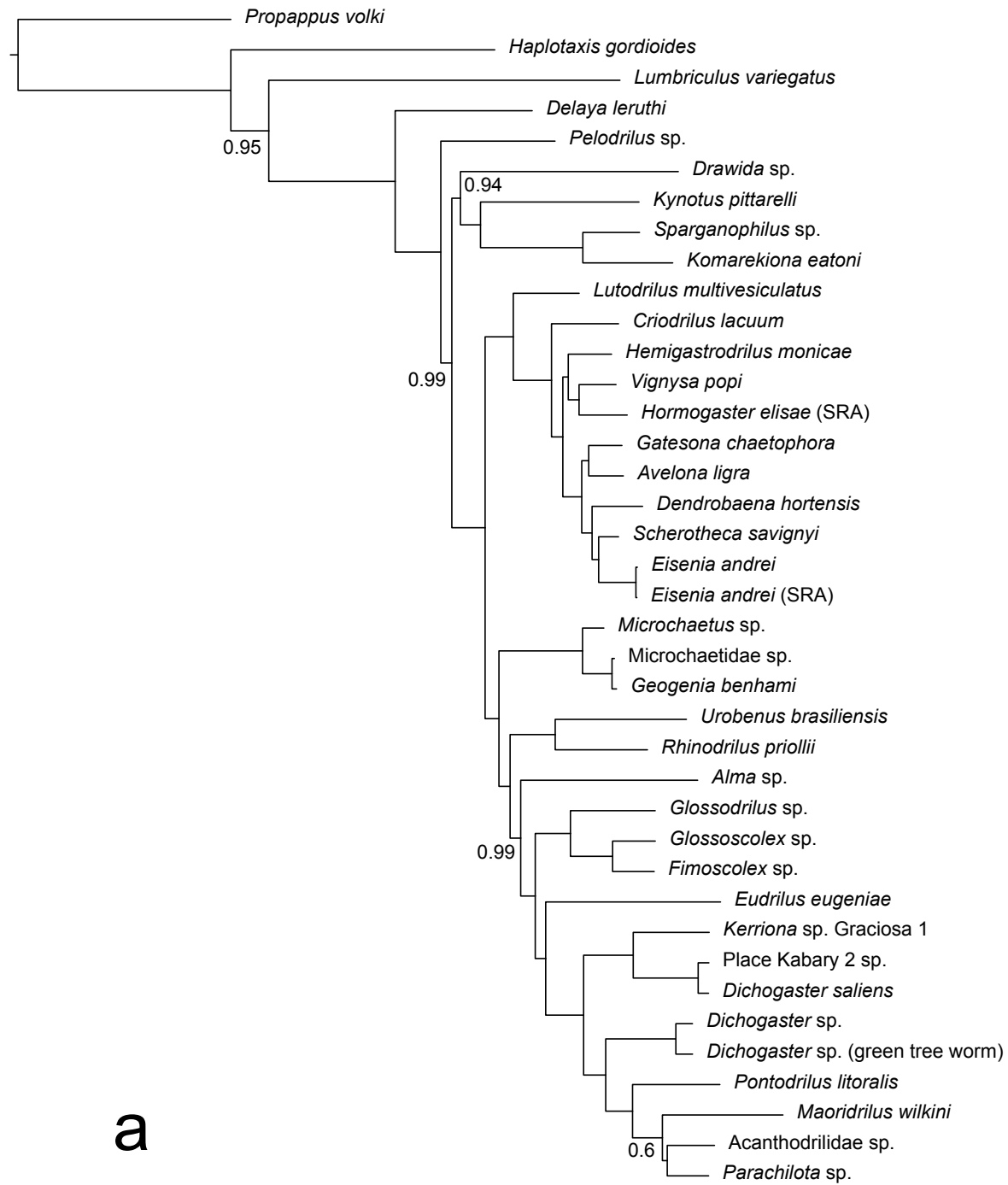

a

0.3

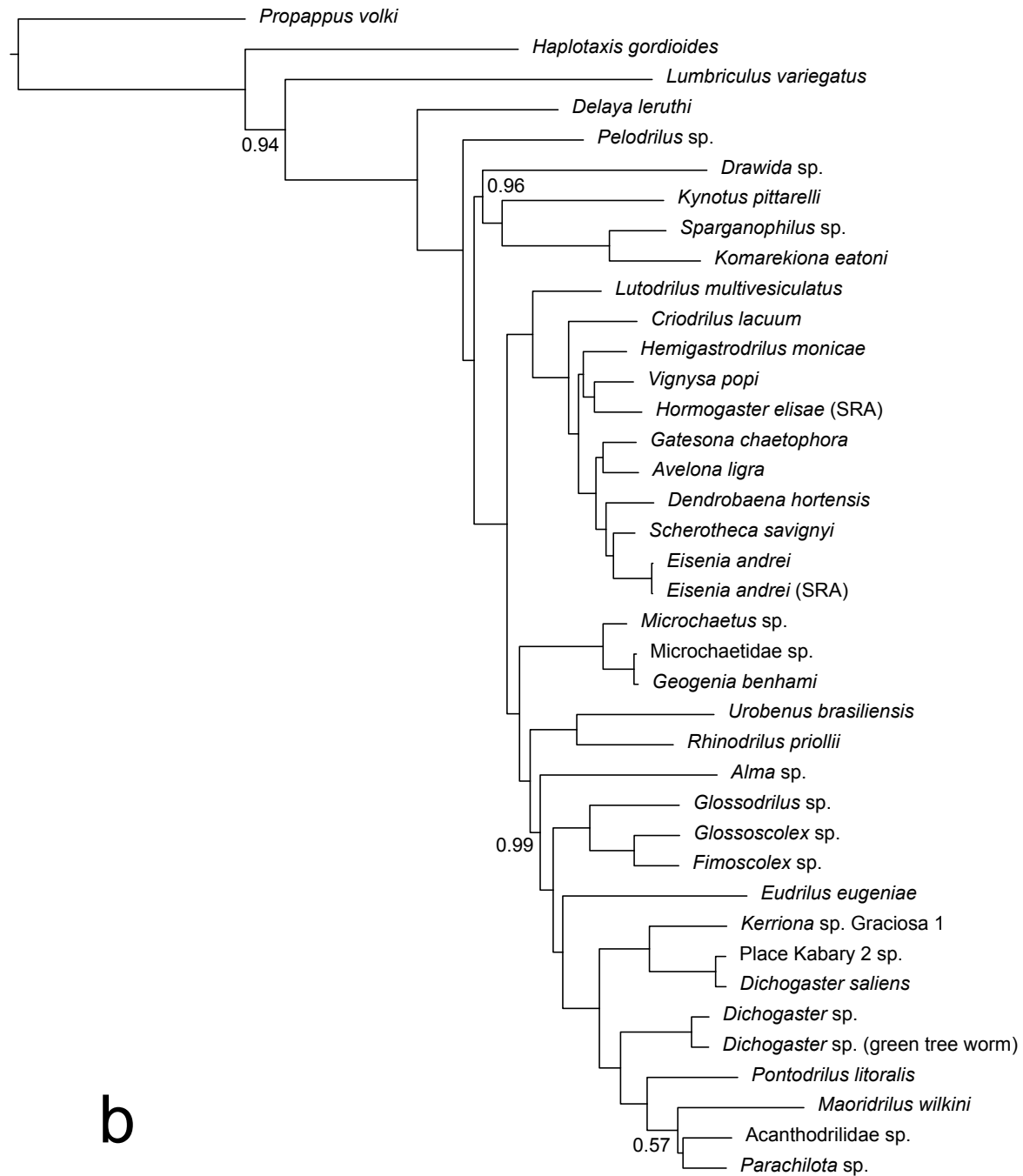

Supplement: Supplementary file 3 — PhyloBayes 50%-majority-rule consensus phylogram for a) the 75% matrix with ?Haplotaxidae sp. removed, filtered with TreSpEx and BaCoCa, all sites, and b) 75% matrix with ?Haplotaxidae sp. removed, filtered with TreSpEx and BaCoCa, and sites comprising >50% gaps deleted. Posterior probabilities are shown at nodes; nodes without values have posterior probabilities of 1.0. (PDF 82 kb) [file 12862_2017_973_MOESM3_ESM.pdf]

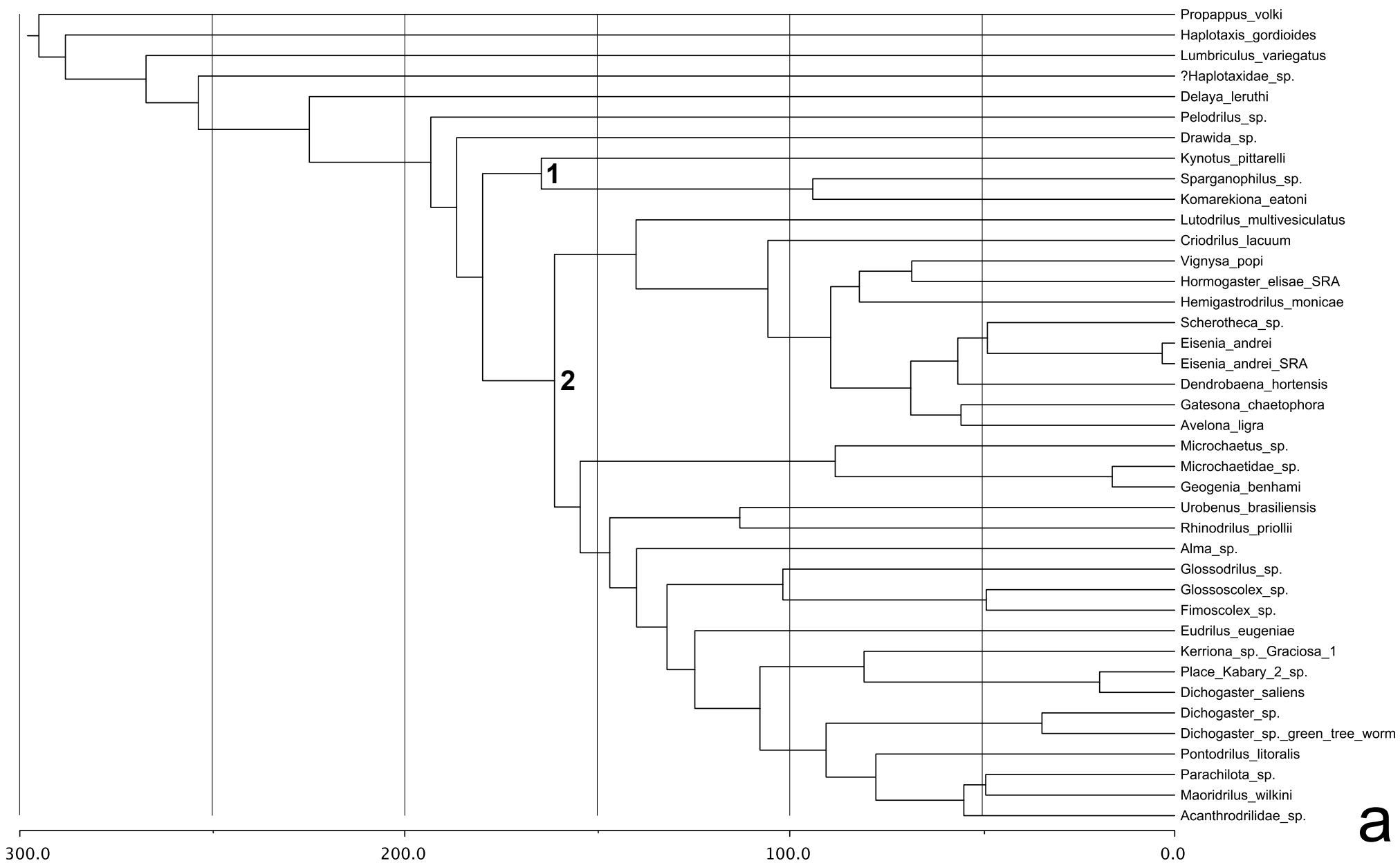

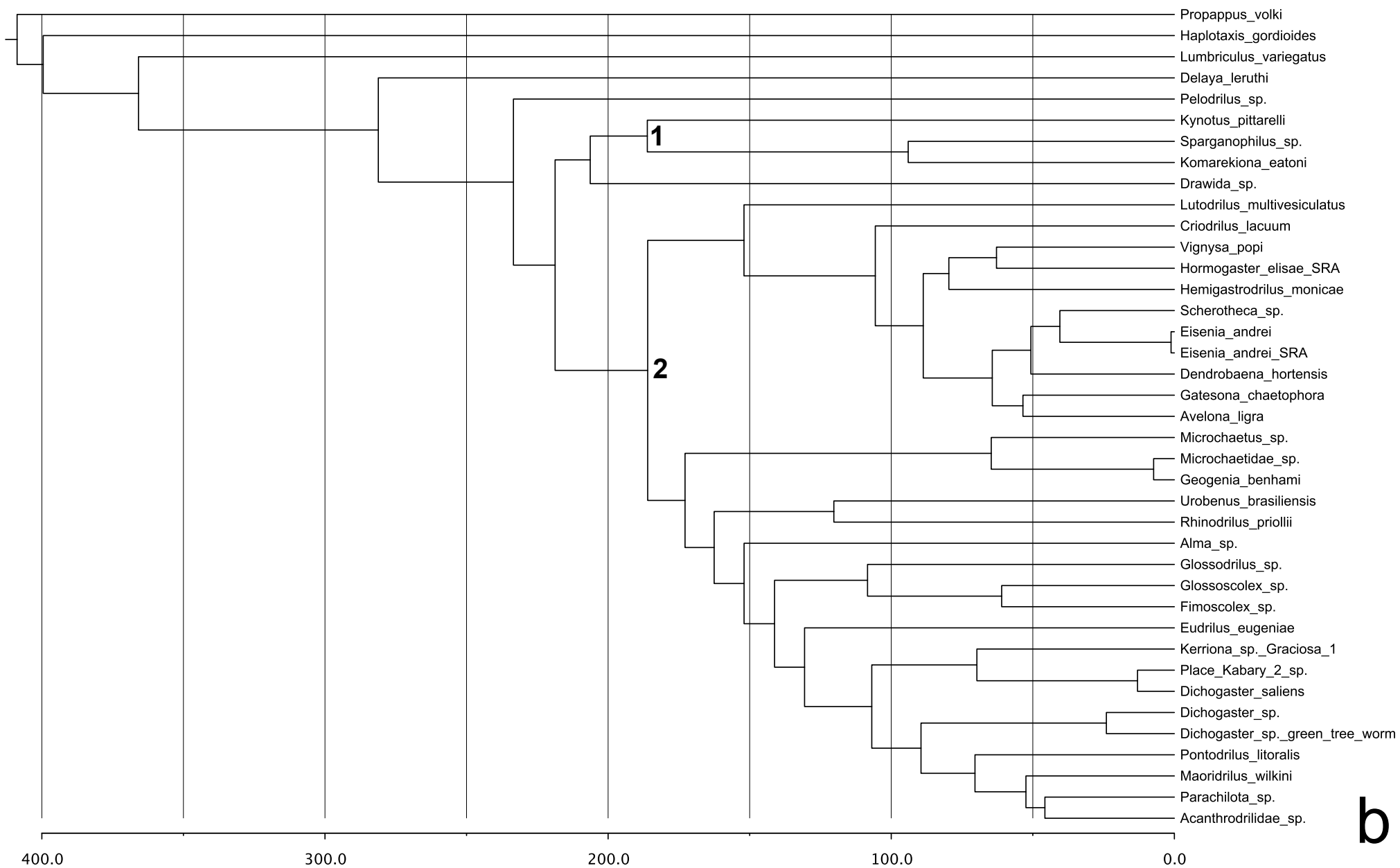

**b**

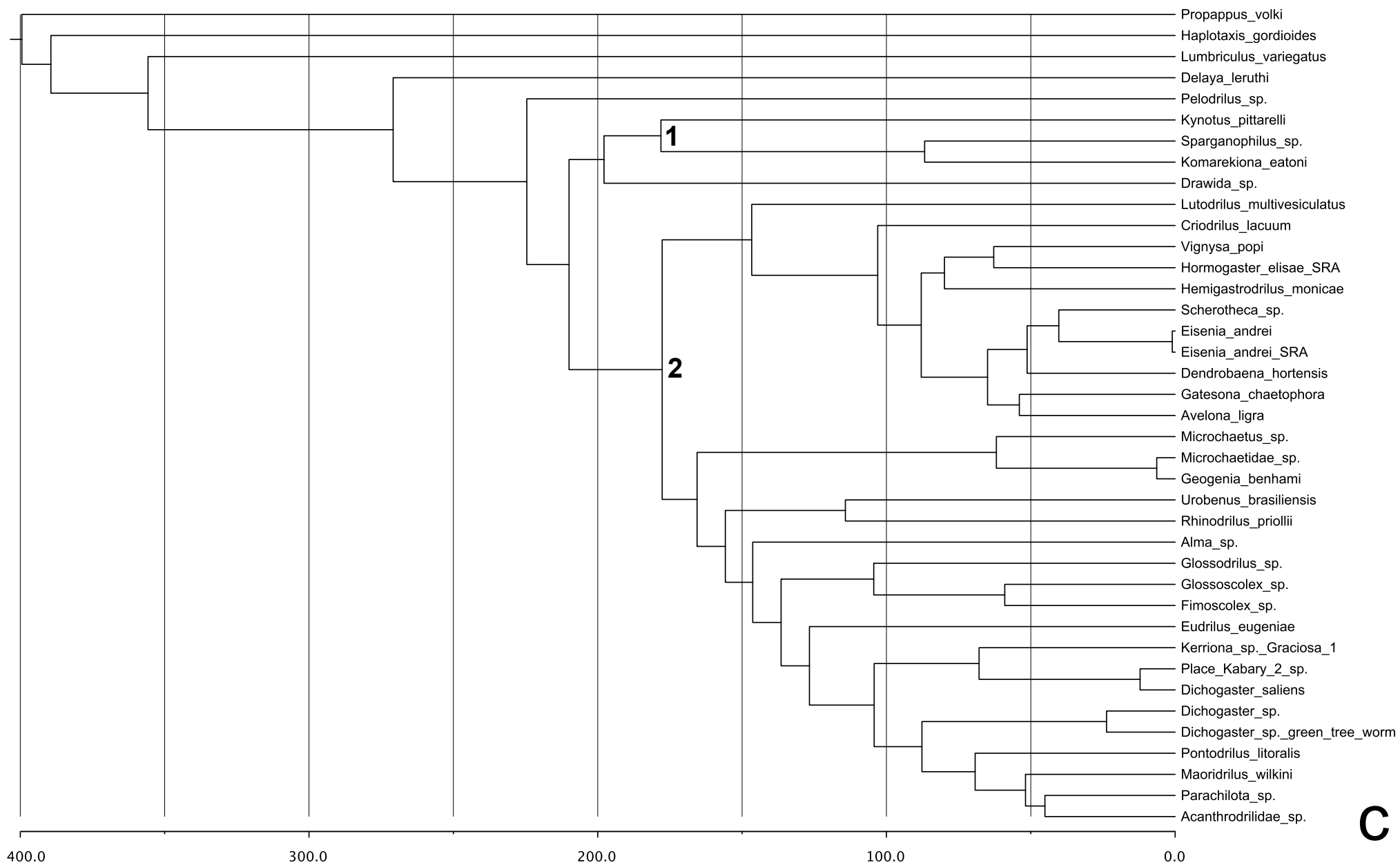

C

Supplement: Supplementary file 4 — Chronograms depicting results of PhyloBayes dating analyses for three 75% data matrices, with highlighted nodes separating Kynotus from Sparganophilus + Komarekiona (node 1) and separating the Northern Hemisphere clade comprising Lutodrilus and Lumbricoidea and the clade comprising several Southern Hemisphere families (node 2). Scale bars in millions of years ago. a) Unfiltered 75% matrix including ?Haplotaxidae sp., all sites included b), 75% matrix with ?Haplotaxidae sp. removed, filtered with TreSpEx and BaCoCa, all sites, and c) 75% matrix with ?Haplotaxidae sp. removed, filtered with TreSpEx and BaCoCa, and sites comprising >50% gaps deleted. (PDF 779 kb) [file 12862_2017_973_MOESM4_ESM.pdf]
